# Influence of genome-scale RNA structure disruption on the replication of murine norovirus—similar replication kinetics in cell culture but attenuation of viral fitness *in vivo*

**DOI:** 10.1093/nar/gkt334

**Published:** 2013-04-27

**Authors:** Nora McFadden, Armando Arias, Inga Dry, Dalan Bailey, Jeroen Witteveldt, David J. Evans, Ian Goodfellow, Peter Simmonds

**Affiliations:** ^1^Division of Infection and Immunity, Roslin Institute, University of Edinburgh, Easter Bush, Edinburgh EH25 9RG, UK ^2^Calicivirus Research Group, Department of Virology, Faculty of Medicine, Imperial College London, St Mary's Campus, Norfolk Place, London W2 1PG, UK, ^3^Division of Virology, Department of Pathology, University of Cambridge, Addenbrooke's Hospital, Hills Road, Cambridge CB2 2QQ, UK and ^4^School of Life Sciences, University of Warwick, Coventry CV4 7AL, UK

## Abstract

Mechanisms by which certain RNA viruses, such as hepatitis C virus, establish persistent infections and cause chronic disease are of fundamental importance in viral pathogenesis. Mammalian positive-stranded RNA viruses establishing persistence typically possess genome-scale ordered RNA secondary structure (GORS) in their genomes. Murine norovirus (MNV) persists in immunocompetent mice and provides an experimental model to functionally characterize GORS. Substitution mutants were constructed with coding sequences in NS3/4- and NS6/7-coding regions replaced with sequences with identical coding and (di-)nucleotide composition but disrupted RNA secondary structure (F1, F2, F1/F2 mutants). Mutants replicated with similar kinetics to wild-type (WT) MNV3 in RAW264.7 cells and primary macrophages, exhibited similar (highly restricted) induction and susceptibility to interferon-coupled cellular responses and equal replication fitness by serial passaging of co-cultures. *In vivo*, both WT and F1/F2 mutant viruses persistently infected mice, although F1, F2 and F1/F2 mutant viruses were rapidly eliminated 1–7 days post-inoculation in competition experiments with WT. F1/F2 mutants recovered from tissues at 9 months showed higher synonymous substitution rates than WT and nucleotide substitutions that potentially restored of RNA secondary structure. GORS plays no role in basic replication of MNV but potentially contributes to viral fitness and persistence *in vivo*.

## INTRODUCTION

In addition to its coding capacity, RNA virus genomes possess a number of other functional attributes that contribute to genome replication, alternative translation initiation strategies and autocatalytic cleavage reactions. Many of these additional capabilities arise through the formation of secondary and higher-order RNA internally base-paired structures, including internal ribosome entry sites (IRES) that function in ribosome recruitment and the initiation of translation (1). Genome replication and packaging are similarly dependent on RNA structural elements that mediate RNA/RNA and RNA/protein interactions in many virus groups (2–7). Functional RNA structure characterized to date are typically short stem loops, frequently associated, as in the case of many IRESs, with tertiary structure interactions such as pseudoknots.

In a prevailing paradigm in which functional RNA structures are discrete entities, typically at the ends of genomes (replication elements, IRESs) or in intergenic regions (e.g. transcriptional components), it was somewhat unexpected to find that genomes of a number of mammalian and plant RNA viruses contained extensive RNA secondary structure throughout the genome (8). Although initial analyses were bioinformatically based, we have recently demonstrated that RNA viruses genomes with pervasive RNA structure throughout the genome, which we designate genome-scale ordered RNA structure (GORS), showed a series of distinct biophysical properties (9), such as inaccessibility to external DNA probe hybridization and compact globular morphologies visualized by scanning electron microscopy that contrasted with the unravelled appearance of unstructured RNA genomes (9,10).

RNA structure prediction methods include thermodynamic calculation of minimum folding energies, phylogenetic methods based on stochastic context-free grammar (11) and methods based on combined thermodynamic/co-variance detection (12). These produced concordant predictions for which mammalian positive-stranded RNA viral genome sequences possessed GORS and which were systematically unstructured (9). To our surprise, the occurrence of GORS was highly variable between different virus groups, such as genera or genogroups within the same virus family. For example, in the virus family *Flaviviridae*, hepatitis C virus (HCV), non-primate hepacivirus and GBV-B virus in the genus *Hepacivirus*, and primate, bat and equine pegiviruses in the genus *Pegivirus*, possessed GORS, whereas all members of the *Pestivirus* and *Flavivirus* genera were unstructured (9,13). Variability between genera within families is similarly evident with the *Picornaviridae* (9,14,15) and *Caliciviridae* (16). As replication strategies and RNA packaging are usually well conserved within a family, this variability of RNA structure formation are most unlikely to contribute directly to these parts of virus replication cycle. However, what was immediately apparent was the association between the presence of GORS and the ability of the virus to persist in their natural hosts, pointing towards a different role of RNA structure in modulating the interaction of the virus with the cell and its defences (8).

Investigating the nature of this potential interaction and determining the mechanistic basis for the association between GORS and persistence has been hampered until recently by the absence of a practical model system to reproduce these attributes, either in cell culture or in a whole animal. Additional constraints such as the biological containment level, the availability of tractable reverse genetics system required to generate mutants has also restricted the experimental systems of use. As examples, one or more of these requirements has largely precluded the experimental use of HCV, other hepaciviruses, pegiviruses (both in the family *Flaviviridae*), aphthoviruses and kobuviruses (*Picornaviridae*) and various structured vesiviruses such as canine calicivirus (*Caliciviridae*).

**Figure 1.**
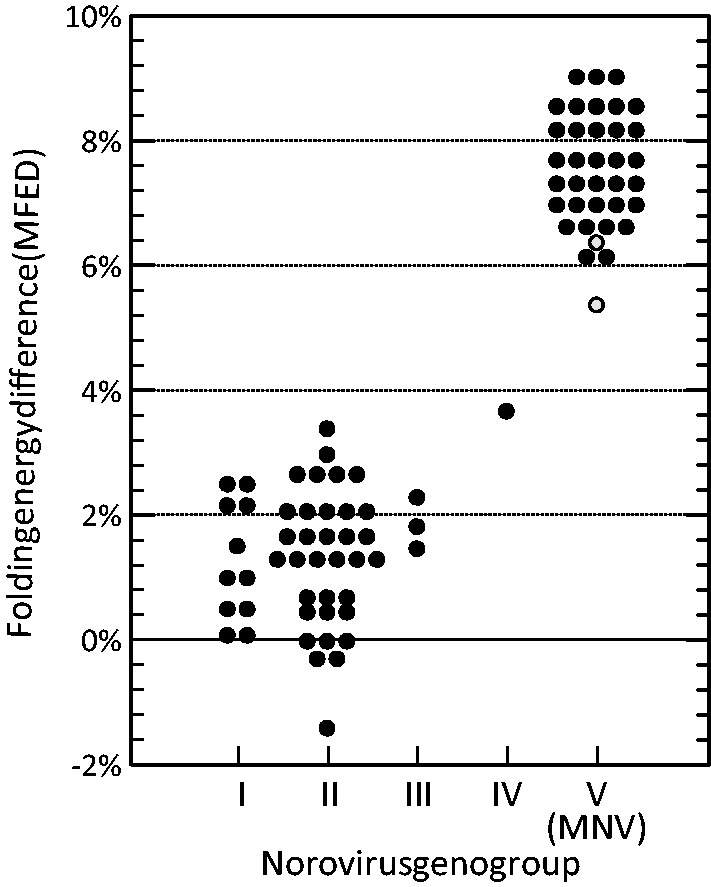
Folding energy differences for members of different genogroups within the *Norovirus* genus. Grey-filled circles represent MFEDs for two MNV-like viruses found in the related rodent species *A.sylvaticus* (25).

The discovery of murine norovirus (MNV) in 2003 (17), which both has the ability to grow in cell culture and to readily infect laboratory mice and the development of efficient reverse genetics strategies to generate infectious virus (18), has recently resolved this experimental block. Although MNV was first found in immunocompromised (STAT-1^−^^/^^−^) mice (17) in which it caused severe and frequently fatal infections, infections in immunocompetent mice are both persistent and non-pathogenic (19). MNV is widely distributed as an unintended contaminating virus in laboratory mice colonies worldwide (20–24) while more recent studies have demonstrated extensive infection among a variety of mouse species in the wild, in which persistence is the norm (25–27). Consistent with this latter attribute, MNV contrasts with other species within the *Norovirus* genus in exhibiting GORS (Figure 1) and thus represents a potential model for functional laboratory investigations. We have recently developed efficient reverse-genetic system to express infectious virus from the pT7:MNV3 clone (28) that can be both cultured in the RAW264.7 macrophage cell line and used to infect immunocompetent mice in which it establishes a persistent non-pathogenic infection (18). MNV3 mutants with large regions of RNA secondary structure removed but which retained coding and other compositional features of the underlying sequences, provided the means to experimentally investigate its effect on replication, immune system interaction and fitness relative to wild-type (WT) virus.

## MATERIALS AND METHODS

### Cell culture and cell lines

MNV3 was propagated in the murine leukaemia macrophage cell line RAW264.7 using Dulbecco modified Eagle medium (DMEM) with 10% foetal calf serum (FCS), penicillin (100 U/ml), streptomycin (100 µg/ml). Baby-hamster kidney cells expressing T7 DNA polymerase (BSRT7 cells) used during reverse genetics recovery of MNV3 from cDNA clones, were obtained from Klaus Conzelmann (Ludwig-Maximilians-University Munich) (29) and cultured in DMEM (plus FCS, penicillin and streptomycin, as aforementioned) containing G418 at a concentration of 1 mg/ml. All cell lines were maintained at 37°C with 10% CO_2_. Bone marrow-derived macrophages (BMDM) were maintained in RPMI 1640 with 25 mM HEPES, 10% FCS, penicillin (100 U/ml), streptomycin (100 µg/ml) and recombinant colony stimulating factor (1 × 10^4^ U/ml; a kind gift from D. Hume, Roslin Institute) at 37°C with 5% CO_2_.

### Antisera

Rabbit polyclonal sera raised against the MNV NS7 polymerase was used as previously described (30).

### Nucleotide sequences used for bioinformatic analysis

Available complete genome sequences of norovirus genogroup (GG) I, III and IV comprised: GGI: SOUCAPPRO, AB042808, AB187514, AF093797, CVXRNA, AB039774, AB081723, FJ515294, JQ388274, JX023285, JQ911594; GGIII: AY126474, AF097917, EU794907; GGIV: JQ613567. Representative GGII and GGV (MNV) sequences showing >2% divergence from each other were selected for analysis and comprised: GGII: AY587983, AY581254, DQ415279, AY502020, AY502023, AY485642, DQ658413, AY741811, AB083781, AF145896, AY032605, X86557, AB220921, AB220923, AB220922, DQ369797, AB039775, AB045603, AB044366, AY237415, AF504671, HCU07611, AY134748, DQ456824, AB039782, AB039781, AY772730, AB083780, DQ366347, AB126320, AB039780, AB084071, AB039777, AB039778; MNV (GGV): DQ223042, DQ223043, DQ223041, AB601769, EF531291, EU854589, EU004670, EF531290, EU004671, EU004676, FJ446720, JN975498, DQ911368, EU004673, FJ446719, EU004680, EU004663, JF320644, AB435514, JF320653, EU004683, EU004674, JN975493, HQ317203, EU004677, JF320652, EU004682, JN975496, EU004660, JN975494, EU004672, EU482057, EU482058, JN975495. Two sequences of MNV-like viruses (JN975491, JN975492) from *Apodemus sylvaticus* (25) were additionally included.

### RNA secondary structure prediction

Mean folding energy differences (MFEDs) were calculated using the program Folding Energy Scan in the sequence editor and analysis program, SSE (31). For calculation of mean MFEDs for whole-genome sequences, mean values for successive 300 base fragments incrementing by 90 bases over the genome were calculated; the first two and penultimate two fragments were excluded from each sequences, as they contain likely specific RNA structures associated with replication and translation functions (16,30,32–34). MFED scans across the genome for MNV used 225 base fragments incrementing by 15 bases to increase resolution of structured regions.

### Suppression of synonymous site variation

Sequence variability at non-coding sites was determined by measurement of mean synonymous pairwise (uncorrected) distances in successive 201 base fragments advancing by 6 bases through the three concatenated open reading frames (ORF), ORFs 1–3 of aligned MNV sequences. This process was automated using the program Sequence Distance in SSE (31).

### Plasmid construction

The previously described full-length MNV3 cDNA pT7:MNV3 clone under the control of a T7 promoter was used for this study (19). Before use, 5′ *Sbf*I (position 11 520) and *Eco*RI (position 11 153) sites were deleted from the pT7 vector using site-directed polymerase chain reaction (PCR) mutagenesis (details available on request). Mutant MNV3 constructs with disrupted RNA secondary structure were engineered by ordering custom-generated DNA sequences (GeneArt, Life Technologies, Paisley, UK). Native sequences were permuted using the CDLR algorithm with MNV3 WT sequence on either side including the 5′ and 3′ respective restriction site to be used for cloning, preceded or followed by several bases of WT sequence. Sequences were provided in standard antibiotic resistant cloning vectors and were cloned into pT7:MNV3 using the unique restriction sites *Sfi*I (genome position 1147) and *Sbf*I (genome position 2468) for fragment 1 (F1) and *Eco*RI (genome position 3124) and *Bgl*II (genome position 4067) for fragment 2 (F2). A double mutant (F1/F2) was similarly generated. All clones were fully sequenced in their coding regions plus T7 promoter before further applications. The permuted F1 and F2 sequences have been submitted to GenBank and have been assigned the accession numbers KC702504 and KC702505.

### Reverse genetics recovery of GORS mutant viruses

GORS mutant viruses were recovered as previously described (18). Briefly, Baby-hamster kidney cells expressing T7 polymerase (BSRT-7) were infected with fowlpox virus expressing T7 polymerase and were subsequently transfected with 1 µg of the MNV3 full-length clones (pT7:MNV3) containing the GORS mutations F1, F2 and F1/F2 (described earlier in the text) using the Lipofectamine 2000 transfection reagent (Invitrogen) according to the manufacturer’s protocol. Then, 24 h post transfection, cells were frozen at −80°C, and the clarified lysates were used to generate passage 1 and 2 stocks by infecting RAW264.7 cells at low multiplicity of infection (m.o.i.) and freezing 48 h post-infection. Viral titres were determined by TCID_50_ titration in RAW264.7 cells. Before use, all viruses were sequenced to ensure they contained the relevant mutations. For all generated viruses, determination of RNA to infectivity ratios was performed in duplicate by extracting RNA from 6.72 × 10^6^ TCID_50_ units and performing subsequent quantitative real time PCR analysis. Extractions were performed in duplicate, and results were analysed by one-way ANOVA with Bonferroni post-tests.

### Growth kinetic analysis

RAW264.7 cells were seeded at 3.2 × 10^5^ cells per well in a 24-well plate and subsequently infected with the WT or GORS mutant viruses F1, F2 and F1/F2 at an m.o.i. of 0.01 TCID_50_ per cell. The assay was performed in triplicate for each virus. At given time points (0, 6, 12, 24, 48, 72 h post-infection), the infected cell monolayers were frozen, and on thawing, the viral titres were determined by TCID_50_. To analyse growth at various temperatures, the assay was set up as above using just WT and F1/F2 mutant virus inocula, and infected cells were incubated at 33, 37 and 39°C, respectively. A high m.o.i. (10 TCID_50_ per cell) virus yield assay was performed 24 h post-infection of BMDMs isolated from the thigh bone of BALB/c mice.

### Analysis of GORS mutant virus stability

RAW264.7 cells were seeded at 3.75 × 10^6^ cells per well of a 6-well dish and were subsequently infected with either F1/F2, WT or both F1/F2 and WT viruses in a 1:1 and 10:1 ratio in favour of the mutant at an m.o.i. of 0.01 TCID_50_ per cell. After 48 h, the resulting ‘pass 1’ cultures were freeze thawed and used to set up the subsequent low m.o.i. (<0.01 TCID_50_ per cell) infections. Virus passage was continued for six cycles after which cells were infected at high m.o.i. and RNA was isolated 12 h post-infection. Reverse transcriptase PCR (RT-PCR) reactions were subsequently used to sequence the region of the genome encompassing GORS F1 (primer details available on request).

### *In vivo* analysis

For the infectious dose assay, groups of six C57BL/six male mice of 4–5 weeks of age (Harlan or Charles River) were initially inoculated by oral gavage with 100 µl of DMEM containing either 10, 100 or 1000 TCID_50_ units of either WT MNV3 or F1/F2 MNV3 stocks. For viral load determination, faeces samples were collected on days 0 (before infection), 3, 7, 14 and 28. In a second inoculation, groups of 12 mice were infected with 1000 TCID_50_ units of WT MNV3 or F1/F2 MNV3 and viral loads in faecal samples determined on days 1, 3 and 5. For the *in vivo* fitness assay, the inoculum consisted of WT MNV3 and F1/F2 MNV3 viruses mixed at a 1:1 ratio (5000 TCID_50_ units of each). Faeces samples were collected on days 0 (before infection), 1, 7 and 28 days for viral population analysis.

For the persistence study, mice were inoculated with 10 000 TCID_50_ units of either WT or F1/F2 MNV3 viruses. For each experiment, six control mice were inoculated with non-infected cell lysates prepared in an identical manner to the virus stocks. Control mice were verified as MNV free at the end of the study, confirming barrier controls were effective. Mice were sacrificed at 9 months post-infection. To collect tissue samples, animals were culled by injection of a lethal overdose of sodium pentobarbital followed by cranial dislocation. Tissue samples taken include spleen and mesenteric lymph node, caecum and colon. Faeces samples were stored at 4°C. Tissue samples were stored in RNA later (Ambion) and stored at −80°C, before the RNA was extracted, as per the manufacturer’s instructions.

### RNA extraction

Faecal pellets were suspended in phosphate buffered saline (50 mg/ml) before homogenization, and 100 µl of the supernatant obtained after centrifugation at 4000g for 20 min at 4°C was used for RNA purification. For tissue samples, RNA was extracted from ∼20 mg of each tissue following the indications provided with the GenElute Mammalian Total RNA Miniprep kit (Sigma-Aldrich).

### Quantitative RT-PCR quantification of viral load

To detect the number of MNV3 RNA molecules in faecal or tissue samples, reverse transcription–quantitative PCR was performed. Reverse transcription was performed using M-MLV reverse transcriptase (Promega) and primer 5380R (Supplementary Table S1). MNV cDNA was then quantified by quantitative real time PCR with primers spanning residues 5028S and 5177R, and a TaqMan FAM-TAMRA-labelled probe complementary to residues 5077–5062 (Supplementary Table S1). Quantitative PCR determinations were carried out with Precision 2× qPCR MasterMix (Primerdesign) in a ViiA7 Real-Time PCR system apparatus (Applied Biosystems). In all the experiments, a standard curve for MNV RNA with a known number of molecules was carried out in parallel. The limit of detection was determined either by the lowest dilution of control standard.

### qRT-PCR for cellular genes TNF, ISG15, ISG54 and ISG56

RAW264.7 cells were infected with F1/F2 or WT MNV3 at an m.o.i. of 1 TCID_50_ per cell. As a negative control, cells were treated with a cellular lysate harvested from uninfected RAW264.7 cells. The assay was performed in triplicate. At specified time points post-infection (8 or 24 h post-infection), total RNA was extracted from uninfected or infected RAWs using an RNeasy kit (Qiagen) and was subjected to DNAse 1 (Promega) treatment before being reverse transcribed using M-MLV reverse transcriptase and random hexamer primers (Promega). Semi-quantitative PCR was performed using a Rotorgene Q cycler (Qiagen) using SensiFAST™ SYBR Hi-ROX One-Step master mix (Bioline) and primers (0.8 Μm) specific to TNF, ISG15 (35), ISG54 and ISG56 (36). Each gene was normalized to HPRT1 (37) or 18 s rRNA (primer details in Supplementary Table S1), and the relative expression was calculated using 2^−^^ΔΔCt^ (38). Data were plotted as fold change per virus over the negative lysate control.

### Interferon reduction assay

RAW246.7 cells were pretreated with 250 U interferon (IFN)-α (PBL Inteferon source) or 10 U IFN-γ (Life technologies) for 24 h before infection with an m.o.i. of 1 with F1/F2 or WT MNV3. Interferon treatment was maintained for the duration of the experiment (8 or 24 h post-infection) at the specific concentration stated previously. In parallel, untreated RAW246.7 cells were infected with an m.o.i. of 1 with F1/F2 or WT MNV3. The assay was performed in triplicate. RNA was extracted from treated and untreated infected RAW246.7 cells, using the QiaAmp viral RNA mini Kit (Qiagen). The viral load of each sample was amplified from cDNA synthesised using M-MLV reverse transcriptase and random hexamer primers (Promega), using SensiFAST™ SYBR Hi-ROX One-Step master mix (Bioline) and primers (0.8 μM) 5275S/5452R. The RNA levels of untreated and treated RAW246.7 cells were normalized using 18 s rRNA before data analysis.

### Sequencing of the F1 and F2 regions

Total RNA from each faecal sample was subjected to DNAseI (NEB) digestion according to the manufacturer’s protocol and used for reverse transcription using the SSIII RT enzyme (Life Technologies) and primer 7350R (Supplementary Table S1). Before PCR amplification, the sample was treated with RNAseH (NEB) according to the manufacturer’s protocol. The F1 region was then amplified with two sets of nested PCR primers complementary to the flanking regions of GORS F1. The first and second round amplification used sense and antisense primers 1081S/2650R followed by 1121S/2500R (Supplementary Table S1). The F2 region was amplified primers 2902S/4155R and 2961S/4128R. The F3 region was amplified primers 2342F/3099R and 2412F/3035R. Amplification was performed using KOD hot start polymerase (Novagen) according to the manufacturer’s protocol. Sequencing was carried out by GATC Biotech sequencing service.

### Restriction enzyme cleavage assay

The relative proportions of WT and mutant sequences in competition assays were determined by unselective nested PCR amplification of MNV sequences by PCR using primers 1081F/2650RR followed by 1121F/1522R (Region 1) and 2902F/4155R and 3678F/4128R (Region 2). Amplicons were column purified and cleaved with the F1-specific restriction endonuclease (RE), *Xho*I to generate fragments of 279 and 163 bps (Region 1) and by BspMI to generate fragments of 400 and 50 bp (Region 2). Cleaved DNA is readily distinguishable in size from uncut (WT) DNA sequences on standard agarose gel electrophoresis. Relative proportions of cleaved and uncleaved DNA were determined by densitometry. The relationship between band intensity and composition was calibrated by amplifying WT and mutant target sequences pre-mixed in different ratios. In both regions, there was a non-linear relationship between band intensity of cleaved and uncleaved DNA as a result of the formation of uncleavable heteroduplexes of DNA towards the end of the amplification reaction. Although introducing some likely reduction in the precision of the assay, the apparent over-representation of uncut WT sequences could be accommodated by construction of a (non-linear) standard curve with which to convert densitometry reading for unknown samples into WT/mutant ratios.

### Mutant specific PCR

For the detection of F1 and F1/F2 mutants in the *in vivo* fitness assay, a round one PCR product was generating using 2141S and 2650R (Supplementary Table S1) using the cDNA generated by reverse transcription as described earlier in the text. This was then amplified in a second round of PCR using the forward primer 2206F and the F1-specific reverse primer 2401F1R (Supplementary Table S1). For the detection of the F2 mutant in the *in vivo* fitness assay, the round one PCR product between positions 2902 and 4155 was amplified using the forward primer 3678F and the F2 specific reverse primer 3846F2R. GoTaq Flexi DNA polymerase (Promega) was used for this amplification, and the reactions were subject to 15 amplification cycles.

### Ethics statement

All of the animals used in this study were maintained in an animal facility at Imperial College London St Mary’s Campus (PCD 70/2727) after ethical review by the Imperial College Ethical Review Panel and subsequent approval of the British Home Office (PPL 70/6838). All animal procedures and care in the UK conformed to the United Kingdom Home Office Guidelines under The Animals (Scientific Procedures) Act 1986.

## RESULTS

### MNV as a model structured virus

**Figure 2.**
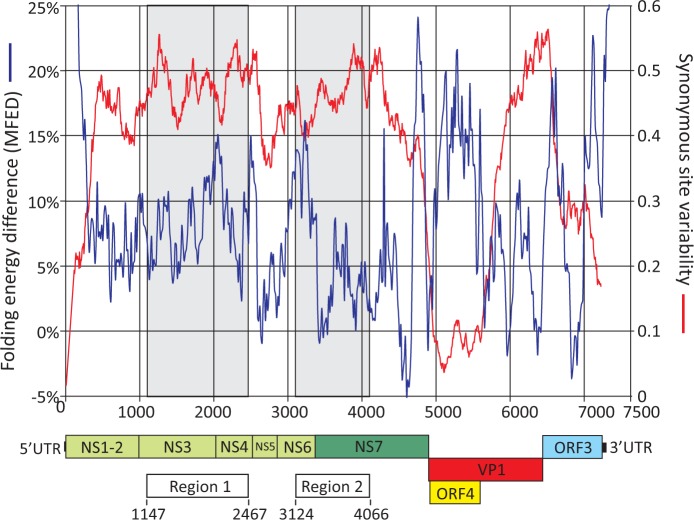
Folding energies (left-hand *y*-axis) and synonymous sequence variability (right-hand *y*-axis) of aligned MNV sequences across the viral genome. Positions of inserts of destabilized sequences (Regions 1 and 2) are shown superimposed on a diagram of the coding sequences in the MNV genome drawn to scale.

MNV shows a number of biological differences from other genogroups of noroviruses, including an absence or limited enteric disease manifestations and an ability to establish persistent infections in immunocompetent mice. To determine whether MNV possessed GORS, comparisons were made between the folding energy of native sequences of different groups of caliciviruses with those of replicate sequences permuted in sequence order (using an algorithm that preserves the mono- and dinucleotide composition of the native sequence). Differences in folding energy (MFEDs) represent the biologically relevant sequence order-dependent component of the sequence (39,40). Consistent with the previously described association between persistence and possession of large-scale RNA structure (9), mean folding energies of MNV genome sequences were higher than those of other norovirus genogroups (Figure 1). High MFED values were observed across the genome of MNV (Figure 2), a distribution that contrasted with its restriction to the genome ends and the ORF1/ORF2 junction of other norovirus genogroups (16).

We have previously reported the development of a reverse genetic system based on a pT7:MNV3 cDNA (GenBank accession number JQ658375) (19). MNV recovered following transfection of this clone was replication competent in cell culture and showed an ability to infect and persist in immunocompetent mice for at least 56 days (19). We used this model to investigate the effect of RNA secondary structure on the ability of MNV to replicate and persist *in vivo.* Two genome regions in ORF1 (positions 1152–2461 and 3129–3965, respectively, designated Region 1 and Region 2; Figure 2) were selected for mutagenesis based on several criteria. Both targeted regions showed evidence for RNA secondary structure as measured by MFED calculations (Table 1). Region 1 showed mean MFED levels of 9.1%, a level typically found in RNA viruses with GORS (9) and a *Z*-score for the whole fragment of −4.2, over four standard deviations away from the mean of the distribution of permuted sequence controls. Region 2 showed a lesser degree of RNA folding (MFED 3.9%, *Z*-score: −1.7). In addition, both regions avoided demonstrated or suspected replication, translation or transcription elements as well as the overlapping ORF at the start of the capsid gene, ORF4 (41). They showed high degrees of variability at synonymous sites (red line in Figure 1) that contrasted with suppression in the 5′ and 3′ ends of the genome, the proposed subgenomic transcriptional promoter and the region in ORF2 coding for virulence factor (VF)-1 in a different reading frame (41).

**Table 1.** Folding energies of WT and mutated region 1 and region 2 sequences

Region	Positions	Sequence	MFE^a^	Control^a^^,^^b^	MFED^c^	*Z*-score
1	1147–2467	WT	−97.0	−90.5	9.10%	−4.2
		F1	−88.5	−91.6	−0.9%	−0.15
2	3124–4066	WT	−104.0	−100.7	3.90%	−1.7
		F2	−96.2	−97.7	−2.6%	−0.01

^a^Minimum free energy on folding (kcal/mol).

^b^Folding energy of sequence permuted by the NDR scrambling algorithm.

^c^MFE difference between non-permuted and permuted sequences.

Sequences between these positions were permuted by the program Sequence Scramble in SSE, using the CDLR algorithm. This permutes nucleotide sequence order while retaining coding order and base composition (including dinucleotide frequencies) of the native sequence. The CDLR algorithm generated sequences with substantially reduced folding energies compared with WT sequences (MFED values of −0.9 and −2.7% for Regions 1 and 2, both effectively no different from folding energies of randomly ordered RNA sequences; Table 1). These were then synthesised and individually cloned into the pT7:MNV3 infectious MNV clone using naturally occurring restriction sites (Figure 2) to generate F1 and F2 mutants. A double mutant (F1/F2) containing both GORS destabilized fragments was also generated.

### Influence of RNA structure on MNV3 replication in cell culture

Using the MNV3 reverse genetics system (19), both the single and double mutant viruses were recovered alongside WT MNV3. Recovery of wild-type and GORS mutant viruses was performed using fowl pox-mediated expression of T7 RNA polymerase to drive the synthesis of MNV3 RNA in cells transfected with full-length cDNA constructs as described previously for MNV1 (30). The initial yields of GORS mutant viruses were comparable with that derived from WT cDNA, although it was on average 10-fold lower than observed for MNV-1 (∼1−5 × 10^3^ TCID_50_ per 35 mm dish, data not shown), western blot analysis of protein lysates of transfected cells for the viral polymerase NS7 revealed equal expression levels between the WT and mutant viruses (Supplementary Figure S1A).

**Figure 3.**
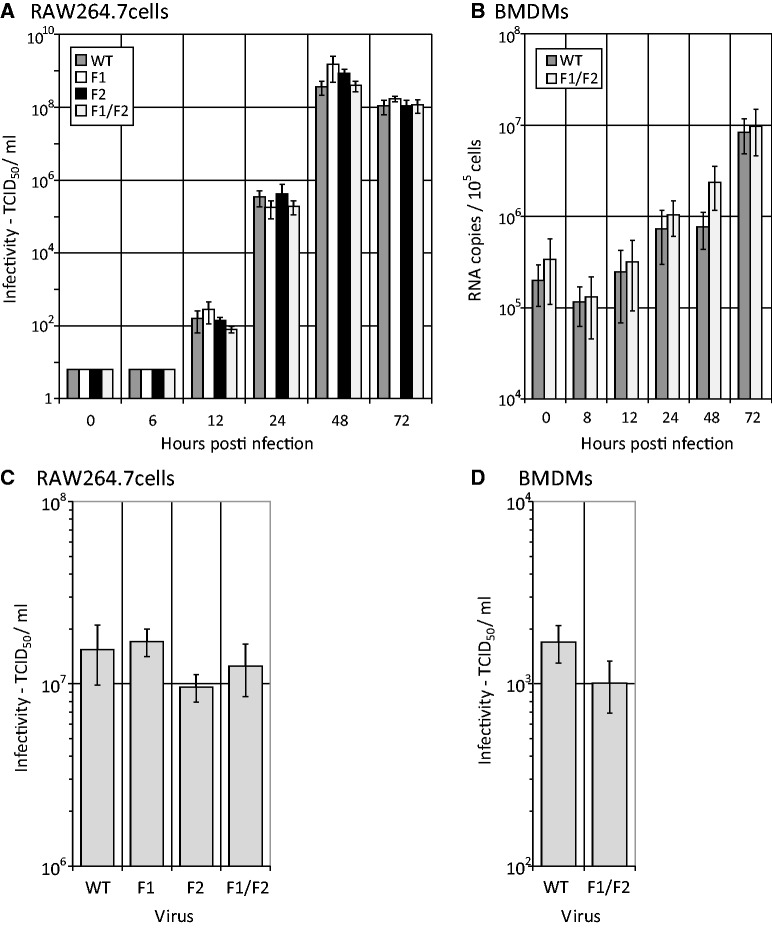
(**A**) Multi-step growth curves of WT and F1, F2 and F1/F2 RNA structure-disrupted mutants in RAW264.7 cells infected at an m.o.i. of 0.01 TCID_50_s/cell. Released virus was quantified by infectivity assays of cell culture supernatant (TCID_50_s/ml). (**B**) Growth curves of WT and F1/F2 mutants in BMDMs infected at an m.o.i. of 1. Released virus was quantified by qPCR (RNA copies/ml). (**C** and **D**) Infectivity titres of cell lysates 24 h after infection of RAW264.7 cells and primary BALB/c BMDMs at high m.o.i. (10 TCID_50_./cell).

To determine RNA copy to infectivity ratios, viral RNA was extracted from viral lysates of known infectious titres for each of the four viruses and quantified by real-time PCR. Ratios were similar between the WT and all three mutant viruses, ranging from 28 (WT) to 78 (F2) RNA copies/TCID_50_ (Supplementary Figure S1B). Growth kinetics of the GORS mutant viruses were compared with WT cDNA-derived MNV3 and demonstrated no differences in either low m.o.i. or high m.o.i. infections in RAW264.7 cells (Figure 3A and C). As no differences were observed between the single (F1 or F2) and double mutant (F1/F2) and WT MNV3, subsequent *in vitro* studies were performed directly with the double mutant F1/F2. Although no differences in replication rate was observed at 37°C, the existence of differences in temperature sensitivity was investigated by culture of WT and F1/F2 MNV3 viruses at 33 , 37 and 39°C. For both viruses, replication kinetics varied by temperature, and culture at both 33 and 39°C showed reduced viral yields compared with 37°C. However, no significant difference was observed between WT and mutant viruses at any temperature (Supplementary Figure S2). To investigate whether differences in replication ability might exist in primary cells more representative of cell types infected *in vivo*, adherent monolayers of pre-differentiated BMDMs were infected with WT MNV3 and F1/F2 MNV3 at an m.o.i. of 10 TCID_50_/cell, and RNA was quantified in the supernatant over the following 72 h (Figure 3B and D). Both viruses replicated in BMDMs with similar kinetics, determined both by quantitation of genomic RNA (Figure 3B) and infectivity measurements of supernatant collected at 24 h (Figure 3D).

**Figure 4.**
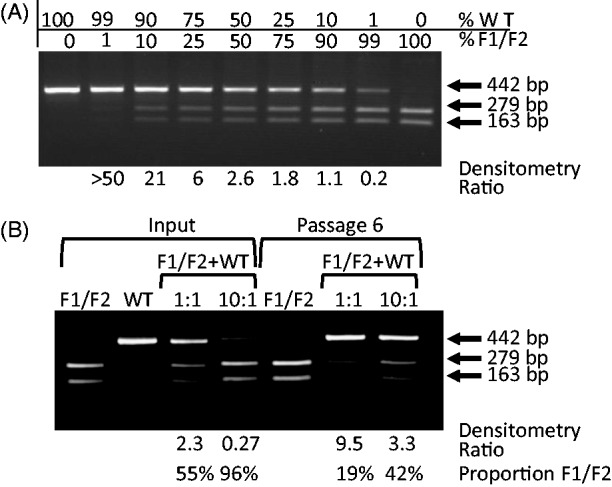
Characterization of viral populations generated in the *in vitro* competition assay. (**A**) F1/F2 and WT PCR products were mixed at different ratios and were cleaved with F1-specific *Xho*I to demonstrate assay sensitivity. (**B**) PCR products generated from a RT-PCR of inoculum virus preparations (single and mixed at in a 1:1 and 10:1 ratio of F1/F2 over WT) were digested with *Xho*I and compared with the digested RT-PCR products from viral lysates prepared from infected cells after six passages in RAW264.7 cells.

As a more sensitive indicator of relative fitness of WT and structure-disrupted viruses, a competition assay was performed in which the F1/F2 mutant virus was serially passaged in RAW264.7 mouse macrophages in the presence of WT virus. Inocula comprised WT and F1/F2 MNV3 mixed in 1:1 and 10:1 infectivity ratios (as determined by titration in RAW264.7 cells) in favour of the mutant. After six passages, progeny virus was genetically characterized by amplification of the F1 region and amplicons cut with a restriction enzyme (*Xho*I) specific for the F1/F2 mutant. To determine the relationship between input ratios of WT and F1 sequences and relative band intensities of cleaved and uncleaved DNA, known amounts of F1/F2 or WT-specific PCR product were pre-mixed at different ratios, amplified by PCR and cut with *Xho*I (Figure 4A). The degree of cleavage observed in the RE assay varied according to input ratios of the F1/F2 and WT templates, although its relationship with band intensity assessed by densitometry was non-linear. This was likely a result of the formation of uncleavable heteroduplexes of DNA towards the end of the amplification reaction (a much more linear relationship was observed when WT and F1 DNA sequences were mixed in different ratios and cleaved without amplification; data not shown).

This technique was then used to analyse MNV extracted from cell culture passaged virus in the competition assay (Figure 4B). As controls, complete cleavage (F1/F2) or no cleavage (WT) was observed after six passages as mono-infections, whereas RNA extracted from inocula of F1F2 and WT virus mixed in 1:1 and 10:1 ratios showed a predicted 46 and 95% representation of F1 sequences. Progeny virus was obtained at passage 6 from both competition assays. As each passage was of 48 h duration during which a cytopathic effect developed, six passages therefore represent a minimum period of 192 h (8 days) if it is assumed that MNV was actively replicating for at least two thirds of the passage time. WT and mutant viruses remained detectable at passage 6 (Figure 4) with relatively limited changes in population representation compared with the inoculum; based on densitometry and calibration against the standards, F1/F2 comprised approximately one-fifth of the combined population compared with a measured 45% at the start of the experiment. A greater proportionate reduction in F1/F2 representation in the competition assay using a 10:1 F1/F2/WT input ratio was observed, although this competition assay was more prone to stochastic effects in the RE assay and on culturing.

To investigate potential differences between WT and structure-disrupted mutants of MNV in their interaction with host cell defences, the induction of IFN-β and various IFN-stimulated genes (ISGs) in RAW264.7 cells infected with WT and F1/F2 viruses was compared. Unlike previous studies of the pathogenic MNV1 isolate (41), infection at an m.o.i. of 1 TCID_50_/cell by both WT and mutant F1/F2 MNV3 did not stimulate a detectable IFN-β response as measured by quantitative PCR for mRNA transcripts at either 8 or 24 h time points in either RAW264.7 cells or primary BMDMs (data not shown). Furthermore, low levels of ISG15, 54, 56 and TNF-α were detected 8 h post-infection with levels only 1.5× to 2× greater than mock-infected controls in either cell type (Supplementary Figure S3A and data not shown).

The replication of WT and F1/F2 variants of MNV in RAW264.7 cells pre-treated for 24 h with and cultured in the presence of 250 U of IFN-α or 10 U IFN-γ was compared with investigate the potential protection of GORS in cells induced into an antiviral state (Supplementary Figure S3B). Both IFN-α and IFN-γ reduced replication of WT MNV to low levels compared with untreated cells, but the F1/F2 mutant virus showed equivalent susceptibility to both classes of IFN (*P* > 0.05).

### Phenotypic characterization of MNV GORS mutants *in vivo*

Although no differences in replicative fitness was observed in cell culture, previous characterization of MNV mutants that were non-attenuated in cell culture demonstrated significant replicative defects in a whole animal model (34,41). To investigate whether the effect of RNA structure disruption was similarly only manifested *in vivo*, we inoculated immune competent C57BL/6 mice with three challenge doses (10, 100 or 1000 TCID_50_) of WT, F1, F2 and F1/F2 mutant viruses and assayed faecal samples for MNV RNA initially for a month post-infection (inoculation A) and in a repeat inoculation (B) at time points (days 1, 3 and 5) to quantify replication in the gastrointestinal tract.

**Figure 5.**
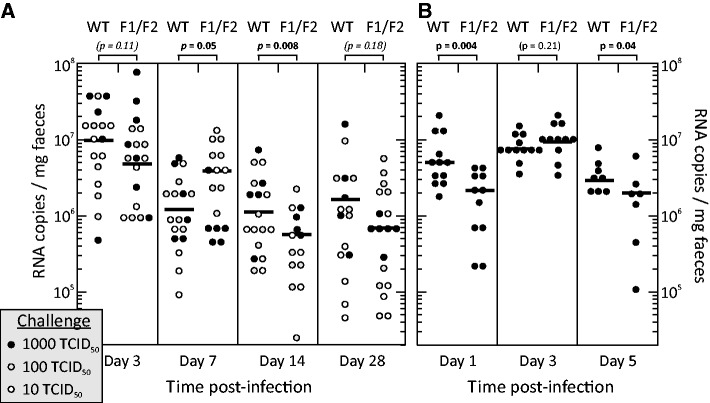
(**A**) Groups of six mice were infected with 10, 100 or 1000 TCID_50_s of either WT MNV3 or the F1/F2 double mutant. (**B**) Repeat inoculation of larger groups of mice with 1000 TCID_50_s of WT F1/F2 double mutant MNV. Viral loads (expressed as RNA copies/mg faeces) were assayed on days 3, 7, 14 and 28 post-infection (A) or days 1, 3 and 5 (B). Log mean values are shown as horizontal bars. Viral load distributions between WT and F1/F2 infected mice were compared using the Kruskall–Wallis non-parametic test; significant differences (*P* ≤ 0.05) are shown in bold type.

As previously observed (19), infections were non-pathogenic, and infected mice showed similar identical weight gain to uninfected controls (data for inoculation A shown in Supplementary Figure S4). Viral shedding was detected in faeces from the earliest collection point (day 1) through to day 28 in mice infected with both WT and GORS-disrupted mutants (WT and F1/F2 shown in Figure 5; F1 and F2 single region mutants; data not shown) but on no occasion in uninfected controls (data not shown). Viral loads were highest at day 3 with ∼10^7^ RNA copies/mg faeces that were typically with higher loads in mice infected with the highest inoculum dose (1000 TCID_50_; black filled circles). Viral loads subsequently declined to means values of ∼10^4^ RNA copies/mg at day 28 in both WT and F1/F2 infected mice.

Although both WT and GORS-disrupted mutants were fully replication competent in mice, some differences in the kinetics of primary infection were observed. Most strikingly, mice infected with the F1/F2 mutant showed a 4-fold lower mean viral load than those infected with WT on day 1 (*P* = 0.004). Beyond this time point, viral loads were comparable between groups at day 3 in both sets A and B, day 5 (inoculation B) and days 7–28 (inoculation A), although with some variability between dosage groups. For example, mice infected with 10 or 100 TCID_50_ of F1/F2 showed higher viral loads than WT at day 7, but F1/F2 viral loads were lower subsequently (approximately on half of mean viral loads observed in WT-infected mice at days 14 (*P* = 0.008) and day 28 (not significant).

Subsequently, persistent virus excretion was detected throughout the 9-month infection period of the experiment in all WT and F1/F2-infected mice. These low levels precluded viral load comparisons between WT and F1/F2-infected mice in this sample type. At the study termination, tissues were harvested, and viral loads in caecum, colon, mesenteric lymph nodes and spleen were determined by quantitative real-time PCR (Supplementary Figure S5). Viral loads ranging from 10 to 10^4^ RNA copies/mg tissue were detected with no significant differences detected between WT and F1/F2-infected mice in any tissue.

### Genetic analysis of mouse passaged WT and F1/F2 mutant viruses

**Figure 6.**
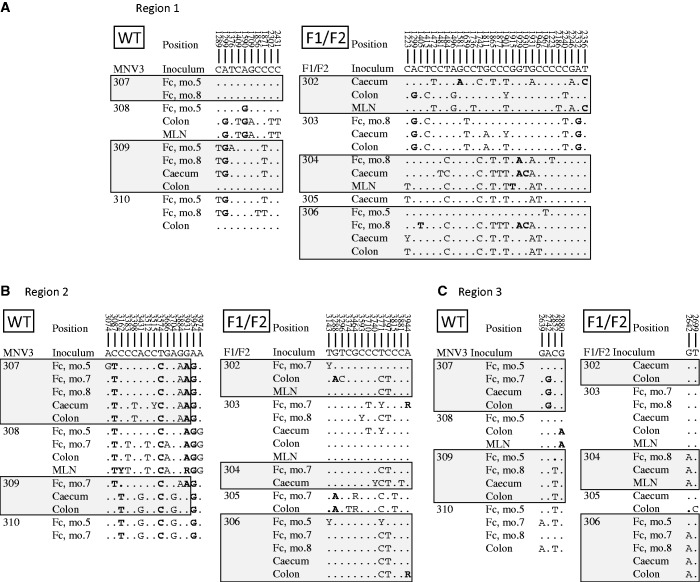
Polymorphic sites identified among viruses recovered from tissues at 9 months and from faecal samples collected at 5 and 8 months post-inoculation with mutant and WT MNV. Regions 1 and 2 are sequences from the F1 and F2 insert regions (positions 1147–2467 and 3124–4066, respectively), whereas Region 3 shows sequences obtained from an originally non-mutated control region in the F1/F2 mutant between positions 2468–3123. Numbers at the top indicate positions of bases in the pT7:MNV3 sequence; sites in bold represent non-synonymous substitutions. Individual mice identified in left hand column; mo., month; Fc, faeces; MLN, mesenteric lymph node.

**Figure 7.**
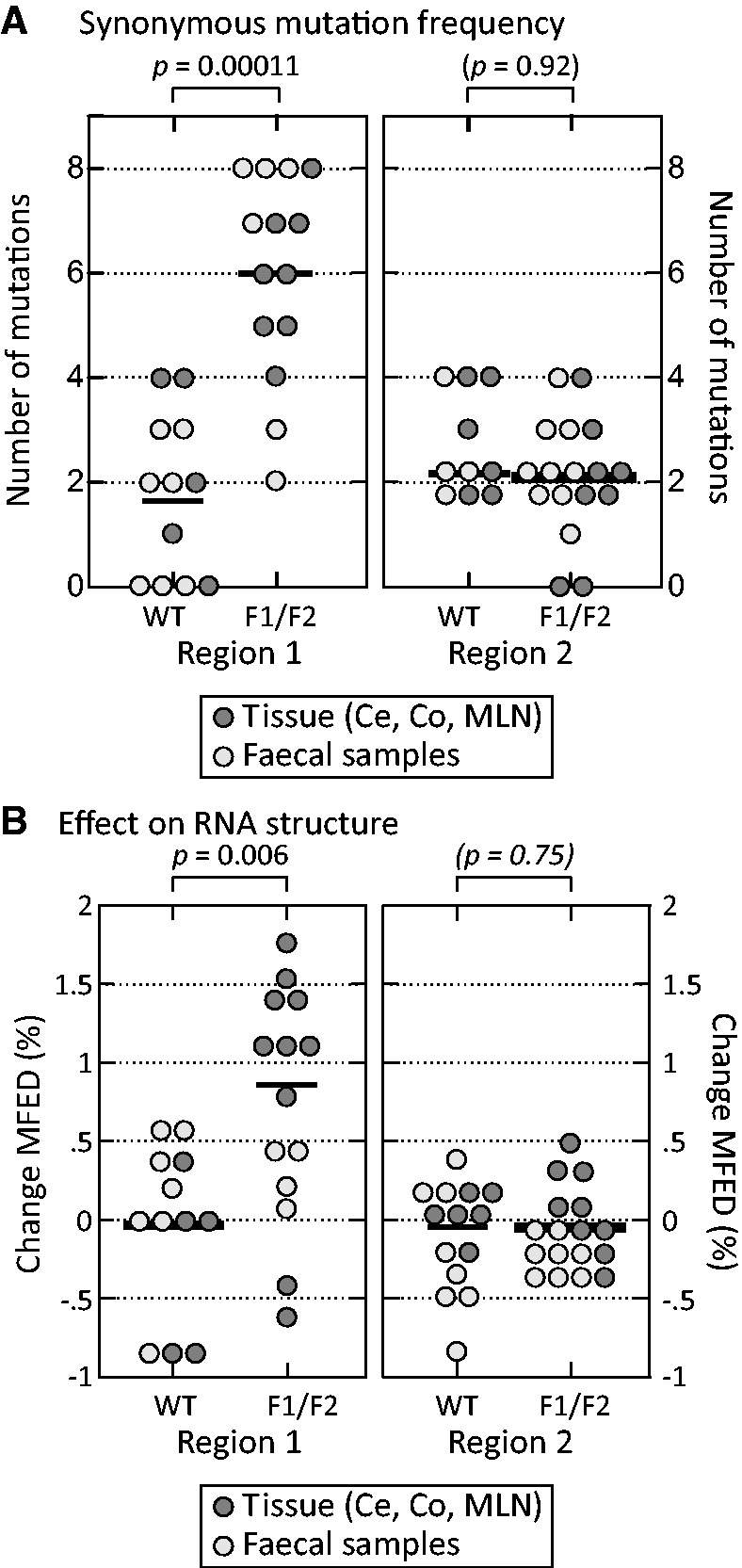
(**A**) Number of sequence changes and (**B**) differences in folding energies of regions 1 and 2 sequences between inoculum viruses and variants recovered between 5 and 9 months post-infection from faecal samples and tissues (caecum, colon, mesenteric lymph node). Mean values shown as horizontal bars; *P-*values shown above graph (Kruskall–Wallis non-parametric test).

To examine the possibility of mutations occurring in the F1/F2 viruses over time that potentially contributed to their ability to persist, variants detected in faecal samples between 5 and 8 months and from tissues (caecum, colon, spleen) were sequenced and compared with inoculum sequences (Figure 5). In Region 1, a much larger number of mutations were observed in mice infected with the F1/F2 mutant than the WT MNV3 control. Most mutations occurred at synonymous sites scattered throughout the region (Figure 6A), with a mean frequency of 6.0 in region 1 of F1/F2 infected mice compared with only 1.75 in WT MNV3-infected mice (*P* < 0.001; Figure 7A). Much lower mutation frequencies were observed in F1/F2 (and WT)-infected mice in genome regions that had not been mutated. Sequencing the entire region between the F1 and F2 inserts (designated Region 3 in Figure 6) revealed only two polymorphic sites in F1/F2-infected mice, actually lower than the four variable sites in WT-infected mice.

Several synonymous mutations occurred independently in different mice providing observational evidence for selected sequence change independently of coding in this region. Mice infected with WT MNV3 showed a higher proportion of non-synonymous mutations in region 2 (Figure 6B), with complete or nearly complete replacement of 3–4 amino acids in all infected mice. Frequencies of synonymous substitutions were similar between WT and F1/F2-infected mice (mean frequencies of 2.2 and 2.1 per sequence, respectively; Figure 7A).

Of the 43 polymorphic sites in sequences obtained from the F1/F2 infected mice, four represented reversions back to the original base in the pT7:MNV3 clone sequence from which the mutated sequences were derived (positions 1415, 1472, 1742 and 3881). A further two sites (at positions 3815 and 3296) were variable between pT7:MNV3 and other MNV sequences (e.g. MNV1), but neither sequences change was a reversion to the pT7:MNV3 sequence.

Recovered sequences were analysed for RNA secondary structure through calculation of MFED values and comparing these with folding energies of inoculum sequences (Figure 7B). In Region 1, all but two sequences recovered from viruses infected with the F1/F2 mutant showed increases in folding energies over the 9-month period of infection (mean change: +0.73%), whereas sequences recovered from mice infected with WT virus showed no overall change (mean: 0.00%), a difference in distribution that was statistically significant (*P* = 0.006). Consistent with the lower mutation rates, no changes in folding energies were identified among sequences in Region 2 from either WT- or F1/F2-infected mice (Figure 7B). Precise RNA structure predictions were precluded by the occurrence of different mutations in different sequences. As there would likely be no conservation in folding between sequences, such predictions would be speculative in the absence of covariance data or physical structure mapping.

### Fitness comparison of WT and F1/F2 mutant viruses *in vivo*

**Figure 8.**
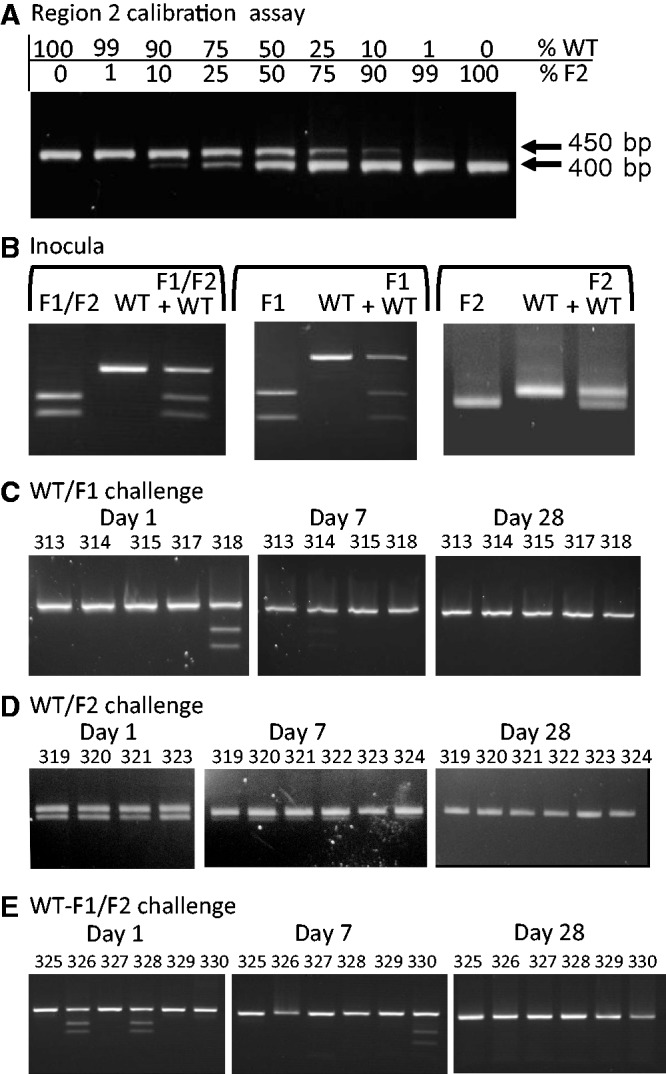
Competition assays between WT and F1, F2 or F1/F2 mutants in mice using REs to identifiy viral population frequences. (**A**) Calibration of RE assay for the F2 region (see Figure 4A for equivalent assay of the Region 1 RE assay); (**B**) Inocula used to infected mice in the three competition assays comprising 50%/50% TCID_50_ ratios of WT and mutant viruses; (**C–E**) Populations of mutants and WT viruses at Days 1, 7 and 28 days post-infection; individual mouse number codes are shown above lanes.

Although cell culture experiments demonstrated that the GORS F1/F2 mutant exhibited similar fitness to WT virus in competition assays, lower viral loads were observed from day 14 after resolution of acute infection in mice infected with F1/F2 variants (Figure 5). As a more sensitive test of relative fitness of WT and GORS-disrupted mutants *in vivo*, groups of six C57BL/6 mice were inoculated with 5 × 10^3^ TCID_50_ of WT MNV3 together with 5 × 10^3^ TCID_50_ of F1, F2 or F1/F2 MNV3 mutants. Viral populations within samples taken at 1, 3, 7, 14 and 28 days post-infection were determined by F1- and F2-specific cleavage assays, direct Sanger sequencing of insert regions and by F1- and F2-specific PCR. As before, an F2-specific RE cleavage assay was developed using *Bsp*MI to cleave amplicons from the F2 region to generate a 400 bp band that was electrophoretically distinguishable from the uncut WT sequences (Figure 8A). F1 and F2 mutant-specific PCRs (MSPs) were developed to detect minor populations through selective amplification using primers specifically targeted to variable regions within Regions 1 and 2. Through assay of pre-mixed sequences, frequencies of mutant sequences in ratios to WT as low as 1:10 000 and 1:1000 could be detected (Supplementary Figure S6).

Using RE and MSP assays, F1, F2 and F1/F2 mutants were replaced by WT virus with varying kinetics. For F1 and F1/F2-specific mutants, mutant populations were infrequently detected by RE analysis using *Xho*I from time points as early as Day 1 (Figure 8C and E and Table 2) and became undetectable by the more sensitive MSP assay beyond day 7 (Table 2). Consistent with these observations, consensus sequencing of polymorphic sites in the F1 region amplified from the inoculum mixture and from mouse samples collected on days 1 and 7 demonstrate the loss of mixed virus populations by day 7 (Supplementary Figure S7; one representative sequence shown). The F2 mutant was similarly out competed by WT virus, although with slower kinetics; e.g equal proportions of WT and mutant viruses were detected in most mice on days 1 and 3 and remained visible in the RE assay on day 7 (Figure 8B). F2 mutants were detectable until at least day 7 by MSP assay (Table 2). By day 28, all mice showed complete replacement of structure disrupted mutants by WT MNV3 in both RE and MSP assays.

**Table 2.** Detection of WT and mutant MNV variants in mouse competition experiment

Variant	Method	Detection frequency				
		Day 1	Day 3	Day 7	Day 14	Day 28
F1	RE	1/5	0/4	0/4	N/A	0/5
	MSP	5/5	4/4	4/4	N/A	1/4
F2	RE	4/4	2/4	5/6	N/A	0/6
	MSP	4/4	2/4	5/6	N/A	0/6
F1/F2	RE	2/6	1/6	1/6	0/6	0/6
	MSP	6/6	6/6	6/6	3/6	0/6

## DISCUSSION

This study investigated the functional role of large-scale RNA structure (GORS) in the genome of a model virus, MNV both *in vitro* and *in vivo.* The rapid replacement of structure disrupted mutants (F1 and F2) by WT virus *in vivo*, despite their equivalent replication kinetics and similar fitness in cell culture competition assays in cell culture demonstrated a strong selective advantage for native viruses that possess high levels of structure in the genome. These observations are consistent with the results of previous bioinformatic and biophysical analyses that demonstrated its variable occurrence in different virus species and genera and a strong association with persistence in the virus’s natural host (8).

### MNV replication in cell culture and *in vivo*

A reductive approach was used to investigate the effect of large-scale RNA secondary structure on MNV replication and fitness. Two regions of the genome were identified that showed evidence for GORS (MFEDs of 9.1 and 3.9% in Regions 1 and 2). Regions 1 and 2 lack known replication or translation elements, transcriptional promoters or documented overlapping reading frames such as ORF4 (positions 5069–5707) encoding VF-1 (16,30,32–34,41). We additionally sought to avoid regions of the genome showing suppression of variability at synonymous sites that may reflect additional non-coding constraints (16) (Figure 2). The combined length of Regions 1 and 2 (2264 bases) represented approximately one-third (31%) of the genome; given the constraints under which regions can be selected for mutation, this is close to the maximum that can be disrupted without destroying known replication elements or the expression of accessory proteins such as VF-1.

RNA structure within regions 1 and 2 was destroyed by permuting the order of bases using an algorithm [CDLR; (31)] that preserves both coding and dinucleotide composition of the native sequences. The latter was necessary following recent reports that the replication of poliovirus was substantially attenuated in mutants with artefactually increased CpG and UpA dinucleotide frequencies [both are under-represented in poliovirus genomes and in most RNA viral genome sequences (42), including MNV (63% of expected values based on G+C content)]. Using several different assays of *in vitro* replicative ability, we were unable to detect any substantial replication defect in RNA-structure disrupted MNV variants (F1, F2 or F1/F2) in RAW264.7 cells. Their comparable fitness *in vitro* provides reassurance that the process of sequence scrambling did not disrupt uncharacterized RNA structure or sequence-based elements critical for virus replication. The impaired fitness of GORS mutants observed in mice therefore cannot be directly attributed to differences in their ability to infect, translate or replicate within a cell or to generate infectious virus progeny.

In common with other caliciviruses, MNV genomes are covalently linked at the 5′ end to VPg that is required for translation initiation (30,43). It has been suggested that this may have the secondary effect of preventing recognition by RIG-I with detection of replicating virus mediated through recognition of long dsRNA sequences by MDA5 (44). Phosphorylation of the latter is then signalled through IRF3 to the nucleus where expression of IFN-β, IFN-λ and several ISGs (−15, −54, −56, −60) and other antiviral proteins (e.g*.* 2′-5′-oligoadenylate synthetase-like protein, zinc finger antiviral protein) is induced (45). IFN-β induction by MNV3 in RAW264.7 cells was minimal, although we cannot exclude that interactions with as yet unrecognized early innate immune response components may underlie the impaired replication of F1/F2 mutants early on primary infection *in vivo* (Figure 5B).

### MNV persistence and adaptive changes

Consistent with previous studies (19) and underlying the choice of MNV3 as the model virus for performing RNA structure investigations, both WT and mutant clones were capable of long-term persistence in immunocompetent animals. Both WT and F1/F2 viruses established non- or minimally pathogenic infections with prolonged faecal shedding of virus and ongoing low level replication in tissues collected 9 months post-inoculation. High frequencies of MNV persistence have been observed in a range of rodent species in the wild (25–27), and the MNV3/*Mus musculus* model may therefore, at least in essential aspects, reflect its pathogenesis and natural life cycle.

In using the newly developed MNV3/immunocompetent mouse model to study persistent RNA virus infections, we acknowledge there is currently only incomplete information on the potential reversion of *in vitro*-adaptive mutations, occurrence of immune escape mutations and other uncharacterized selection pressures that potentially select for sequence changes in addition to RNA structure re-acquisition (Figure 7). For example, the MNV3 clone was derived from an *in vitro* isolate in which a number of cell culture-adaptive changes may have occurred on initial isolation and passaging in RAW264.7 macrophages. These may have reverted on re-introduction into mice, as recently documented to have occurred in VP1 and NS7 genes (19). As a second possibility, there is no information on the MHC background of the mouse originally infected with MNV3 from which the isolate was originally obtained. Infection of mice in the current study with different class I haplotypes may have exerted selection pressure for immune escape at targeted MHC-I epitopes. These occur at high frequency and regularity in other persistent RNA virus infections, notably HCV and particularly well characterized in HIV-1 infections [reviewed in (46,47)]. Either of these explanations may account for the observation of convergent amino acid sequence changes in both mutated regions, although intriguingly only a subset of changes were shared in mice infected with WT and F1/F2 mutant viruses.

Although amino acid changes occurred in both WT and F1/F2-infected mice at similar frequencies, the latter showed evidence for substantially larger number of substitutions at synonymous sites (×3) than occurred in the WT virus in Region 1 (Figures 6 and 7A). As the permuted sequences used in the F1 and F2 constructs were identical in coding (and dinucleotide composition) to MNV3, this difference in mutation frequency observed in Region 1 discounts functional selection in F1/F2 mutants at the protein level or compositional selection (such as avoidance of certain dinucleotides such as CpG). The restriction of higher mutation rates to Region 1 may be a reflection of the lesser degree of RNA structure folding in Region 2 (MFED value of 3.9%), which may therefore be under reduced selection pressure to re-acquire RNA structure. A lesser degree of fitness loss by disruption of this region is apparent from the competition experiment, where similar levels of mutant and WT viruses were found in most mice up to 7 days after co-infection (Figure 8D). This observation contrasts with the much more rapid disappearance of F1 and F1/F2 mutants during this period (Figure 8C and E).

Without any other identified sequence constraints in these regions, the observations are instead consistent with a process of (partial) RNA secondary structure re-optimisation, a hypothesis supported by a consistent trend for increased MFED of the mutated sequences compared with the inoculum sequence in the F1 region, but not among WT control sequences (Figure 7B). Reacquisition of RNA secondary structure has been frequently observed after disruption of defined structured elements in a range of RNA viruses. In a remarkable series of studies in the 1990s, stem-loops that regulated coat protein expression in the MS2 phage were observed to rapidly rebuild after disruption (48,49). RNA structured elements in the genome of human immunodeficiency virus type 1 (the long stem-loop in the trans-activating region and the poly-A hairpin) would re-form on passaging of initially replication-impaired mutants over several months (50,51). Although there are potential analogies with the increased folding energies of MNV mutant sequences (Figure 7), different F1/F2-infected mice showed differing sequence changes, and it was bioinformatically problematic to predict specific structures in the absence of phylogenetic information (such as covariant sites) that are necessary to support a structure prediction.

We additionally acknowledge that the increases in MFED values were relatively small compared with the initial 7–10% reductions created by *in silico* mutagenesis. However, the process would, if continued, achieve WT levels over periods as short as 2–3 generations of mice. Both the rapid exclusion of destabilised viruses and the relatively short time frame for restoring RNA secondary structure provide a plausible evolutionary model for the maintenance of GORS in MNV populations in the wild.

### Interaction mechanisms

Despite the clear fitness difference phenotype of GORS-disrupted mutants characterized in the current study, the underlying mechanism(s) responsible for the phenotype remain largely undetermined. As previously discussed (8,9), the innate immune system represents the most likely point of interaction, where RNA structure probably influences RIG-I-like and Toll-like receptor-coupled mechanisms within the cell. Alternatively, GORS may modulate recognition by effector proteins such as protein kinase R (PKR) and RNAseL that are activated by dsRNA-binding domains, as documented from the binding and inhibition of PKR by adenovirus VA_I_ and Epstein–Barr virus EBER structured transcripts (52,53). Against this latter possibility, however, is the observation of equal sensitivity of WT and F1/F2 mutants to exogenous IFN-α and IFN-γ (Supplementary Figure S3). An observation that points towards an early effect of RNA structure disruption on host response in experimentally infected mice was the lower viral shedding in F1/F2-inoculated mice at day 1 (4-fold reduction compared with WT viruses). This difference was transient, with similar viral loads observed on day 3 and subsequently. This initial defect in replication potentially contributes towards for the rapid and almost complete elimination of mutant viruses (F1, F2 and F1/F2) when inoculated in competition with WT virus by day 3 (Figure 8). Evidence for an early replication defect of structure-disrupted mutants at a time before the evolution of systemic inflammatory or acquired immune responses points towards an effect at an early stage during recognition and/or the initial signalling by innate cellular responses. This conjecture is consistent with likely effects on virus recognition with pathogen-recognition receptors coupled to the interferon system.

Documenting what interactions are disrupted would be considerably assisted by the development of *in vitro* infection models where differences in replication phenotypes of or cellular responses to WT and GORS-disrupted mutants can be detected. It was also beyond the scope of the current study to investigate the basis for the observed fitness differences between the F1/F2 mutant and WT viruses in mice. Although various knockout mouse strains could have been experimentally infected, these likely modify the course of MNV infection [such as fatal infections in STAT-1 knockout mice (17)]. They therefore may no longer accurately model the natural history of infection in nature for which the contribution of RNA structure is likely most relevant. Among possible knockout targets to investigate, infection outcomes of MNV3 in mice with defects in MDA-5 and IFN receptors (IFNAR; IL28Rα) require prior characterization before they can be used for investigation of GORS-associated virus/host interactions.

### The evolution of large scale RNA structure

Observations of similar replicative capacity in cell culture, but a major fitness disadvantage *in vivo*, are consistent with predictions made from previous comparative bioinformatic analyses of its distribution in different virus groups (8,9). As demonstrated in Figure 1, even closely related viruses such as different norovirus species with similar replication strategies may vary greatly in their degree of RNA secondary structure (Figure 1), and among these, it is only noroviruses capable of persistence in an immunocompetent background (MNV variants infecting *M.musculus* and *A.sylvaticus*) that possess structured genomes. Infections with all other species are associated with acute and generally rapidly resolving acute gastroenteritis in immunocompetent humans, cows, pigs and a range of other mammalian species (54). In contrast to MNV, high levels of virus excretion and lack of sustained population immunity provide an alternative strategy for their longer term perpetuation, termed ‘virus durability’ (55,56). Associations between persistence and possession of structured genomes run through several other mammalian positive-stranded RNA virus families [e.g. *Flaviviridae*, *Picornaviridae*; (8,9)]. Although this might be construed as evidence for independent evolution of this trait among viruses adopting a persistent highly host-adapted life cycle, the converse has also been cogently argued (56). This postulates that the ancestral state for virus infections is persistence, best able to perpetuate in small host populations, but given the opportunity, acute pathogenic infections typically occur after cross-species transmission into a poorly adapted host but with a population size large enough to support an acute infection cycle. In this model, GORS and other adaptive changes that promote persistence are initially non-functional and are therefore lost, but perhaps regained after longer periods of adaptive evolutionary changes in a new host. That such closely related viruses as different norovirus species can differ so fundamentally in persistence and pathogenicity, correlated with possession of RNA structure and accessory genes that modulate host innate responses in the case of ORF4 in MNV (41), provides a potential example of this dynamic in nature.

For the first time, these studies demonstrate a phenotypic effect for extensive GORS *in vivo*. GORS plays a determining role in viral fitness and retention or acquisition of GORS is a fundamental evolutionary pressure operating directly at the RNA genome level. These studies have implications for our understanding of viral persistence, host-pathogen interactions and the evolution of RNA structure as a means of evading or subverting the host immune response. Indirectly, these studies have implications for our understanding of non-coding constraints on virus sequence drift and molecular clocks that make inherent assumptions about the evolutionary process that exclude secondary or higher-order RNA structures (57). Finally, these studies demonstrate that MNV is a suitable model system we and others can use to dissect the mechanistic role of GORS in viral persistence, a poorly understood characteristic of global viral pathogens such as HCV. In further characterizing the nature of GORS, ongoing studies are investigating the nature of the RNA structure and pairing characteristics, the potential existence of tertiary structures elements and whether permuted sequences selected for similar MFED values to those of native MNV sequences but with artificial RNA pairings can restore fitness *in vivo*. To this end, the availability of an animal model system in which the re-acquisition of GORS can be studied will be invaluable. These studies will provide further mechanistic insights into persistence mechanisms of RNA viruses and the pathways involved in virus recognition *in vivo*.

## SUPPLEMENTARY DATA

Supplementary Data are available at NAR Online: Supplementary Table 1 and Supplementary Figures 1–7.

## FUNDING

Wellcome Trust [WT087628MA] and a Wellcome Senior Fellowship [WT097997MA to I.G.]. Funding for open access charge: University of Edinburgh.

*Conflict of interest statement.* None declared.

## Supplementary Material

Supplementary Data
