# Supplementary material for: Influence of genome-scale RNA structure disruption on the replication of murine norovirus—similar replication kinetics in cell culture but attenuation of viral fitness in vivo
Source: Nucleic Acids Res. 2013 Apr 27;41(12):6316–31. doi: 10.1093/nar/gkt334 (PMC3695492; doi:10.1093/nar/gkt334)
Supplement: Supplementary Data [file supp_41_12_6316__index.html]

Influence of genome-scale RNA structure disruption on the replication of murine norovirus—similar replication kinetics in cell culture but attenuation of viral fitness in vivo — Influence of genome-scale RNA structure disruption on the replication of murine norovirus—similar replication kinetics in cell culture but attenuation of viral fitness in vivo — Supplementary Data 

# Influence of genome-scale RNA structure disruption on the replication of murine norovirus—similar replication kinetics in cell culture but attenuation of viral fitness *in vivo*

## Supplementary Data

files

**Files in this Data Supplement:**

- Supplementary Data - pdf file
