# Supplementary material for: Influence of genome-scale RNA structure disruption on the replication of murine norovirus—similar replication kinetics in cell culture but attenuation of viral fitness in vivo
Source: Nucleic Acids Res. 2013 Apr 27;41(12):6316–31. doi: 10.1093/nar/gkt334 (PMC3695492; doi:10.1093/nar/gkt334)
Supplement: Supplementary Data [file supp_gkt334_nar-00712-2013-File011.pdf]

SUPPLEMENTARY DATA

TABLE S1

PRIMERS USED FOR AMPLIFICATION OF MNV RNA SEQUENCES

Assay	Pos/Orient	Sequence (5'-3')
Region 1 PCR	1081F	GCATGGCCTTCGGTCTGACATC
	1121F	GCTTGCCTCCACCAACTCGG
	2500R	TGGTGGTAGATAGAGAAGGCTGT
	2650R	CGCCCTCGGCCCTTCTTGTTCTTACCCTTCTTGC
Region 2 PCR	2902F	GCAAGCCAATTGACTGGAATGTGGTTGGCCCC
	2961F	GACTACGGTGAGAAGATCAGCTTCG
	4128R	CGCGAATCATGGTGCCAAGGTCAGAGCC
	4155R	CACAGAAGGGGCCAAAAGCGCGAGC
Region 3 PCR	2342F	CATGGTTGAGAAAGTCAAGGAGC
	2412F	GCCCGGCTCCGCTGTTATTGC
	3099R	CGGTGCCCTTGGGTGGCGCCACATG
	3035R	CCATCCCGTGCCGAACTGTACGACG
Reverse transcription	7350R	AAAATGCATCTAAATACTACTAAAAGAAAAGAGC
Quantitative PCR	5380R	GAACCTCCATGTTCCCAACCCAGCCGGTGTACATGG
	5028S	CCGCAGGAACGCTCAGCAGT
	5177R	GCAAGAGCCGCGCCAGCCACGGGCTGAATGGGGACGGCCT
	5077R-Probe	CTGCGCCATCACTCAT
	18sS	GTAACCCGTTGAACCCCATTC
	18sR	ACCATCCAATCGGTAGTAGCG
	5275F	ACCCCAGGTGAAATACTGTTTG
	5452R	TGGGAAAATAGGGTGGTACAAG
Allele-specific PCR (Region 1)	2141F	TGCTGGCAAGGTTACCGCCTTC
	2650R	CGCCCTCGGCCCTTCTTGTTCTTACCCTTCTTGC
	2206F	CAGCGATTGGCTACAAGGCCATGGGCTGCACCTG
	2401R_F1	CGCCTGACGCAGTTCACCAGTG
Digest PCR (Region 1)	1081F	GCATGGCCTTCGGTCTGACATC
	1121F	GCTTGCCTCCACCAACTCGG
	1522R	GGCCTGCCAGAGACCATAATCAC
	2650R	CGCCCTCGGCCCTTCTTGTTCTTACCCTTCTTGC
Allele-specific PCR (Region 2)	2902F	GCAAGCCAATTGACTGGAATGTGGTTGGCCCC
	4155R	CACAGAAGGGGCCAAAAGCGCGAGC

	3678F	GCTTACCTGGGYTCCAAGGATGAGAG
	3846R_F2	GGGCTCCAGCGTATTCTCAAGT
Digest PCR (region 2)	2902F	GCAAGCCAATTGACTGGAATGTGGTTGGCCCC
	3678F	GCTTACCTGGGYTCCAAGGATGAGAG
	4128R	CGCGAATCATGGTGCCAAGGTCAGAGCC
	4155R	CACAGAAGGGGCCAAAAGCGCGAGC

FIGURE S1

A)

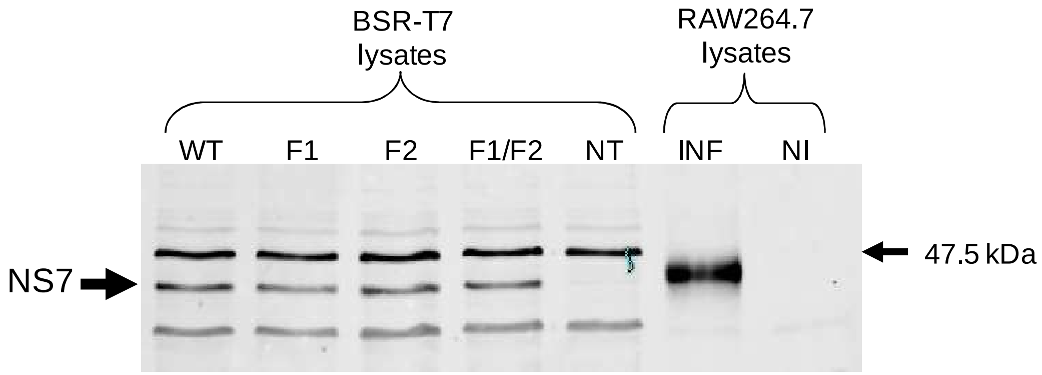

B)

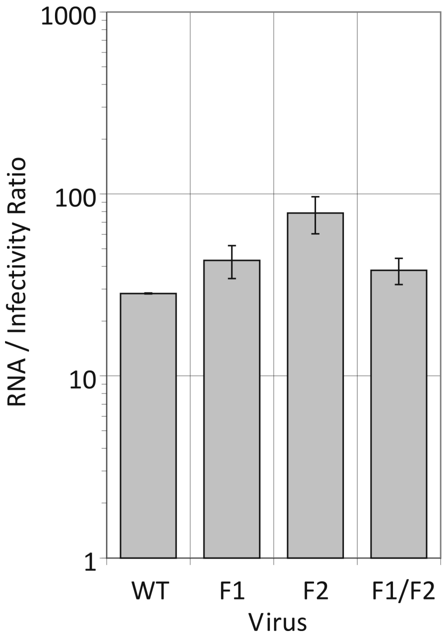

(A) Western blot detection of NS7 (viral RNA polymerase) expressed in BSRT-7 cells after transfection with WT and RNA-structure disrupted mutant cDNA. Infected and non-infected RAW264.7 lysates acted as an assay control. (B) Infectivity to RNA ratios was measured by extracting RNA from equal amounts of viral particles and quantifying viral copy numbers by qPCR.

FIGURE S2

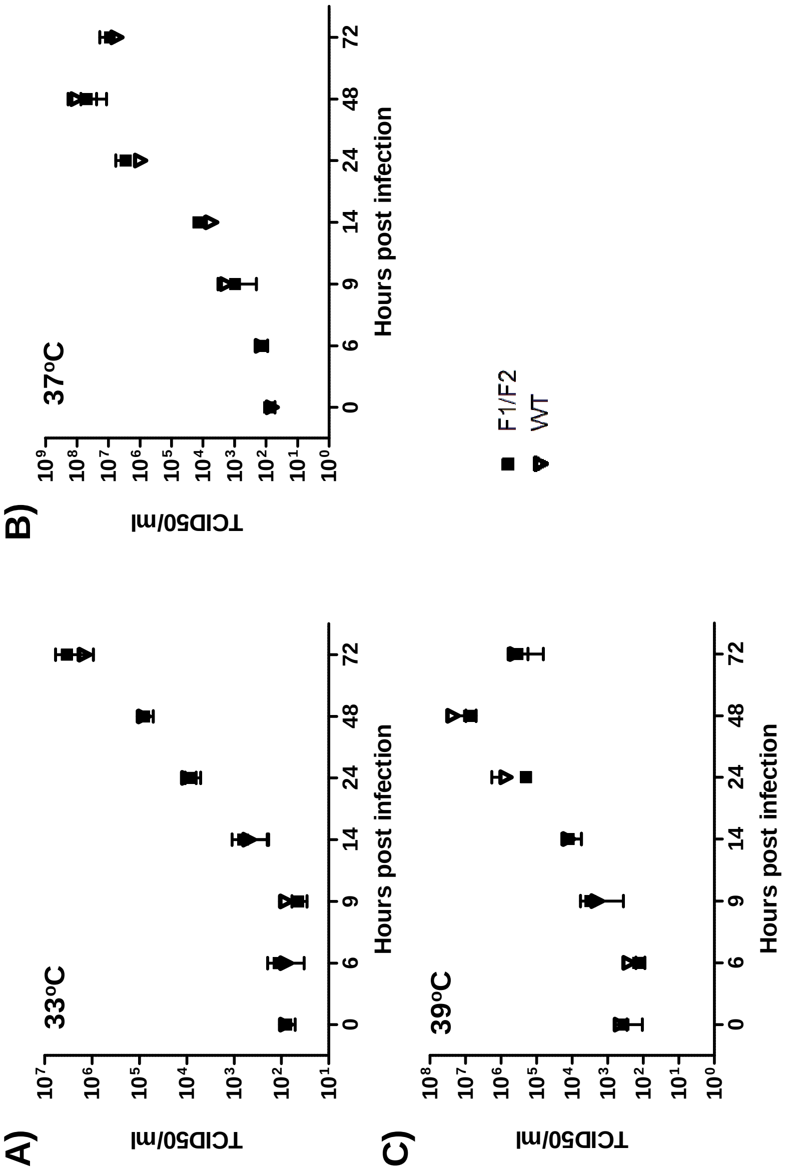

Influence of incubation temperature on the replicaion of WT and F1/F2 viruses. Multistep growth curves (m.o.i of 0.01) were performed in RAW264.7 cells at (A) 33°C, (B) 37°C and (C) 39°C. Infectivity titres expressed as TCID50s / ml were determined at several time points post-infection..

FIGURE S3A

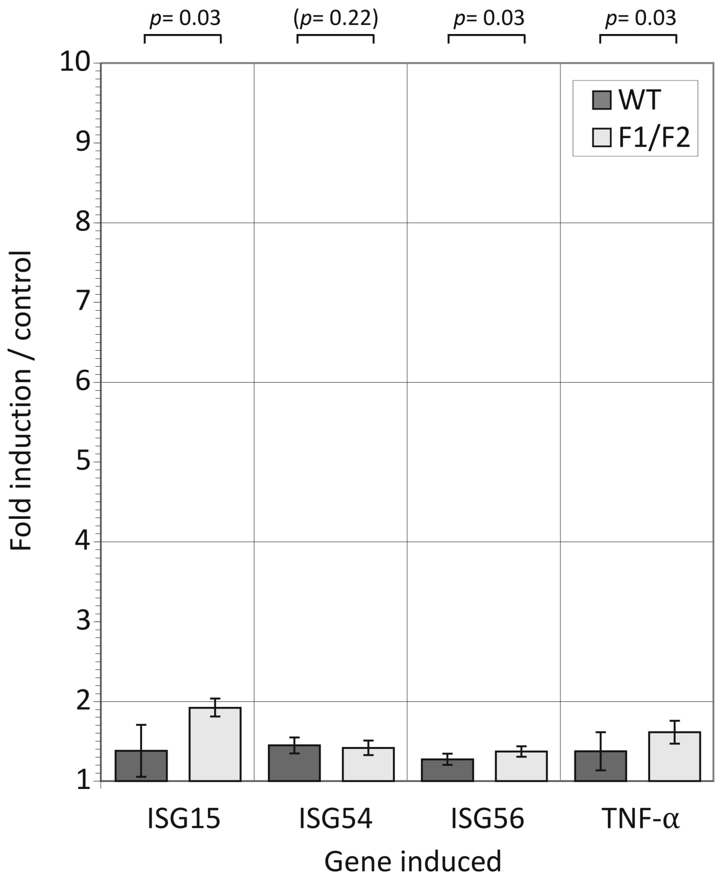

Low level induction of ISGs 15, 54 and 56 and TNF-α in RAW264.7 infected with MNV3 WT or F1/F2 mutants at an m.o.i. of 1 and assaying mRNA levels by qPCR at 8 hours. All assays were performed in triplicate (mean values and SEMs shown by bars and error bars).

FIGURE S3B

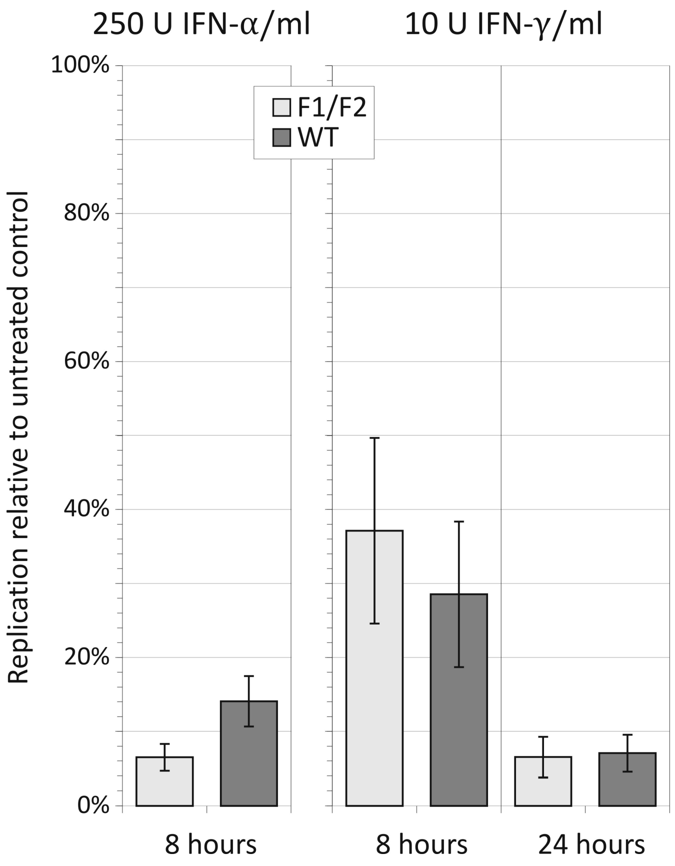

Sensitvity of WT and F1/F2 variants of MNV to IFN-α and IFN-γ shown as the proportion of RNA levels in IFN-treated cells compared to the untreated control. No significant differences between replication rates of WT and F1/F2 variants were detected (student T test, $p > 0.05$ for all comparisons)

FIGURE S4

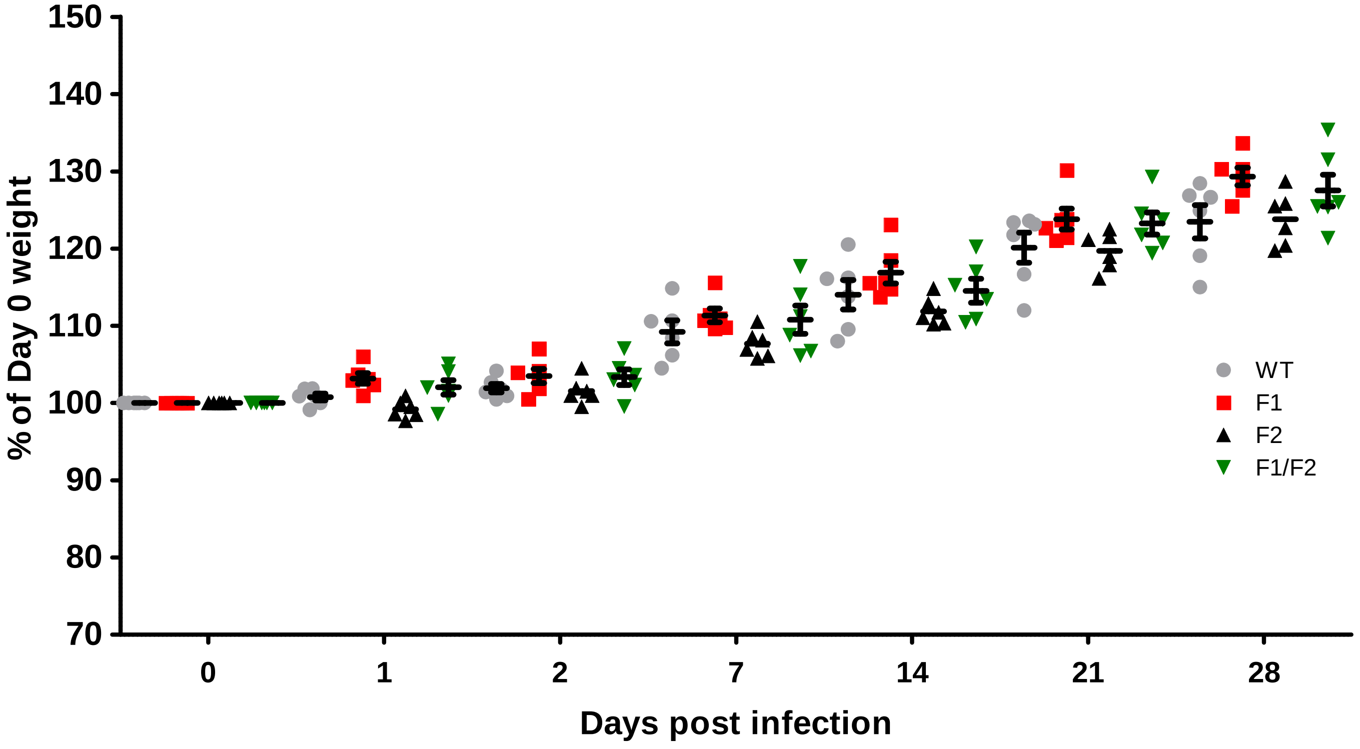

Change in weight of mice infected with F1, F2 and F1/F2 mutant viruses compared to those infected with WT virus. Bars show means and SEMs for weight distributions in each category.

FIGURE S5

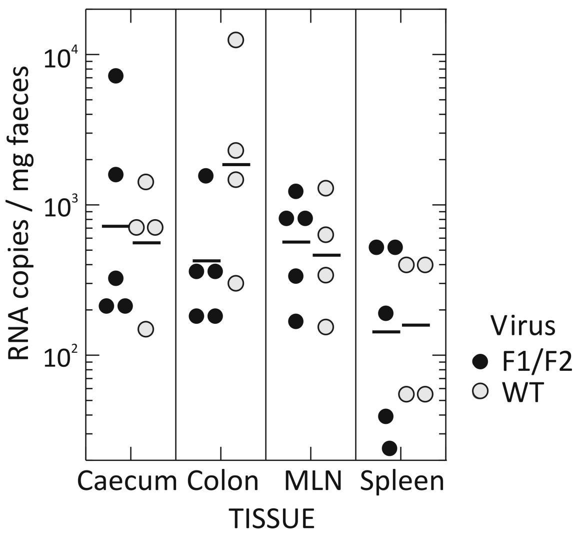

Viral loads in tissues collected 9 months post-infection with WT and F1/F2 mutant viruses. No significant differences in viral loads were detected in mice infected mutant and WT viruses in any tissue.

FIGURE S6

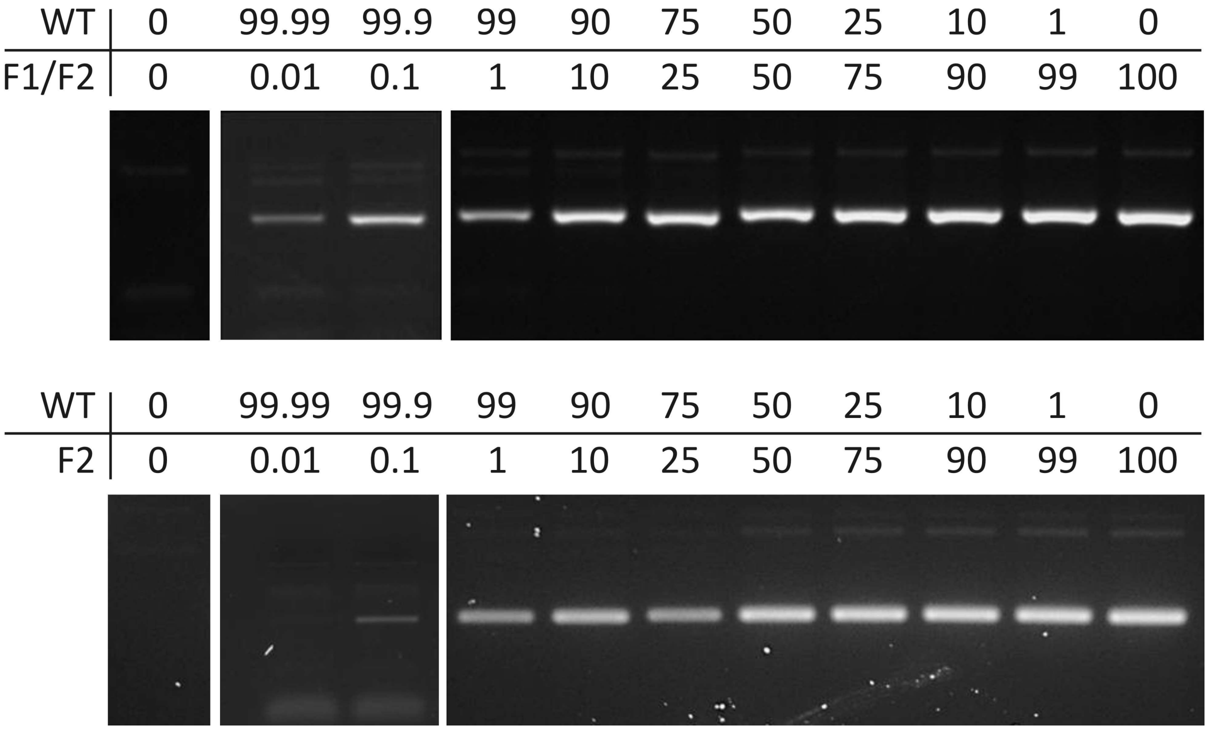

Sensitivity of mutant-specific PCR for different input proportions of WT and F1 or F2 mutant viruses (proportions indicated above gel images).

FIGURE S7

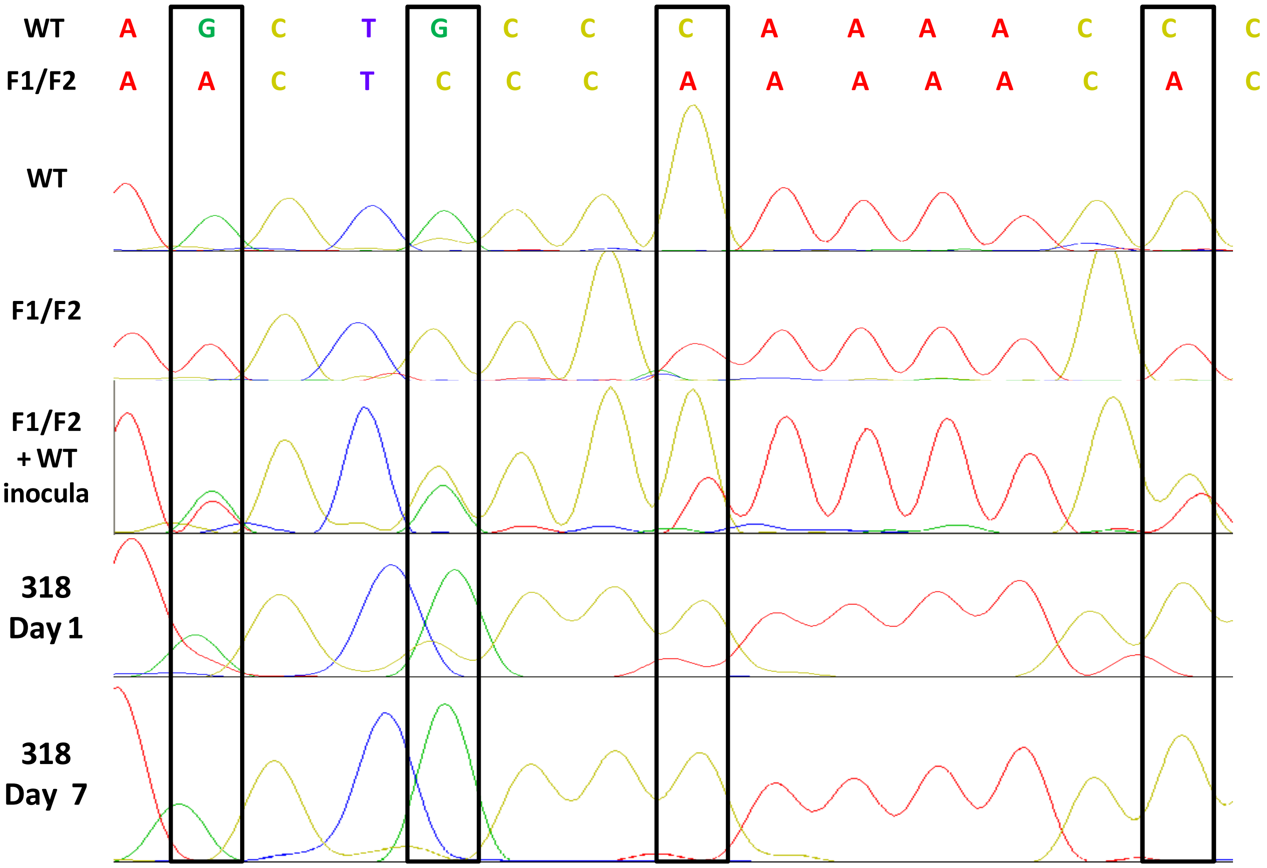

Representative chromatograms of Sanger sequencing reactions of regions of F1 variable in sequence between WT and mutant viruses (genome position 2368-2381; polymorphic sites shown in boxes). This compares sequence traces of WT and F1 sequences with the inoculum strain (50%/50% $TCID_{50}$ ratio of WT and F1/F2) and with viral RNA extracted from faecal samples of co-infected mice collected on days 1 and 7 post inoculation.

SEQUENCE SUPPLEMENT

Sequences of MNV recovered from mice infected with WT and F1/F2 mutants.

Region 1

```
>F1+2
GCCTCCACCAACTCGGCCCTCAAGGCCGCGCAGGAGATGGGGAAGTTTGC
GATTGAGGTTTTCAAGCAAATCATGGCCTGGATCTGGCCGTCCGAGGACC
CGGTACCCGCCCTGCTCTCAAACATGGAGCAGGCTGTCATCAAGAACGAG
TGCCAGCTCGAGAACCAACTCACCACCATGTTGAGGGACCGAAATGCAGG
AGCAGAGTTCTTGAAGGCTCTAGATGAAGAAGAGCAGGAGGTTCGCAAGA
TTGCGGCCAAGTGTGGCAATTCTGCCACCACCGGCACGACTAATGCTCTC
TTGGCTAGGATCAGCATGGCCCGCGCTGCCTTTGAGAAGGCACGAGCCGA
GCAGACCTCTCGCGTCCGCCCAGTTGTGATTATGGTCTCTGGCAGGCCAG
GCATTGGGAAAACTTGCTTCTGCCAGAACCTAGCAAAAAGGATCGCGGCC
TCGCTGGGCGACGAGACCTCAGTTGGCATCATCCCGCGTGCGGACGTGGA
CCACTGGGACGCTTACAAGGGCGCCAGGGTGGTACTGTGGGACGACTTTG
GGATGGACAATGTAGTCAAGGATGCCCTGCGCCTGCAGATGCTCGCAGAC
ACATGCCCCGTCACTCTTAATTGTGACAGGATTGAGAACAAAGGAAAGAT
GTTTGACTCTCAGGTCATCATCATCACCACCAACCAGCAGACACCCGTCC
CCTTGGATTATGTCAACCTTGAGGCCGTTTGCCGGCGCATCGATTTCCTG
GTCTATGCTGAGAGCCCCGTGGTCGACGCTGCGCGAGCCAGGTCCCCTGG
TGATGTGGCCGCTGTGAAAGCCGCCATGAGGCCCGATTACAGCCACATCA
ACTTCATTTTGGCCCCCCAGGGCGGCTTTGACCGCCAGGGCAACACACCC
TATGGCAAGGGGGTCACGAAGATCATCGGGGCCACTGCCCTCTGCGCCAG
GGCTGTTGCGCTTGTGCATGAGCGCCATGATGATTTCGGTCTGCAGAACA
AAGTCTATGATTTTGATGCTGGCAAGGTTACCGCATTCAAGACCATGGCT
GCTGATGCGGGCATCCCCTGGTATAAGATGGCGGCCATTGGCTACAAGGC
GATGGGCTGCACTTGCGTGGAGGAAGCCATGAACCTGCTCAAGGACTATG
AGGTAGCCCCCTGCCAGGTCATCTACAACGGTGCCACCTACAACGTCAGC
TGCATCAAGGGGGCCCCCATGGTTGAGAAAGTCAAGGAGCCTGAACTCCC
AAAAACACTGGTGAACTGCGTCAGGCGGATTAAGGAGGCCCGGCTCCGCT
GTTATTGCAGGATGGCCACAGACGTCATCACCTCCATCCTGCAGGCGGCT
GGGACAGCCTTCTCTATCTACCACCA
>MNV1
GCTTCCACCAACTCGGCTCTCAAGGCCGCCCAAGAGATGGGCAAGTTCGC
CATAGAGGTCTTCAAGCAAATTATGGCCTGGATCTGGCCCTCTGAGGACC
CAGTGCCAGCCCTCTTATCCAACATGGAGCAGGCCATCATTAAGAATGAG
TGTCAACTGGAGAACCAACTCACGGCCATGTTGCGGGATCGCAACGCAGG
GGCTGAATTCCTAAGGTCCCTTGATGAGGAGGAGCAGGAAGTCCGCAAGA
TCGCAGCTAAGTGCGGCAACTCGGCCACCACTGGAACCACCAACGCTCTG
CTGGCCAGGATCAGCATGGCCCGCGCGGCCTTTGAGAAAGCTCGCGCTGA
ACAGACCTCCCGAGTCCGCCCTGTGGTGATCATGGTCTCAGGCAGGCCCG
GGATCGGGAAAACCTGCTTTTGCCAAAACCTGGCCAAGAGGATTGCTGCG
TCCCTGGGTGATGAGACCTCTGTTGGCATCATACCACGCGCTGATGTCGA
CCACTGGGATGCTTACAAGGGAGCCAGAGTGGTTCTCTGGGATGATTTCG
GCATGGACAACGTGGTGAAGGATGCACTGAGGCTTCAGATGCTTGCCGAC
ACGTGCCCAGTGACACTCAATTGTGACAGGATTGAGAACAAGGGAAAGAT
GTTTGACTCTCAGGTCATTATCATCACCACAAATCAACAAACCCCCGTGC
CCCTGGACTATGTCAACCTGGAGGCGGTCTGCCGCCGCATAGATTTCCTG
GTTTATGCTGAGAGCCCTGTTGTTGATGATGCTCGGGCCAGAGCCCCTGG
CGATGTGAATGCAGTGAAAGCTGCCATGAGGCCCGATTACAGCCACATCA
ATTTCATCTTGGCACCGCAGGGCGGCTTTGACCGTCAGGGAAACACCCCC
TACGGTAAGGGCGTCACCAAGATCATTGGCGCCACTGCTCTTTGCGCGAG
AGCGGTTGCTCTTGTCCATGAGCGCCATGATGATTTCGGCCTCCAGAACA
AGGTCTATGACTTTGATGCCGGCAAGATCACCGCCTTCAAGNCCATGGCG
GCTGACGCCGGCATTCCATGGTACAAAATGGCAGCTATTGGGTGCAAAGC
AATGGGGTGCACCTGTGTAGAGGAGGCCATGCATTTACTTAAGGATTATG
AGGTGGCTCCCTGTCAGGTGATCTACAATGGTGCCACCTATAATGTGAGC
```

TGCATCAAGGGTGCCCCAATGGTTGAAAAGGTCAAGGAGCCTGAATTGCC
CAAAACACTTGTCAACTGTGTCAGAAGGATAAAGGAGGCCCGCCTCCGCT
GCTACTGTAGGATGGCTGCTGACGTCATCACGTCCATTCTGCAGGCGGCC
GGCACGGCCTTCTCTATTTACCACCA
>pT7:MNV\3
GCCTCCACCAACTCGGCCCTCAAGGCCGCCCAGGAGATGGGCAAGTTTGC
GATTGAGGTTTTCAAACAAATCATGGCATGGATCTGGCCCTCTGAGGACC
CGGTGCCCGCTCTGCTTTCCAACATGGAGCAGGCTGTCATCAAGAACGAG
TGCCAGCTTGAGAACCAACTCACGACCATGTTGCGGGACCGCAACGCTGG
AGCTGAGTTCTTGAAGGCACTCGACGAAGAAGAGCAAGAGGTCCGGAAAA
TTGCAGCCAAGTGTGGAAACTCTGCCACCACGGGCACAACCAACGCCCTG
TTGGCCAGGATTAGCATGGCTCGCGCAGCCTTTGAGAAGGCTCGCGCTGA
GCAGACTTCCCGGGTTCGCCCCGTAGTGATTATGGTCTCTGGCAGGCCCG
GGATCGGGAAAACTTGTTTTTGCCAGAACCTAGCAAAGAGAATTGCTGCC
TCCCTCGGGGATGAGACCTCAGTCGGCATCATACCGCGCGCCGACGTGGA
CCACTGGGATGCCTACAAGGGCGCCAGGGTGGTCCTCTGGGATGATTTTG
GCATGGACAATGTGGTGAAGGATGCACTGCGGCTGCAGATGCTTGCTGAC
ACATGCCCCGTCACGCTCAACTGTGACAGGATTGAGAACAAGGGGAAGAT
GTTTGATTCTCAAGTTATCATCATCACCACGAACCAGCAGACCCCAGTGC
CTTTGGATTATGTCAACCTTGAGGCGGTCTGCCGCCGCATAGACTTCCTG
GTCTATGCCGAGAGCCCCGTGGTCGACGCTGCCCGAGCCAGGTCACCGGG
CGACGTGGCCGCTGTCAAGGCCGCTATGAGGCCAGACTACAGCCATATCA
ATTTCATCTTGGCACCGCAGGGTGGCTTTGACCGGCAGGGCAACACCCCC
TACGGCAAGGGCGTCACTAAGATCATCGGCGCCACCGCGCTCTGTGCAAG
GGCGGTCGCTCTTGTCCATGAGCGTCATGATGATTTTGGCCTCCAGAACA
AAGTCTATGATTTTGATGCTGGCAAGGTTACCGCCTTCAAGACCATGGCG
GCTGATGCCGGCATCCCCTGGTACAAGATGGCAGCGATTGGCTACAAGGC
CATGGGCTGCACCTGCGTGGAGGAAGCCATGAACCTACTCAAGGACTATG
AGGTGGCCCCGTGCCAGGTGATCTACAACGGAGCCACCTACAATGTCAGC
TGCATCAAGGGGGCGCCCATGGTTGAGAAAGTCAAGGAGCCTGAGCTGCC
CAAAACCCTAGTCAATTGTGTCAGGAGGATCAAGGAGGCCCGCCTCCGCT
GCTATTGCAGGATGGCCACAGATGTCATCACCTCCATCCTGCAGGCGGCT
GGGACAGCCTTCTCTATCTACCACCA
>302_Cae
GCCTCCACCAACTCGGCCCTCAAGGCCGCGCAGGAGATGGGGAAGTTTGC
GATTGAGGTTTTCAAGCAAATCATGGCCTGGATCTGGCCGTCCGAGGACC
CGGTACCCGCCCTGCTCTCAAACATGGAGCAGGCTGTCATCAAGAACGAG
TGCCAGCTCGAGAACCAACTCACCACCATGTTGAGGGACCGAAATGCAGG
AGCAGAGTTCTTGAAGGCTCTAGATGAAGAAGAGCAGGAGGTTCGCAAGA
TTGCGGCCAAGTGTGGCAATTCTGCCACCACCGGCACGACTAATGCTCTC
TTGGCTAGGATCAGCATGGCCCGCGCTGCCTTTGAGAAGGCACGAGCTGA
GCAGACCTCTCGCGTCCGCCCAGTTGTGATTATGGTCTCTGGCAGGCCAG
GCATTGGGAAAACTTGCTTCTGCCAGAACCTAGCAAAAAGGATCGCGGCC
TCGCTGGGCGACGAGACCTCAGTTGGCATCATCCCGCGTGCGGACGTGGA
CCACTGGGACGCTTACAAGGGCGCCAGGGTGGTACTGTGGGACGACTTTG
GGATGGACAATGTAGTCAAGGATGCCCTGCGCCTGCAGATGCTCGCAGAC
ACATGCCCCGTCACTCTCAATTGTGACAGGATTGAGAACAAAGGAAAGAT
GTTTGACTCTCAGGTCATCATCATCACCACCAACCAGCAGACACCCGTCC
CCTTGGATTATGTCAACCTTGAGGCCGTTTGCCGGCGCATTGATTTCCTG
GTCTATGCTGAGAGCCCCGTGGTCGATGCTGCGCGAGCCAGGTCCCCTGG
TGATGTAGCCGCTGTGAAAGCCGCCATGAGGCCCGATTACAGCCACATCA
ACTTCATTTTGGCCCCCCAGGGCGGCTTTGACCGCCAGGGCAACACACCC
TATGGCAAGGGGGTCACGAAGATCATCGGGGCCACTGCCCTCTGCGCCAG
GGCTGTTGCGCTTGTGCATGAGCGCCATGATGATTTCGGTCTGCAGAACA
AAGTCTATGATTTTGATGCTGGCAAGGTTACCGCATTCAAGACCATGGCT
GCTGATGCGGGCATCCCCTGGTATAAGATGGCGGCCATTGGCTACAAGGC
GATGGGCTGCACTTGCGTGGAAGAAGCCATGAACCTGCTCAAGGACTATG
AGGTAGCCCCCTGCCAGGTCATCTACAACGGTGCCACCTACAACGTCAGC
TGCATCAAGGGGGCCCCCATGGTTGAGAAAGCCAAGGAGCCTGAACTCCC
AAAAACACTGGTGAACTGCGTCAGGCGGATTAAGGAGGCCCGGCTCCGCT
GTTATTGCAGGATGGCCACAGACGTCATCACCTCCNNNNNNNNNNNNNNN

NNNNNNNNNNNNNNNNNNNNNNNNNN
>302_Colon
GCCTCCACCAACTCGGCCCTCAAGGCCGCGCAGGAGATGGGGAAGTTTGC
GATTGAGGTTTTCAAGCAAATCATGGCCTGGATCTGGCCGTCCGAGGACC
CGGTACCCGCCCTGCTCTCAAACATGGAGCAGGCTGTCATCAAGAACGAG
TGCCAGCTCGAGAACCAACTCACCGCCATGTTGAGGGACCGAAATGCAGG
AGCAGAGTTCTTGAAGGCTCTAGATGAAGAAGAGCAGGAGGTTCGCAAGA
TTGCGGCCAAGTGTGGCAATTCTGCCACCACCGGCACGACCAATGCTCTC
TTGGCTAGGATCAGCATGGCCCGCGCTGCCTTTGAGAAGGCACGAGCCGA
GCAGACCTCTCGCGTCCGCCCGGTTGTGATTATGGTCTCTGGCAGGCCAG
GCATTGGGAAAACTTGCTTCTGCCAGAACCTAGCAAAAAGGATCGCGGCC
TCGCTGGGCGACGAGACCTCAGTTGGCATCATCCCGCGTGCGGACGTGGA
CCACTGGGACGCTTACAAGGGCGCCAGGGTGGTACTGTGGGACGACTTTG
GGATGGACAATGTAGTCAAGGATGCCCTGCGCCTGCAGATGCTCGCAGAC
ACATGCCCCGTCACTCTTAATTGTGACAGGATTGAGAACAAAGGAAAGAT
GTTTGACTCTCAGGTCATCATCATCACCACCAACCAGCAGACACCCGTCC
CCTTGGATTATGTCAACCTTGAGGCCGTTTGCCGGCGCATCGATTTCCTG
GTCTATGCTGAGAGCCCCGTGGTCGAYGCTGCGCGAGCCAGGTCCCCTGG
TGATGTGGCCGCTGTGAAAGCCGCCATGAGGCCCGATTACAGCCACATCA
ACTTCATTTTGGCCCCCCAGGGCGGCTTTGACCGCCAGGGCAACACACCC
TATGGCAAGGGGGTCACGAAGATCATCGGGGCCACTGCCCTCTGCGCCAG
GGCTGTTGCGCTTGTGCATGAGCGCCATGATGATTTCGGTCTGCAGAACA
AAGTCTATGATTTTGATGCTGGCAAGGTTACCGCATTCAAGACCATGGCT
GCTGATGCGGGCATCCCCTGGTATAAGATGGCGGCCATTGGCTACAAGGC
GATGGGCTGCACTTGTGTGGAGGAAGCCATGAACCTGCTCAAGGACTATG
AGGTAGCCCCCTGCCAGGTCATCTACAACGGTGCCACCTACAACGTCAGC
TGCATCAAGGGGGCCCCCATGGTTGAGAAAGTCAAGGAGCCTGAACTCCC
AAAAACACTGGTGAACTGCGTCAGGCGGATTAAGGAGGCCCGGCTCCGCT
GTTATTGCAGGATGGCCACAGACGTCATCACCTCCATCCTGCAGGCGGCT
GGGACAGCCTTCTCTATCTACCACCA
>302_MLN
GCCTCCACCAACTCGGCCCTCAAGGCCGCGCAGGAGATGGGGAAGTTTGC
GATTGAGGTTTTCAAGCAAATCATGGCCTGGATCTGGCCGTCCGAGGACC
CGGTACCCGCCCTGCTCTCAAACATGGAGCAGGCTGTCATCAAGAACGAG
TGCCAGCTCGAGAACCAACTCACCACCATGTTGAGGGACCGAAATGCAGG
AGCAGAGTTCTTGAAGGCTCTAGATGAAGAAGAGCAGGAGGTTCGCAAGA
TTGCGGCCAAGTGTGGCAATTCTGCCACCACCGGCACGACTAATGCTCTC
TTGGCTAGGATCAGCATGGCCCGCGCTGCCTTTGAGAAGGCACGAGCTGA
GCAGACCTCTCGCGTCCGCCCGGTTGTGATTATGGTCTCTGGCAGGCCAG
GCATTGGGAAAACTTGCTTCTGCCAGAACCTAGCAAAAAGGATCGCGGCC
TCGCTGGGCGACGAGACCTCAGTTGGCATCATCCCGCGTGCGGACGTGGA
CCACTGGGACGCTTACAAGGGCGCCAGGGTGGTACTGTGGGACGACTTTG
GGATGGACAATGTAGTCAAGGATGCCCTGCGCCTGCAGATGCTCGCAGAC
ACATGCCCCGTTACTCTTAATTGTGACAGGATTGAGAACAAAGGAAAGAT
GTTTGACTCTCAGGTCATCATCATCACCACCAACCAGCAGACACCCGTCC
CCTTGGATTATGTCAACCTTGAGGCCGTTTGCCGGCGCATCGATTTCCTG
GTCTATGCTGAGAGCCCCGTGGTCGATGCTGCGCGAGCCAGGTCCCCTGG
TGATGTGGCCGCTGTGAAAGCCGCCATGAGGCCCGATTACAGCCACATCA
ACTTCATTTTGGCCCCCCAGGGCGGCTTTGACCGCCAGGGCAACACACCC
TATGGCAAGGGGGTCACGAAGATCATCGGGGCCACTGCCCTCTGCGCCAG
GGCTGTTGCGCTTGTGCATGAGCGCCATGATGATTTCGGTCTGCAGAACA
AAGTCTATGATTTTGATGCTGGCAAGGTTACCGCATTCAAGACCATGGCT
GCTGATGCGGGTATCCCCTGGTATAAGATGGCGGCCATTGGCTACAAGGC
GATGGGCTGCACTTGCGTGGAAGAAGCCATGAACCTGCTCAAGGACTATG
AGGTAGCCCCCTGCCAGGTCATCTACAACGGTGCCACCTACAACGTCAGC
TGCATCAAGGGGGCCCCCATGGTTGAGAAAGCCAAGGAGCCTGAACTCCC
AAAAACACTGGTGAACTGCGTCAGGCGGATTAAGGAGGCCCGGCTCCGCT
GTTATTGCAGGATGGCCACAGACGTCATCACCTCCATCCTGCAGGCGGCT
GGGACAGCCTTCTCTATCTACCACCA
>303_M8
GCCTCCACCAACTCGGCCCTCAAGGCCGCGCAGGAGATGGGGAAGTTTGC

GATTGAGGTTTTCAAGCAAATCATGGCCTGGATCTGGCCGTCCGAGGACC
CGGTACCCGCCCTGCTCTCAAACATGGAGCAGGCTGTCATCAAGAACGAG
TGCCAGCTCGAGAACCAACTCACCGCCATGTTGAGGGACCGAAATGCAGG
AGCAGAGTTCTTGAAGGCTCTAGATGAAGAAGAGCAGGAGGTTCGCAAGA
TTGCGGCCAAGTGTGGCAATTCTGCCACCACCGGCACGACCAATGCTCTC
TTGGCTAGGATCAGCATGGCCCGCGCTGCCTTTGAGAAGGCACGAGCCGA
GCAGACCTCTCGCGTCCGCCCAGTTGTGATTATGGTCTCTGGCAGGCCAG
GCATTGGGAAAACTTGCTTCTGCCAGAACCTAGCAAAAAGGATCGCGGCC
TCGCTGGGCGACGAGACCTCAGTTGGCATCATCCCGCGTGCGGACGTGGA
CCACTGGGACGCTTACAAGGGCGCCAGGGTGGTATTGTGGGACGACTTTG
GGATGGACAATGTAGTCAAGGATGCCCTGCGCCTGCAGATGCTNGCAGAC
ACATGCCCCGTCACTNTTAATTGTGACAGGATTGAGAACAAAGGAAAGAT
GTTTGACTCTCAGGTCATCATCATCACCACCAACCAGCAGACACCCGTCC
CCTTGGATTATGTCAACCTTGAGGCCGTTTGCCGGCGCATCGATTTCCTG
GTCTATGCTGAGAGCCCCGTGGTCGACGCTGCGCGAGCCAGGTCCCCTGG
TGATGTGGCCGCTGTGAAAGCCGCCATGAGGCCCGATTACAGCCACATCA
ACTTCATTTTGGCCCCCCAGGGCGGCTTTGACCGCCAGGGCAACACACCC
TATGGCAAGGGGGTCACGAAGATCATCGGGGCCACTGCCCTCTGCGCCAG
GGCTGTTGCGCTTGTGCATGAGCGCCATGATGATTTCGGTCTGCAGAACA
AAGTCTATGATTTTGATGCTGGCAAGGTTACCGCATTCAAGACCATGGCT
GCTGATGCGGGCATCCCCTGGTATAAGATGGCGGCCATTGGCTACAAGGC
GATGGGCTGCACTTGTGTGGAGGAAGCCATGAACCTGCTCAAGGACTATG
AGGTAGCCCCCTGCCAGGTCATCTACAACGGTGCCACCTACAACGTCAGC
TGCATCAGGGGGGCCCCCATGGTTGAGAAAGTCAAGGAGCCTGAACTCCC
AAAAACACTGGTGAACTGCGTCAGGCGGATTAAGGAGGCCCGGCTCCGCT
GTTATTGCAGGATGGCCACAGACGTCATCACCTCCATCCTGCANNNNNNN
NNNNNNNNNNNNNNNNNNNNNNNNN
>303_Cae
GCCTCCACCAACTCGGCCCTCAAGGCCGCGCAGGAGATGGGGAAGTTTGC
GATTGAGGTTTTCAAGCAAATCATGGCCTGGATCTGGCCGTCCGAGGACC
CGGTACCCGCCCTGCTCTCAAACATGGAGCAGGCTGTCATCAAGAACGAG
TGCCAGCTCGAGAACCAACTCACCGCCATGTTGAGGGACCGAAATGCAGG
AGCAGAGTTCTTGAAGGCTCTAGATGAAGAAGAGCAGGAGGTTCGCAAGA
TTGCGGCCAAGTGTGGCAATTCTGCCACCACCGGCACGACCAATGCTCTC
TTGGCTAGGATCAGCATGGCCCGCGCTGCCTTTGAGAAGGCACGAGCCGA
GCAGACCTCTCGCGTCCGCCCAGTTGTGATTATGGTCTCTGGCAGGCCAG
GCATTGGGAAAACTTGCTTCTGCCAGAACCTAGCAAAAAGGATCGCGGCC
TCGCTGGGCGACGAGACCTCAGTTGGCATCATCCCGCGTGCGGACGTGGA
CCACTGGGACGCTTACAAGGGCGCCAGGGTGGTATTGTGGGACGACTTTG
GGATGGACAATGTAGTCAAGGATGCCCTGCGCCTGCAGATGCTCGCAGAC
ACATGCCCCGTCACTCTTAATTGTGACAGGATTGAGAACAAAGGAAAGAT
GTTTGACTCTCAGGTCATCATCATCACCACCAACCAACAGACACCCGTCC
CCTTGGATTATGTCAACCTTGAGGCCGTTTGCCGGCGCATCGATTTCCTG
GTCTATGCTGAGAGCCCCGTGGTCGAYGCTGCGCGAGCCAGGTCCCCTGG
TGATGTGGCCGCTGTGAAAGCCGCCATGAGGCCCGATTACAGCCACATCA
ACTTCATTTTGGCCCCCCAGGGCGGCTTTGACCGCCAGGGCAACACACCC
TATGGCAAGGGGGTCACGAAGATCATCGGGGCCACTGCCCTCTGCGCCAG
GGCTGTTGCGCTTGTGCATGAGCGCCATGATGATTTCGGTCTGCAGAACA
AAGTCTATGATTTTGATGCTGGCAAGGTTACCGCATTCAAGACCATGGCT
GCTGATGCGGGCATCCCCTGGTATAAGATGGCGGCCATTGGCTACAAGGC
GATGGGCTGCACTTGTGTGGAGGAAGCCATGAACCTGCTCAAGGACTATG
AGGTAGCCCCCTGCCAGGTCATCTACAACGGTGCCACCTACAACGTCAGC
TGCATCAGGGGGGCCCCCATGGTTGAGAAAGTCAAGGAGCCTGAACTCCC
AAAAACACTGGTGAACTGCGTCAGGCGGATTAAGGAGGCCCGGCTCCGCT
GTTATTGCAGGATGGCCACAGACGTCATCACCTCCATCCTGCAGGCGGCT
GGGACAGCCTTCTCTATCNNNNNNNN
>303_Colon
NNNNNNNNNNACTCGGCCCTCAAGGCCGCGCAGGAGATGGGGAAGTTTGC
GATTGAGGTTTTCAAGCAAATCATGGCCTGGATCTGGCCGTCCGAGGACC
CGGTACCCGCCCTGCTCTCAAACATGGAGCAGGCTGTCATCAAGAACGAG
TGCCAGCTCGAGAACCAACTCACCGCCATGTTGAGGGACCGAAATGCAGG

AGCAGAGTTCTTGAAGGCTCTAGATGAAGAAGAGCAGGAGGTTCGCAAGA
TTGCGGCCAAGTGTGGCAATTCTGCCACCACCGGCACGACCAATGCTCTC
TTGGCTAGGATCAGCATGGCCCGCGCTGCCTTTGAGAAGGCACGAGCCGA
GCAGACCTCTCGCGTCCGCCCAGTTGTGATTATGGTCTCTGGCAGGCCAG
GCATTGGGAAAACTTGCTTCTGCCAGAACCTAGCAAAAAGGATCGCGGCC
TCGCTGGGCGACGAGACCTCAGTTGGCATCATCCCGCGTGCGGACGTGGA
CCACTGGGACGCTTACAAGGGCGCCAGGGTGGTATTGTGGGACGACTTTG
GGATGGACAATGTAGTCAAGGATGCCCTGCGCCTGCAGATGCTCGCAGAC
ACATGCCCCGTCACTCTTAATTGTGACAGGATTGAGAACAAAGGAAAGAT
GTTTGACTCTCAGGTCATCATCATCACCACCAACCAACAGACACCCGTCC
CCTTGGATTATGTCAACCTTGAGGCCGTTTGCCGGCGCATCGATTTCCTG
GTCTATGCTGAGAGCCCCGTGGTCGATGCTGCGCGAGCCAGGTCCCCTGG
TGATGTGGCCGCTGTGAAAGCCGCCATGAGGCCCGATTACAGCCACATCA
ACTTCATTTTGGCCCCCCAGGGCGGCTTTGACCGCCAGGGCAACACACCC
TATGGCAAGGGGGTCACGAAGATCATCGGGGCCACTGCCCTCTGCGCCAG
GGCTGTTGCGCTTGTGCATGAGCGCCATGATGATTTCGGTCTGCAGAACA
AAGTCTATGATTTTGATGCTGGCAAGGTTACCGCATTCAAGACCATGGCT
GCTGATGCGGGCATCCCCTGGTATAAGATGGCGGCCATTGGCTACAAGGC
GATGGGCTGCACTTGTGTGGAGGAAGCCATGAACCTGCTCAAGGACTATG
AGGTAGCCCCCTGCCAGGTCATCTACAACGGTGCCACCTACAACGTCAGC
TGCATCAGGGGGGCCCCCATGGTTGAGAAAGTCAAGGAGCCTGAACTCCC
AAAAACACTGGTGAACTGCGTCAGGCGGATTAAGGAGGCCCGGCTCCGCT
GTTATTGCAGGATGGCCACAGACGTCATCACCTCCATCCTGCAGGCGGCT
GGGACAGCCTTCTCTATCTACCACCA
>304_M8
GCCTCCACCAACTCGGCCCTCAAGGCCGCGCAGGAGATGGGGAAGTTTGC
GATTGAGGTTTTCAAGCAAATCATGGCCTGGATCTGGCCGTCCGAGGACC
CGGTACCCGCCCTGCTCTCAAACATGGAGCAGGCTGTCATCAAGAACGAG
TGCCAGCTCGAGAACCAACTCACCACCATGTTGAGGGACCGAAATGCAGG
AGCAGAGTTCTTGAAGGCTCTAGATGAAGAAGAGCAGGAGGTTCGCAAGA
TTGCGGCCAAGTGTGGCAATTCTGCCACCACCGGCACGACTAATGCTCTC
TTGGCTAGGATCAGCATGGCCCGCGCTGCCTTTGAGAAGGCACGAGCCGA
GCAGACCTCCCGCGTCCGCCCAGTTGTGATTATGGTCTCTGGCAGGCCAG
GCATTGGGAAAACTTGCTTCTGCCAGAACCTAGCAAAAAGGATCGCGGCC
TCGCTGGGCGACGAGACCTCAGTTGGCATCATCCCGCGTGCGGACGTGGA
CCACTGGGACGCTTACAAGGGCGCCAGGGTGGTACTGTGGGACGACTTTG
GGATGGACAATGTAGTCAAGGATGCCCTGCGCCTGCAGATGCTCGCAGAC
ACATGCCCCGTCACTCTCAATTGTGACAGGATTGAGAACAAAGGAAAGAT
GTTTGACTCTCAGGTCATCATCATCACCACCAACCAGCAGACACCCGTCC
CCTTGGATTATGTCAACCTTGAGGCCGTTTGCCGGCGCATTGATTTCCTG
GTCTATGCTGAGAGCCCCGTGGTCGATGCTGCGCGAGCCAGGTCCCCTGG
TGATATAGCCGCTGTGAAAGCCGCCATGAGGCCCGATTACAGCCACATCA
ACTTCATTTTGGCCCCCCAGGGCGGCTTTGACCGCCAGGGCAACACACCC
TATGGTAAGGGGGTCACGAAGATCATCGGGGCCACTGCCCTCTGCGCCAG
GGCTGTTGCGCTTGTGCATGAGCGCCATGATGATTTCGGTCTGCAGAACA
AAGTCTATGATTTTGATGCTGGCAAGGTTACCGCATTCAAGACCATGGCT
GCTGATGCGGGCATCCCCTGGTATAAGATGGCGGCCATTGGCTACAAGGC
GATGGGCTGCACTTGCGTGGAGGAAGCCATGAACCTGCTCAAGGACTATG
AGGTAGCCCCCTGCCAGGTCATCTACAACGGTGCCACCTACAACGTCAGC
TGCATCAAGGGGGCCCCCATGGTTGAGAAAGTCAAGGAGCCTGAACTCCC
AAAAACACTGGTGAACTGCGTCAGGCGGATTAAGGAGGCCCGGCTCCGCT
GTTATTGCAGGATGGCCACAGACGTCATCACCTCCATCCTGCAGGCGGCT
GGGACAGCCTTCTCTATCTACCACCA
>304_Cae
GCCTCCACCAACTCGGCCCTCAAGGCCGCGCAGGAGATGGGGAAGTTTGC
GATTGAGGTTTTCAAGCAAATCATGGCCTGGATCTGGCCGTCCGAGGACC
CGGTACCCGCCCTGCTCTCAAACATGGAGCAGGCTGTCATCAAGAACGAG
TGCCAGCTCGAGAACCAACTCACCACCATGTTGAGGGACCGAAATGCAGG
AGCAGAGTTCTTGAAGGCTCTAGATGAAGAAGAGCAGGAGGTTCGCAAGA
TTGCGGCCAAGTGTGGCAATTCTGCCACCACCGGCACGACTAATGCTCTC
TTGGCTAGGATCAGCATGGCCCGCGCTGCCTTTGAGAAGGCACGAGCCGA

GCAGACTTCCCGCGTCCGCCCAGTTGTGATTATGGTCTCTGGCAGGCCAG
GCATTGGGAAAACTTGCTTCTGCCAGAACCTAGCAAAAAGGATCGCGGCC
TCGCTGGGCGACGAGACCTCAGTTGGCATCATCCCGCGTGCGGACGTGGA
CCACTGGGACGCTTACAAGGGCGCCAGGGTGGTACTGTGGGACGACTTTG
GGATGGACAATGTAGTCAAGGATGCCCTGCGCCTGCAGATGCTCGCAGAC
ACATGCCCCGTCACTCTCAATTGTGACAGGATTGAGAACAAAGGAAAGAT
GTTTGACTCTCAGGTCATCATCATCACCACCAACCAGCAGACACCCGTCC
CCTTGGATTATGTCAACCTTGAGGCCGTTTGCCGGCGCATTGATTTCCTG
GTTTATGCTGAGAGCCCCGTGGTCGATGCTGCGCGAGCCAGGTCCCCTGG
TGATACAGCCGCTGTGAAAGCCGCCATGAGGCCCGATTACAGCCACATCA
ACTTCATTTTGGCCCCCCAGGGCGGCTTTGACCGCCAGGGCAACACACCC
TATGGCAAGGGGGTCACGAAGATCATCGGGGCCACTGCCCTCTGCGCCAG
GGCTGTTGCGCTTGTGCATGAGCGCCATGATGATTTCGGTCTGCAGAACA
AAGTCTATGATTTTGATGCTGGCAAGGTTACCGCATTCAAGACCATGGCT
GCTGATGCGGGCATCCCCTGGTATAAGATGGCGGCCATTGGCTACAAGGC
GATGGGCTGCACTTGCGTGGAGGAAGCCATGAACCTGCTCAAGGACTATG
AGGTAGCCCCCTGCCAGGTCATCTACAACGGTGCCACCTACAACGTCAGC
TGCATCAAGGGGGCCCCCATGGTTGAGAAAGTCAAGGAGCCTGAACTCCC
AAAAACACTGGTGAACTGCGTCAGGCGGATTAAGGAGGCCCGGCTCCGCT
GTTATTGCAGGATGGCCACAGACGTCATCACCTCCATCCTGCAGGCGGCT
GGGACAGCCTTCTCTATCTACCACCA
>304_MLN
GCCTCCACCAACTCGGCCCTCAAGGCCGCGCAGGAGATGGGGAAGTTTGC
GATTGAGGTTTTCAAGCAAATCATGGCCTGGATCTGGCCGTCCGAGGATC
CGGTACCCGCCCTGCTCTCAAACATGGAGCAGGCTGTCATCAAGAACGAG
TGCCAGCTCGAGAACCAACTCACCACCATGTTGAGGGACCGAAATGCAGG
AGCAGAGTTCTTGAAGGCTCTAGATGAAGAAGAGCAGGAGGTTCGCAAGA
TTGCGGCCAAGTGTGGCAATTCTGCCACCACCGGCACGACTAATGCTCTC
TTGGCTAGGATCAGCATGGCCCGCGCTGCCTTTGAGAAGGCACGAGCCGA
GCAGACCTCCCGCGTCCGCCCAGTTGTGATTATGGTCTCTGGCAGGCCAG
GCATTGGGAAAACTTGCTTCTGCCAGAACCTAGCAAAAAGGATCGCGGCC
TCGCTGGGCGACGAGACCTCAGTTGGCATCATCCCGCGTGCGGACGTGGA
CCACTGGGACGCTTACAAGGGCGCCAGGGTGGTACTGTGGGACGACTTTG
GGATGGACAATGTAGTCAAGGATGCCCTGCGCCTGCAGATGCTCGCAGAC
ACATGCCCCGTCACTCTCAATTGTGACAGGATTGAGAACAAAGGAAAGAT
GTTTGACTCTCAGGTCATCATCATCACCACCAACCAGCAGACACCCGTCC
CCTTGGATTATGTCAACCTTGAGGCCGTTTGCCGGCGCATTGATTTCCTG
GTCTATGCTGAGAGCCCCGTGGTCGATGCTGCGCGAGCCATGTCCCCTGG
TGATGTAGCCGCTGTGAAAGCTGCCATGAGGCCCGATTACAGCCACATCA
ACTTCATTTTGGCCCCCCAGGGCGGCTTTGACCGCCAGGGCAACACACCC
TATGGCAAGGGGGTCACGAAGATCATCGGGGCCACTGCCCTCTGCGCCAG
GGCTGTTGCGCTTGTGCATGAGCGCCATGATGATTTCGGTCTGCAGAACA
AAGTCTATGATTTTGATGCTGGCAAGGTTACCGCATTCAAGACCATGGCT
GCTGATGCGGGCATCCCCTGGTATAAGATGGCGGCCATTGGCTACAAGGC
GATGGGCTGCACTTGCGTGGAGGAAGCCATGAACCTGCTCAAGGACTATG
AGGTAGCCCCCTGCCAGGTCATCTACAACGGTGCCACCTACAACGTCAGC
TGCATCAAGGGGGCCCCCATGGTTGAGAAAGTCAAGGAGCCTGAACTCCC
AAAAACACTGGTGAACTGCGTCAGGCGGATTAAGGAGGCCCGGCTCCGCT
GTTATTGCAGGATGGCCACAGACGTCATCACCTCCATCCTGCAGGCGGCT
GGGACAGCCTTCTCTATCTACCACCA
>305_Cae
NNNNNNNNCAACTCGGCCCTCAAGGCCGCGCAGGAGATGGGGAAGTTTGC
GATTGAGGTTTTCAAGCAAATCATGGCCTGGATCTGGCCGTCCGAGGATC
CGGTACCCGCCCTGCTCTCAAACATGGAGCAGGCTGTCATCAAGAACGAG
TGCCAGCTCGAGAACCAACTCACCACCATGTTGAGGGACCGAAATGCAGG
AGCAGAGTTCTTGAAGGCTCTAGATGAAGAAGAGCAGGAGGTTCGCAAGA
TTGCGGCCAAGTGTGGCAATTCTGCCACCACCGGCACGACTAATGCTCTC
TTGGCTAGGATCAGCATGGCCCGCGCTGCCTTTGAGAAGGCACGAGCCGA
GCAGACCTCCCGCGTCCGCCCAGTTGTGATTATGGTCTCTGGCAGGCCAG
GCATTGGGAAAACTTGCTTCTGCCAGAACCTAGCAAAAAGGATCGCGGCC
TCGCTGGGCGACGAGACCTCAGTTGGCATCATCCCGCGTGCGGACGTGGA

CCACTGGGACGCTTACAAGGGCGCCAGGGTGGTACTGTGGGACGACTTTG
GGATGGACAATGTAGTCAAGGATGCCCTGCGCCTGCAGATGCTCGCAGAC
ACATGCCCCGTCACTCTCAATTGTGACAGGATTGAGAACAAAGGAAAGAT
GTTTGACTCTCAGGTCATCATCATCACCACCAACCAGCAGACACCCGTCC
CCTTGGATTATGTCAACCTTGAGGCCGTTTGCCGGCGCATTGATTTCCTG
GTCTATGCTGAGAGCCCCGTGGTCGATGCTGCGCGAGCCAGGTCCCCTGG
TGATGTAGCCGCTGTGAAAGCTGCCATGAGGCCCGATTACAGCCACATCA
ACTTCATTTTGGCCCCCCAGGGCGGCTTTGACCGCCAGGGCAACACACCC
TATGGCAAGGGGGTCACGAAGATCATCGGGGCCACTGCCCTCTGCGCCAG
GGCTGTTGCGCTTGTGCATGAGCGCCATGATGATTTCGGTCTGCAGAACA
AAGTCTATGATTTTGATGCTGGCAAGGTTACCGCATTCAAGACCATGGCT
GCTGATGCGGGCATCCCCTGGTATAAGATGGCGGCCATTGGCTACAAGGC
GATGGGCTGCACTTGCGTGGAGGAAGCCATGAACCTGCTCAAGGACTATG
AGGTAGCCCCCTGCCAGGTCATCTACAACGGTGCCACCTACAACGTCAGC
TGCATCAAGGGGGCCCCCATGGTTGAGAAAGTCAAGGAGCCTGAACTCCC
AAAAACACTGGTGAACTGCGTCAGGCGGATTAAGGAGGCCCGGCTCCGCT
GTTATTGCAGGATGGCCACAGACGTCATCACCTCCATCCTGCAGGCGGCT
GGGACAGCCTTCTCTATCTACCACCA
>306_M5
GCCTCCACCAACTCGGCCCTCAAGGCCGCGCAGGAGATGGGGAAGTTTGC
GATTGAGGTTTTCAAGCAAATCATGGCCTGGATCTGGCCGTCCGAGGACC
CGGTACCCGCCCTGCTCTCAAACATGGAGCAGGCTGTCATCAAGAACGAG
TGCCAGCTCGAGAACCAACTCACCACCATGTTGAGGGACCGAAATGCAGG
AGCAGAGTTCTTGAAGGCTCTAGATGAAGAAGAGCAGGAGGTTCGCAAGA
TTGCGGCCAAGTGTGGCAATTCTGCCACCACCGGCACGACTAATGCTCTC
TTGGCTAGGATCAGCATGGCCCGCGCTGCCTTTGAGAAGGCACGAGCCGA
GCAGACCTCTCGCGTCCGCCCAGTTGTGATTATGGTCTCTGGCAGGCCAG
GCATTGGGAAAACTTGCTTCTGCCAGAACCTAGCAAAAAGGATCGCGGCC
TCGCTGGGCGACGAGACCTCAGTTGGCATCATCCCGCGTGCGGACGTGGA
CCACTGGGACGCTTACAAGGGCGCCAGGGTGGTACTGTGGGACGACTTTG
GGATGGACAATGTAGTCAAGGATGCCCTGCGCCTGCAGATGCTCGCAGAC
ACATGCCCCGTCACTCTTAATTGTGACAGGATTGAGAACAAAGGAAAGAT
GTTTGACTCTCAGGTCATCATCATCACCACCAACCAGCAGACACCCGTCC
CCTTGGATTATGTCAACCTTGAGGCCGTTTGCCGGCGCATCGATTTCCTG
GTCTATGCTGAGAGCCCCGTGGTCGACGCTGCGCGAGCCAGGTCCCCTGG
TGATGTGGCCGCTGTGAAAGCCGCCATGAGGCCCGATTACAGTCACATCA
ACTTCATTTTGGCCCCCCAGGGCGGCTTTGACCGCCAGGGCAACACACCC
TATGGCAAGGGGGTCACGAAGATCATCGGGGCCACTGCCCTCTGCGCCAG
GGCTGTTGCGCTTGTGCATGAGCGCCATGATGATTTCGGTCTGCAGAACA
AAGTCTATGATTTTGATGCTGGCAAGGTTACCGCATTCAAGACCATGGCT
GCTGATGCGGGCATCCCCTGGTATAAGATGGCGGCCATTGGCTACAAGGC
GATGGGCTGCACTTGCGTGGAGGAAGCCATGAACCTGCTCAAGGACTATG
AGGTAGCCCCCTGCCAGGTCATCTACAACGGTGCCACCTACAACGTCAGC
TGCATCAAGGGGGCCCCCATGGTTGAGAAAGTCAAGGAGCCTGAACTCCC
AAAAACACTGGTGAACTGCGTCAGGCGGATTAAGGAGGCCCGGCTCCGCT
GTTATTGCAGGATGGCCACAGACGTCATCACCTCCATCCTGCAGGCGGCT
GGGACAGCCTTCTCTATCTACCACCA
>306_M8
NNNNNNNNNNNNNNGGCCCTCAAGGCCGCGCAGGAGATGGGGAAGTTTGC
GATTGAGGTTTTCAAGCAAATCATGGCCTGGATCTGGCCGTCCGAGGACC
CGGTACCCGCCCTGCTCTCAAACATGGAGCAGGCTGTCATCAAGAACGAG
TGCCAGCTCGAGAACCAACTCACCACCATGTTGAGGGACCGAAATGCAGG
AGCAGAGTTCTTGAAGGCTCTAGATGAAGAAGAGCAGGAGGTTCGCAAGA
TTGCGGCCAAGTGTGGCAATTCTGCCACCATCGGCACGACTAATGCTCTC
TTGGCTAGGATCAGCATGGCCCGCGCTGCCTTTGAGAAGGCACGAGCCGA
GCAGACCTCCCGCGTCCGCCCAGTTGTGATTATGGTCTCTGGCAGGCCAG
GCATTGGGAAAACTTGCTTCTGCCAGAACCTAGCAAAAAGGATCGCGGCC
TCGCTGGGCGACGAGACCTCAGTTGGCATCATCCCGCGTGCGGACGTGGA
CCACTGGGACGCTTACAAGGGCGCCAGGGTGGTACTGTGGGACGACTTTG
GGATGGACAATGTAGTCAAGGATGCCCTGCGCCTGCAGATGCTCGCAGAC
ACATGCCCCGTCACTCTCAATTGTGACAGGATTGAGAACAAAGGAAAGAT

GTTTGACTCTCAGGTCATCATCATCACCACCAACCAGCAGACACCCGTCC
CCTTGGATTATGTCAACCTTGAGGCCGTTTGCCGGCGCATTGATTTCCTG
GTTTATGCTGAGAGCCCCGTGGTCGATGCTGCGCGAGCCAGGTCCCCTGG
TGATACAGCCGCTGTGAAAGCCGCCATGAGGCCCGATTACAGCCACATCA
ACTTCATTTTGGCCCCCCAGGGCGGCTTTGACCGCCAGGGCAACACACCC
TATGGCAAGGGGGTCACGAAGATCATCGGGGCCACTGCCCTCTGCGCCAG
GGCTGTTGCGCTTGTGCATGAGCGCCATGATGATTTCGGTCTGCAGAACA
AAGTCTATGATTTTGATGCTGGCAAGGTTACCGCATTCAAGACCATGGCT
GCTGATGCGGGCATCCCCTGGTATAAGATGGCGGCCATTGGCTACAAGGC
GATGGGCTGCACTTGCGTGGAGGAAGCCATGAACCTGCTCAAGGACTATG
AGGTAGCCCCCTGCCAGGTCATCTACAACGGTGCCACCTACAACGTCAGC
TGCATCAAGGGGGCCCCCATGGTTGAGAAAGTCAAGGAGCCTGAACTCCC
AAAAACACTGGTGAACTGCNNNNNNNNNNNNNNNNNNNNNNNNNNNNNNN
NNNNNNNNNNNNNNNNNNNNNNNNNNNNNNNNNNNNNNNNNNNNNNNNNN
NNNNNNNNNNNNNNNNNNNNNNNNNN
>306_Cae
GCCTCCACCAACTCGGCCCTCAAGGCCGCGCAGGAGATGGGGAAGTTTGC
GATTGAGGTTTTCAAGCAAATCATGGCCTGGATCTGGCCGTCCGAGGAYC
CGGTACCCGCCCTGCTCTCAAACATGGAGCAGGCTGTCATCAAGAACGAG
TGCCAGCTCGAGAACCAACTCACCACCATGTTGAGGGACCGAAATGCAGG
AGCAGAGTTCTTGAAGGCTCTAGATGAAGAAGAGCAGGAGGTTCGCAAGA
TTGCGGCCAAGTGTGGCAATTCTGCCACCACCGGCACGACTAATGCTCTC
TTGGCTAGGATCAGCATGGCCCGCGCTGCCTTTGAGAAGGCACGAGCCGA
GCAGACCTCCCGCGTCCGCCCAGTTGTGATTATGGTCTCTGGCAGGCCAG
GCATTGGGAAAACTTGCTTCTGCCAGAACCTAGCAAAAAGGATCGCGGCC
TCGCTGGGCGACGAGACCTCAGTTGGCATCATCCCGCGTGCGGACGTGGA
CCACTGGGACGCTTACAAGGGCGCCAGGGTGGTACTGTGGGACGACTTTG
GGATGGACAATGTAGTCAAGGATGCCCTGCGCCTGCAGATGCTCGCAGAC
ACATGCCCCGTCACTCTCAATTGTGACAGGATTGAGAACAAAGGAAAGAT
GTTTGACTCTCAGGTCATCATCATCACCACCAACCAGCAGACACCCGTCC
CCTTGGATTATGTCAACCTTGAGGCCGTTTGCCGGCGCATTGATTTCCTG
GTCTATGCTGAGAGCCCCGTGGTCGATGCTGCGCGAGCCAGGTCCCCTGG
TGATGTAGCCGCTGTGAAAGCTGCCATGAGGCCCGATTACAGCCACATCA
ACTTCATTTTGGCCCCCCAGGGCGGCTTTGACCGCCAGGGCAACACACCC
TATGGCAAGGGGGTCACGAAGATCATCGGGGCCACTGCCCTCTGCGCCAG
GGCTGTTGCGCTTGTGCATGAGCGCCATGATGATTTCGGTCTGCAGAACA
AAGTCTATGATTTTGATGCTGGCAAGGTTACCGCATTCAAGACCATGGCT
GCTGATGCGGGCATCCCCTGGTATAAGATGGCGGCCATTGGCTACAAGGC
GATGGGCTGCACTTGCGTGGAGGAAGCCATGAACCTGCTCAAGGACTATG
AGGTAGCCCCCTGCCAGGTCATCTACAACGGTGCCACCTACAACGTCAGC
TGCATCAAGGGGGCCCCCATGGTTGAGAAAGTCAAGGAGCCTGAACTCCC
AAAAACACTGGTGAACTGCGTCAGGCGGATTAAGGAGGCCCGGCTCCGCT
GTTATTGCAGGATGGCCACAGACGTCATCACCTCCATCCNNNNNNNNNNN
NNNNNNNNNNNNNNNNNNNNNNNNNN
>306_Colon
GCCTCCACCAACTCGGCCCTCAAGGCCGCGCAGGAGATGGGGAAGTTTGC
GATTGAGGTTTTCAAGCAAATCATGGCCTGGATCTGGCCGTCCGAGGATC
CGGTACCCGCCCTGCTCTCAAACATGGAGCAGGCTGTCATCAAGAACGAG
TGCCAGCTCGAGAACCAACTCACCACCATGTTGAGGGACCGAAATGCAGG
AGCAGAGTTCTTGAAGGCTCTAGATGAAGAAGAGCAGGAGGTTCGCAAGA
TTGCGGCCAAGTGTGGCAATTCTGCCACCACCGGCACGACTAATGCTCTC
TTGGCTAGGATCAGCATGGCCCGCGCTGCCTTTGAGAAGGCACGAGCCGA
GCAGACCTCCCGCGTCCGCCCAGTTGTGATTATGGTCTCTGGCAGGCCAG
GCATTGGGAAAACTTGCTTCTGCCAGAACCTAGCAAAAAGGATCGCGGCC
TCGCTGGGCGACGAGACCTCAGTTGGCATCATCCCGCGTGCGGACGTGGA
CCACTGGGACGCTTACAAGGGCGCCAGGGTGGTACTGTGGGACGACTTTG
GGATGGACAATGTAGTCAAGGATGCCCTGCGCCTGCAGATGCTCGCAGAC
ACATGCCCCGTCACTCTCAATTGTGACAGGATTGAGAACAAAGGAAAGAT
GTTTGACTCTCAGGTCATCATCATCACCACCAACCAGCAGACACCCGTCC
CCTTGGATTATGTCAACCTTGAGGCCGTTTGCCGGCGCATTGATTTCCTG
GTCTATGCTGAGAGCCCCGTGGTCGATGCTGCGCGAGCCAGGTCCCCTGG

TGATGTAGCCGCTGTGAAAGCTGCCATGAGGCCCGATTACAGCCACATCA
ACTTCATTTTGGCCCCCCAGGGCGGCTTTGACCGCCAGGGCAACACACCC
TATGGCAAGGGGGTCACGAAGATCATCGGGGCCACTGCCCTCTGCGCCAG
GGCTGTTGCGCTTGTGCATGAGCGCCATGATGATTTCGGTCTGCAGAACA
AAGTCTATGATTTTGATGCTGGCAAGGTTACCGCATTCAAGACCATGGCT
GCTGATGCGGGCATCCCCTGGTATAAGATGGCGGCCATTGGCTACAAGGC
GATGGGCTGCACTTGCGTGGAGGAAGCCATGAACCTGCTCAAGGACTATG
AGGTAGCCCCCTGCCAGGTCATCTACAACGGTGCCACCTACAACGTCAGC
TGCATCAAGGGGGCCCCCATGGTTGAGAAAGTCAAGGAGCCTGAACTCCC
AAAAACACTGGTGAACTGCGTCAGGCGGATTAAGGAGGCCCGGCTCCGCT
GTTATTGCAGGATGGCCACAGACGTCATCACCTCCATCCTGCAGGCGGCT
GGGACAGCCTTCTCTATCTACCACCA
>307_M5
GCCTCCACCAACTCGGCCCTCAAGGCCGCCCAGGAGATGGGCAAGTTTGC
GATTGAGGTTTTCAAACAAATCATGGCATGGATCTGGCCCTCTGAGGACC
CGGTGCCCGCTCTGCTTTCCAACATGGAGCAGGCTGTCATCAAGAACGAG
TGCCAGCTTGAGAACCAACTCACGACCATGTTGCGGGACCGCAACGCTGG
AGCTGAGTTCTTGAAGGCACTCGACGAAGAAGAGCAAGAGGTCCGGAAAA
TTGCAGCCAAGTGTGGAAACTCTGCCACCACGGGCACAACCAACGCCCTG
TTGGCCAGGATTAGCATGGCTCGCGCAGCCTTTGAGAAGGCTCGCGCTGA
GCAGACTTCCCGGGTTCGCCCCGTAGTGATTATGGTCTCTGGCAGGCCCG
GGATCGGGAAAACTTGTTTTTGCCAGAACCTAGCAAAGAGAATTGCTGCC
TCCCTCGGGGATGAGACCTCAGTCGGCATCATACCGCGCGCCGACGTGGA
CCACTGGGATGCCTACAAGGGCGCCAGGGTGGTCCTCTGGGATGATTTTG
GCATGGACAATGTGGTGAAGGATGCACTGCGGCTGCAGATGCTTGCTGAC
ACATGCCCCGTCACGCTCAACTGTGACAGGATTGAGAACAAGGGNAAGAT
GTTTGATTCTCAAGTTATCATCATCACCACGAACCAGCAGACCCCAGTGC
CTTTGGATTATGTCAACCTTGAGGCGGTCTGCCGCCGCATAGACTTCCTG
GTCTATGCCGAGAGCCCCGTGGTCGACGCTGCCCGAGCCAGGTCACCGGG
CGACGTGGCCGCTGTCAAGGCCGCTATGAGGCCAGACTACAGCCATATCA
ATTTCATCTTGGCACCGCAGGGTGGCTTTGACCGGCAGGGCAACACCCCC
TACGGCAAGGGCGTCACTAAGATCATCGGCGCCACCGCGCTCTGTGCAAG
GGCGGTCGCTCTTGTCCATGAGCGTCATGATGATTTTGGCCTCCAGAACA
AAGTCTATGATTTTGATGCTGGCAAGGTTACCGCCTTCAAGACCATGGCG
GCTGATGCCGGCATCCCCTGGTACAAGATGGCAGCGATTGGCTACAAGGC
CATGGGCTGCACCTGCGTGGAGGAAGCCATGAACCTACTCAAGGACTATG
AGGTGGCCCCGTGCCAGGTGATCTACAACGGAGCCACCTACAATGTCAGC
TGCATCAAGGGGGCGCCCATGGTTGAGAAAGTCAAGGAGCCTGAGCTGCC
CAAAACCCTAGTCAATTGTGTCAGGAGGATCAAGGAGGCCCGCGTCCGCT
GCTATTGCAGGATGGCCACAGATGTCATCACCTCCATCCTGCAGGCGGCT
GGGACAGCCTTCTCTATCTACCACCA
>307_M8
GCCTCCACCAACTCGGCCCTCAAGGCCGCCCAGGAGATGGGCAAGTTTGC
GATTGAGGTTTTCAAACAAATCATGGCATGGATCTGGCCCTCTGAGGACC
CGGTGCCCGCTCTGCTTTCCAACATGGAGCAGGCTGTCATCAAGAACGAG
TGCCAGCTTGAGAACCAACTCACGACCATGTTGCGGGACCGCAACGCTGG
AGCTGAGTTCTTGAAGGCACTCGACGAAGAAGAGCAAGAGGTCCGGAAAA
TTGCAGCCAAGTGTGGAAACTCTGCCACCACGGGCACAACCAACGCCCTG
TTGGCCAGGATTAGCATGGCTCGCGCAGCCTTTGAGAAGGCTCGCGCTGA
GCAGACTTCCCGGGTTCGCCCCGTAGTGATTATGGTCTCTGGCAGGCCCG
GGATCGGGAAAACTTGTTTTTGCCAGAACCTAGCAAAGAGAATTGCTGCC
TCCCTCGGGGATGAGACCTCAGTCGGCATCATACCGCGCGCCGACGTGGA
CCACTGGGATGCCTACAAGGGCGCCAGGGTGGTCCTCTGGGATGATTTTG
GCATGGACAATGTGGTGAAGGATGCACTGCGGCTGCAGATGCTTGCTGAC
ACATGCCCCGTCACGCTCAACTGTGACAGGATTGAGAACAAGGGGAAGAT
GTTTGATTCTCAAGTTATCATCATCACCACGAACCAGCAGACCCCAGTGC
CTTTGGATTATGTCAACCTTGAGGCGGTCTGCCGCCGCATAGACTTCCTG
GTCTATGCCGAGAGCCCCGTGGTCGACGCTGCCCGAGCCAGGTCACCGGG
CGACGTGGCCGCTGTCAAGGCCGCTATGAGGCCAGACTACAGCCATATCA
ATTTCATCTTGGCACCGCAGGGTGGCTTTGACCGGCAGGGCAACACCCCC
TACGGCAAGGGCGTCACTAAGATCATCGGCGCCACCGCGCTCTGTGCAAG

GGCGGTCGCTCTTGTCCATGAGCGTCATGATGATTTTGGCCTCCAGAACA
AAGTCTATGATTTTGATGCTGGCAAGGTTACCGCCTTCAAGACCATGGCG
GCTGATGCCGGCATCCCCTGGTACAAGATGGCAGCGATTGGCTACAAGGC
CATGGGCTGCACCTGCGTGGAGGAAGCCATGAACCTACTCAAGGACTATG
AGGTGGCCCCGTGCCAGGTGATCTACAACGGAGCCACCTACAATGTCAGC
TGCATCAAGGGGGCGCCCATGGTTGAGAAAGTCAAGGAGCCTGAGCTGCC
CAAAACCCTAGTCAATTGTGTCAGGAGGATCAAGGAGGCCCGCCTCCGCT
GCTATTGCAGGATGGCCACAGATGTCATCACCTCCATCCTGCAGGCGGCT
GGGACAGCCTTCTCTATCTACCACCA
>308_M5
GCCTCCACCAACTCGGCCCTCAAGGCCGCCCAGGAGATGGGCAAGTTTGC
GATTGAGGTTTTCAAACAAATCATGGCATGGATCTGGCCCTCTGAGGACC
CGGTGCCCGCTCTGCTTTCCAACATGGAGCAGGCTGTCATCAAGAACGAG
TGCCAGCTTGAGAACCAACTCACGACCATGTTGCGGGACCGCAACGCTGG
AGCTGAGTTCTTGAAGGCACTCGACGAAGAAGAGCAAGAGGTCCGGAAAA
TTGCAGCCAAGTGTGGAAACTCTGCCACCACGGGCACAACCAACGCCCTG
TTGGCCAGGATTAGCATGGCTCGCGCAGCCTTTGAGAAGGCTCGCGCTGA
GCAGACTTCCCGGGTTCGCCCCGTAGTGATTATGGTCTCTGGCAGGCCCG
GGATCGGGAAAACTTGTTTTTGCCAGAACCTAGCAAAGAGAATTGCTGCC
TCCCTCGGGGATGAGGCCTCAGTCGGCATCATACCGCGCGCCGACGTGGA
CCACTGGGATGCCTACAAGGGCGCCAGGGTGGTCCTCTGGGATGATTTTG
GCATGGACAATGTGGTGAAGGATGCACTGCGGCTGCAGATGCTTGCTGAC
ACATGCCCCGTCACGCTCAACTGTGACAGGATTGAGAACAAGGGGAAGAT
GTTTGATTCTCAAGTTATCATCATCACCACGAACCAGCAGACCCCAGTGC
CTTTGGATTATGTCAACCTTGAGGCGGTCTGCCGCCGCATAGACTTCCTG
GTCTATGCCGAGAGCCCCGTGGTCGACGCTGCCCGAGCCAGGTCACCGGG
CGACGTGGCCGCTGTCAAGGCCGCTATGAGGCCAGACTACAGCCATATCA
ATTTCATCTTGGCACCGCAGGGTGGCTTTGACCGGCAGGGCAACACCCCC
TACGGCAAGGGCGTCACTAAGATCATCGGCGCCACCGCGCTCTGTGCAAG
GGCGGTCGCTCTTGTCCATGAGCGTCATGATGATTTTGGCCTCCAGAACA
AAGTCTATGATTTTGATGCTGGCAAGGTTACCGCCTTCAAGACCATGGCG
GCTGATGCCGGCATCCCCTGGTACAAGATGGCAGCGATTGGCTACAAGGC
CATGGGCTGCACCTGCGTGGAGGAAGCCATGAACCTACTCAAGGACTATG
AGGTGGCCCCGTGCCAGGTGATCTACAACGGAGCCACCTACAATGTCAGC
TGCATCAAGGGGGCGCCCATGGTTGAGAAAGTCAAGGAGCCTGAGCTGCC
CAAAACCCTAGTCAATTGTGTCAGGAGGATCAAGGAGGCCCGCCTCCGCT
GCTATTGCAGGATGGCCACAGATGTCATCACCTCCATCCTGCAGGCGGCT
GGGACAGCCTTCTCTATCTACCACCA
>308_Colon
GCCTCCACCAACTCGGCCCTCAAGGCCGCCCAGGAGATGGGCAAGTTTGC
GATTGAGGTTTTCAAACAAATCATGGCATGGATCTGGCCCTCTGAGGACC
CGGTGCCCGCTCTGCTTTCCAACATGGAGCAGGCTGTCATCAAGAACGAG
TGCCAGCTTGAGAACCAACTCACGGCCATGTTGCGGGACCGCAACGCTGG
AGCTGAGTTCTTGAAGGCACTCGACGAAGAAGAGCAAGAGGTCCGGAAAA
TTGCAGCCAAGTGTGGAAACTCTGCCACCACGGGTACAACCAACGCCCTG
TTGGCCAGGATTAGCATGGCTCGCGCAGCCTTTGAGAAGGCTCGCGCTGA
GCAGACTTCCCGGGTTCGCCCCGTAGTGATTATGGTCTCTGGCAGGCCCG
GGATCGGGAAAACTTGTTTTTGCCAGAACCTAGCAAAGAGAATTGCTGCC
TCCCTCGGGGATGAGGCCTCAGTCGGCATCATACCGCGCGCCGACGTGGA
CCACTGGGATGCCTACAAGGGCGCCAGGGTGGTCCTCTGGGATGATTTTG
GCATGGACAATGTGGTGAAGGATGCACTGCGGCTACAGATGCTTGCTGAC
ACATGCCCCGTCACGCTCAACTGTGACAGGATTGAGAACAAGGGGAAGAT
GTTTGATTCTCAAGTTATCATCATCACCACGAACCAGCAGACCCCAGTGC
CTTTGGATTATGTCAACCTTGAGGCGGTCTGCCGCCGCATAGACTTCCTG
GTCTATGCCGAGAGCCCCGTGGTCGACGCTGCCCGAGCCAGGTCACCGGG
CGACGTGGCCGCTGTCAAGGCCGCTATGAGGCCAGACTACAGCCATATCA
ATTTCATCTTGGCACCGCAGGGTGGCTTTGACCGGCAGGGCAACACCCCC
TACGGCAAGGGCGTCACTAAGATCATCGGCGCCACCGCGCTCTGTGCAAG
GGCGGTCGCTCTTGTCCATGAGCGTCATGATGATTTTGGCCTCCAGAACA
AAGTCTATGATTTTGATGCTGGCAAGGTTACCGCCTTCAAGACCATGGCG
GCTGATGCCGGCATCCCCTGGTACAAGATGGCAGCGATTGGCTACAAGGC

CATGGGCTGCACCTGCGTGGAGGAAGCCATGAACCTACTCAAGGACTATG
AGGTGGCCCCGTGCCAGGTGATCTACAATGGAGCCACCTACAATGTCAGC
TGCATCAAGGGGGCGCCCATGGTTGAGAAAGTCAAGGAGCCTGAGCTGCC
CAAAACCCTAGTCAATTGTGTCAGGAGGATCAAGGAGGCCCGCCTCCGCT
GCTATTGTAGGATGGCCACAGATGTCATCACCTCCATCCTGCAGGCGGCT
GGGACAGCCTTCTCTATCTACCACCA
>308_MLN
GCCTCCACCAACTCGGCCCTCAAGGCCGCCCAGGAGATGGGCAAGTTTGC
GATTGAGGTTTTCAAACAAATCATGGCATGGATCTGGCCCTCTGAGGACC
CGGTGCCCGCTCTGCTTTCCAACATGGAGCAGGCTGTCATCAAGAACGAG
TGCCAGCTTGAGAACCAACTCACGGCCATGTTGCGGGACCGCAACGCTGG
AGCTGAGTTCTTGAAGGCACTCGACGAAGAAGAGCAAGAGGTCCGGAAAA
TTGCAGCCAAGTGTGGAAACTCTGCCACCACGGGTACAACCAACGCCCTG
TTGGCCAGGATTAGCATGGCTCGCGCAGCCTTTGAGAAGGCTCGCGCTGA
GCAGACTTCCCGGGTTCGCCCCGTAGTGATTATGGTCTCTGGCAGGCCCG
GGATCGGGAAAACTTGTTTTTGCCAGAACCTAGCAAAGAGAATTGCTGCC
TCCCTCGGGGATGAGGCCTCAGTCGGCATCATACCGCGCGCCGACGTGGA
CCACTGGGATGCCTACAAGGGCGCCAGGGTGGTCCTCTGGGATGATTTTG
GCATGGACAATGTGGTGAAGGATGCACTGCGGCTACAGATGCTTGCTGAC
ACATGCCCCGTCACGCTCAACTGTGACAGGATTGAGAACAAGGGGAAGAT
GTTTGATTCTCAAGTTATCATCATCACCACGAACCAGCAGACCCCAGTGC
CTTTGGATTATGTCAACCTTGAGGCGGTCTGCCGCCGCATAGACTTCCTG
GTCTATGCCGAGAGCCCCGTGGTCGACGCTGCCCGAGCCAGGTCACCGGG
CGACGTGGCCGCTGTCAAGGCCGCTATGAGGCCAGACTACAGCCATATCA
ATTTCATCTTGGCACCGCAGGGTGGCTTTGACCGGCAGGGCAACACCCCC
TACGGCAAGGGCGTCACTAAGATCATCGGCGCCACCGCGCTCTGTGCAAG
GGCGGTCGCTCTTGTCCATGAGCGTCATGATGATTTTGGCCTCCAGAACA
AAGTCTATGATTTTGATGCTGGCAAGGTTACCGCCTTCAAGACCATGGCG
GCTGATGCCGGCATCCCCTGGTACAAGATGGCAGCGATTGGCTACAAGGC
CATGGGCTGCACCTGCGTGGAGGAAGCCATGAACCTACTCAAGGACTATG
AGGTGGCCCCGTGCCAGGTGATCTACAATGGAGCCACCTACAATGTCAGC
TGCATCAAGGGGGCGCCCATGGTTGAGAAAGTCAAGGAGCCTGAGCTGCC
CAAAACCCTAGTCAATTGTGTCAGGAGGATCAAGGAGGCCCGCCTCCGCT
GCTATTGTAGGATGGCCACAGATGTCATCACCTCCATCCTGCAGGCGGCT
GGGACAGCCTTCTCTATCTACCACCA
>309_M5
GCCTCCACCAACTCGGCCCTCAAGGCCGCCCAGGAGATGGGCAAGTTTGC
GATTGAGGTTTTCAAACAAATCATGGCATGGATCTGGCCCTCTGAGGACC
CGGTGCCCGCTCTGCTTTCCAACATGGAGCAGGCTGTCATCAAGAACGAG
TGCCAGCTTGAGAATCAACTCACGGCCATGTTGCGGGACCGCAACGCTGG
AGCTGAGTTCTTGAAGGCACTCGACGAAGAAGAGCAAGAGGTCCGGAAAA
TAGCAGCCAAGTGTGGAAACTCTGCCACCACGGGCACAACCAACGCCCTG
TTGGCCAGGATTAGCATGGCTCGCGCAGCCTTTGAGAAGGCTCGCGCTGA
GCAGACTTCCCGGGTTCGCCCCGTAGTGATTATGGTCTCTGGCAGGCCCG
GGATCGGGAAAACTTGTTTTTGCCAGAACCTAGCAAAGAGAATTGCTGCC
TCCCTCGGGGATGAGACCTCAGTCGGCATCATACCGCGCGCCGACGTGGA
CCACTGGGATGCCTACAAGGGCGCCAGGGTGGTCCTCTGGGATGATTTTG
GCATGGACAATGTGGTGAAGGATGCACTGCGGCTGCAGATGCTTGCTGAC
ACATGCCCCGTCACGCTCAACTGTGACAGGATTGAGAACAAGGGGAAGAT
GTTTGATTCTCAAGTTATCATCATCACCACGAACCAGCAGACCCCAGTGC
CTTTGGATTATGTCAACCTTGAGGCGGTCTGCCGCCGCATAGACTTTCTG
GTCTATGCCGAGAGCCCCGTGGTCGACGCTGCCCGAGCCAGGTCACCGGG
CGACGTGGCCGCTGTCAAGGCCGCTATGAGGCCAGACTACAGCCATATCA
ATTTCATCTTGGCACCGCAGGGTGGCTTTGACCGGCAGGGCAACACCCCC
TACGGCAAGGGCGTCACTAAGATCATCGGCGCCACCGCGCTCTGTGCAAG
GGCGGTCGCTCTTGTCCATGAGCGTCATGATGATTTTGGCCTCCAGAACA
AAGTCTATGATTTTGATGCTGGCAAGGTTACCGCCTTCAAGACCATGGCG
GCTGATGCCGGCATCCCCTGGTACAAGATGGCAGCGATTGGCTACAAGGC
CATGGGCTGCACCTGCGTGGAGGAAGCCATGAACCTACTCAAGGACTATG
AGGTGGCCCCGTGCCAGGTGATCTACAACGGAGCCACCTACAATGTCAGC
TGCATCAAGGGGGCGCCCATGGTTGAGAAAGTCAAGGAGCCTGAGCTGCC

CAAAACCCTAGTCAATTGTGTCAGGAGGATCAAGGAGGCCCGCCTCCGCT
GCTATTGCAGGATGGCCACAGATGTCATCACCTCCATCCTGCAGGCGGCT
GGGACAGCCTTCTCTATCTACCACCA
>309_M8
GCCTCCACCAACTCGGCCCTCAAGGCCGCCCAGGAGATGGGCAAGTTTGC
GATTGAGGTTTTCAAACAAATCATGGCATGGATCTGGCCCTCTGAGGACC
CGGTGCCCGCTCTGCTTTCCAACATGGAGCAGGCTGTCATCAAGAACGAG
TGCCAGCTTGAGAATCAACTCACGGCCATGTTGCGGGACCGCAACGCTGG
AGCTGAGTTCTTGAAGGCACTCGACGAAGAAGAGCAAGAGGTCCGGAAAA
TTGCAGCCAAGTGTGGAAACTCTGCCACCACGGGCACAACCAACGCCCTG
TTGGCCAGGATTAGCATGGCTCGCGCAGCCTTTGAGAAGGCTCGCGCTGA
GCAGACTTCCCGGGTTCGCCCCGTAGTGATTATGGTCTCTGGCAGGCCCG
GGATCGGGAAAACTTGTTTTTGCCAGAACCTAGCAAAGAGAATTGCTGCC
TCCCTCGGGGATGAGACCTCAGTCGGCATCATACCGCGCGCCGACGTGGA
CCACTGGGATGCCTACAAGGGCGCCAGGGTGGTCCTCTGGGATGATTTTG
GCATGGACAATGTGGTGAAGGATGCACTGCGGCTGCAGATGCTTGCTGAC
ACATGCCCCGTCACGCTCAACTGTGACAGGATTGAGAACAAGGGGAAGAT
GTTTGATTCTCAAGTTATCATCATCACCACGAACCAGCAGACCCCAGTGC
CTTTGGATTATGTCAACCTTGAGGCGGTCTGCCGCCGCATAGACTTTCTG
GTCTATGCCGAGAGCCCCGTGGTCGACGCTGCCCGAGCCAGGTCACCGGG
CGACGTGGCCGCTGTCAAGGCCGCTATGAGGCCAGACTACAGCCATATCA
ATTTCATCTTGGCACCGCAGGGTGGCTTTGACCGGCAGGGCAACACCCCC
TACGGCAAGGGCGTCACTAAGATCATCGGCGCCACCGCGCTCTGTGCAAG
GNCGGTCGCTCTTGTCCATGAGCGTCATGATGATTTTGGCCTCCAGAACA
AAGTCTATGATTTTGATGCTGGCAAGGTTACCGCCTTCANGACCATGGCG
GCTGATGCCGGCATCCCCTGGTACAAGATGGCAGCGATTGGCTACAAGGC
CATGGGCTGCACCTGCGTGGAGGAAGCCATGAACCTACTCAAGGACTATG
AGGTGGCCCCGTGCCAGGTGATCTACAACGGAGCCACCTACAATGTCAGC
TGCATCAAGGGGGCGCCCATGGTTGAGAAAGTCAAGGAGCCTGAGCTGCC
CAAAACCCTAGTCAATTGTGTCAGGAGGATCAAGGAGGCCCGCCTCCGCT
GCTATTGCAGGATGGCCACAGATGTCATCACCTCCATCCTGCAGGCGGCT
GGGACAGCCTTCTCTATCTACCACCA
>309_Cae
GCCTCCACCAACTCGGCCCTCAAGGCCGCCCAGGAGATGGGCAAGTTTGC
GATTGAGGTTTTCAAACAAATCATGGCATGGATCTGGCCCTCTGAGGACC
CGGTGCCCGCTCTGCTTTCCAACATGGAGCAGGCTGTCATCAAGAACGAG
TGCCAGCTTGAGAATCAACTCACGGCCATGTTGCGGGACCGCAACGCTGG
AGCTGAGTTCTTGAAGGCACTCGACGAAGAAGAGCAAGAGGTCCGGAAAA
TTGCAGCCAAGTGTGGAAACTCTGCCACCACGGGCACAACCAACGCCCTG
TTGGCCAGGATTAGCATGGCTCGCGCAGCCTTTGAGAAGGCTCGCGCTGA
GCAGACTTCCCGGGTTCGCCCCGTAGTGATTATGGTCTCTGGCAGGCCCG
GGATCGGGAAAACTTGTTTTTGCCAGAACCTAGCAAAGAGAATTGCTGCC
TCCCTCGGGGATGAGACCTCAGTCGGCATCATACCGCGCGCCGACGTGGA
CCACTGGGATGCCTACAAGGGCGCCAGGGTGGTCCTCTGGGATGATTTTG
GCATGGACAATGTGGTGAAGGATGCACTGCGGCTGCAGATGCTTGCTGAC
ACATGCCCCGTCACGCTCAACTGTGACAGGATTGAGAACAAGGGGAAGAT
GTTTGATTCTCAAGTTATCATCATCACCACGAACCAGCAGACCCCAGTGC
CTTTGGATTATGTCAACCTTGAGGCGGTCTGCCGCCGCATAGACTTTCTG
GTCTATGCCGAGAGCCCCGTGGTCGACGCTGCCCGAGCCAGGTCACCGGG
CGACGTGGCCGCTGTCAAGGCCGCTATGAGGCCAGACTACAGCCATATCA
ATTTCATCTTGGCACCGCAGGGTGGCTTTGACCGGCAGGGCAACACCCCC
TACGGCAAGGGCGTCACTAAGATCATCGGCGCCACCGCGCTCTGTGCAAG
GGCGGTCGCTCTTGTCCATGAGCGTCATGATGATTTTGGCCTCCANAACA
AAGTCTATGATTTTGATGCTGGCAAGGTTACCGCCTTCAAGACCATGGCG
GCTGATGCCGGCATCCCCTGGTACAAGATGGCAGCGATTGGCTACAAGGC
CATGGGCTGCACCTGCGTGGAGGAAGCCATGAACCTACTCAAGGACTATG
AGGTGGCCCCGTGCCAGGTGATCTACAACGGAGCCACCTACAATGTCAGC
TGCATCAAGGGGGCGCCCATGGTTGAGAAAGTCAAGGAGCCTGAGCTGCC
CAAAACCCTAGTCAATTGTGTCAGGAGGATCAAGGAGGCCCGCCTCCGCT
GCTATTGCAGGATGGCCACAGATGTCATCACCTCCATCCTGCAGGCGGCT
GGGACAGCCTTCTCTATCTACCACCA

>309_Colon
GCCTCCACCAACTCGGCCCTCAAGGCCGCCCAGGAGATGGGCAAGTTTGC
GATTGAGGTTTTCAAACAAATCATGGCATGGATCTGGCCCTCTGAGGACC
CGGTGCCCGCTCTGCTTTCCAACATGGAGCAGGCTGTCATCAAGAACGAG
TGCCAGCTTGAGAACCAACTCACGACCATGTTGCGGGACCGCAACGCTGG
AGCTGAGTTCTTGAAGGCACTCGACGAAGAAGAGCAAGAGGTCCGGAAAA
TTGCAGCCAAGTGTGGAAACTCTGCCACCACGGGCACAACCAACGCCCTG
TTGGCCAGGATTAGCATGGCTCGCGCAGCCTTTGAGAAGGCTCGCGCTGA
GCAGACTTCCCGGGTTCGCCCCGTAGTGATTATGGTCTCTGGCAGGCCCG
GGATCGGGAAAACTTGTTTTTGCCAGAACCTAGCAAAGAGAATTGCTGCC
TCCCTCGGGGATGAGACCTCAGTCGGCATCATACCGCGCGCCGACGTGGA
CCACTGGGATGCCTACAAGGGCGCCAGGGTGGTCCTCTGGGATGATTTTG
GCATGGACAATGTGGTGAAGGATGCACTGCGGCTGCAGATGCTTGCTGAC
ACATGCCCCGTCACGCTCAACTGTGACAGGATTGAGAACAAGGGGAAGAT
GTTTGATTCTCAAGTTATCATCATCACCACGAACCAGCAGACCCCAGTGC
CTTTGGATTATGTCAACCTTGAGGCGGTCTGCCGCCGCATAGACTTCCTG
GTCTATGCCGAGAGCCCCGTGGTCGACGCTGCCCGAGCCAGGTCACCGGG
CGACGTGGCCGCTGTCAAGGCCGCTATGAGGCCAGACTACAGCCATATCA
ATTTCATCTTGGCACCGCAGGGTGGCTTTGACCGGCAGGGCAACACCCCC
TACGGCAAGGGCGTCACTAAGATCATCGGCGCCACCGCGCTCTGTGCAAG
GGCGGTCGCTCTTGTCCATGAGCGTCATGATGATTTTGGCCTCCAGAACA
AAGTCTATGATTTTGATGCTGGCAAGGTTACCGCCTTCAAGACCATGGCG
GCTGATGCCGGCATCCCCTGGTACAAGATGGCAGCGATTGGCTACAAGGC
CATGGGCTGCACCTGCGTGGAGGAAGCCATGAACCTACTCAAGGACTATG
AGGTGGCCCCGTGCCAGGTGATCTACAACGGAGCCACCTACAATGTCAGC
TGCATCAAGGGGGCGCCCATGGTTGAGAAAGTCAAGGAGCCTGAGCTGCC
CAAAACCCTAGTCAATTGTGTCAGGAGGATCAAGGAGGCCCGCCTCCGCT
GCTATTGCAGGATGGCCACAGATGTCATCACCTCCATCCTGCAGGCGGCT
GGGACAGCCTTCTCTATCTACCACCA
>310_M5
NNNNNNNNNNNNNNNNNNNTCAAGGCCGCCCAGGAGATGGGCAAGTTTGC
GATTGAGGTTTTCAAACAAATCATGGCATGGATCTGGCCCTCTGAGGACC
CGGTGCCCGCTCTGCTTTCCAACATGGAGCAGGCTGTCATCAAGAACGAG
TGCCAGCTTGAGAATCAACTCACGGCCATGTTGCGGGACCGCAACGCTGG
AGCTGAGTTCTTGAAGGCACTCGACGAAGAAGAGCAAGAGGTCCGGAAAA
TTGCAGCCAAGNGTGGAAACTCTGCCACCACGGGCACAACCAACGCCCTG
TTGGCCAGGATTAGCATGGCTCGCGCAGCCTTTGAGAAGGCTCGCGCTGA
GCAGACTTCCCGGGTTCGCCCCGTAGTGATTATGGTCTCTGGCAGGCCCG
GGATCGGGAAAACTTGTTTTTGCCAGAACCTAGCAAAGAGAATTGCTGCC
TCCCTCGGGGATGAGACCTCAGTCGGCATCATACCGCGCGCCGACGTGGA
CCACTGGGATGCCTACAAGGGCGCCAGGGTGGTCCTCTGGGATGATTTTG
GCATGGACAATGTGGTGAAGGATGCACTGCGGCTGCAGATGCTTGCTGAC
ACATGCCCCGTCACGCTCAACTGTGACAGGATTGAGAACAAGGGGAAGAT
GTTTGATTCTCAAGTTATCATCATCACCACGAACCAGCAGACCCCAGTGC
CTTTGGATTATGTCAACCTTGAGGCGGTCTGCCGCCGCATAGACTTTCTG
GTCTATGCCGAGAGCCCCGTGGTCGACGCTGCCCGAGCCAGGTCACCGGG
CGACGTGGCCGCTGTCAAGGCCGCTATGAGGCCAGACTACAGCCATATCA
ATTTCATCTTGGCACCGCAGGGTGGCTTTGACCGGCAGGGCAACACCCCC
TACGGCAAGGGCGTCACTAAGATCATCGGCGCCACCGCGCTCTGTGCAAG
GGCGGTCGCTCTTGTCCATGAGCGTCATGATGATTTTGGCCTCCAGAACA
AAGTCTATGATTTTGATGCTGGCAAGGTTACCGCCTTCAAGACCATGGCG
GCTGATGCCGGCATCCCCTGGTACAAGATGGCAGCGATTGGCTACAAGGC
CATGGGCTGCACCTGCGTGGAGGAAGCCATGAACCTACTCAAGGACTATG
AGGTGGCCCCGTGCCAGGTGATCTACAACGGAGCCACCTACAATGTCAGC
TGCATCAAGGGGGCGCCCATGGTTGAGAAAGTCAAGGAGCCTGAGCTGCC
CAAAACCCTAGTCAATTGTGTCAGGAGGATCAAGGAGGCCCGCCTCCGCT
GCTATTGCAGGATGGCCACAGATGTCATCACCTCCATCCTGCAGGCGGCT
GGGACAGCCTTCTCTATCTACCACCA
>310_M8
GCCTCCACCAACTCGGCCCTCAAGGCCGCCCAGGAGATGGGCAAGTTTGC
GATTGAGGTTTTCAAACAAATCATGGCATGGATCTGGCCCTCTGAGGACC

```
CGGTGCCCGCTCTGCTTTCCAACATGGAGCAGGCTGTCATCAAGAACGAG
TGCCAGCTTGAGAATCAACTCACGGCCATGTTGCGGGACCGCAACGCTGG
AGCTGAGTTCTTGAAGGCACTCGACGAAGAAGAGCAAGAGGTCCGGAAAA
TTGCAGCCAAGTGTGGAAACTCTGCCACCACGGGCACAACCAACGCCCTG
TTGGCCAGGATTAGCATGGCTCGCGCAGCCTTTGAGAAGGCTCGCGCTGA
GCAGACTTCCCGGGTTCGCCCCGTAGTGATTATGGTCTCTGGCAGGCCCG
GGATCGGGAAAACTTGTTTTTGCCAGAACCTAGCAAAGAGAATTGCTGCC
TCCCTCGGGGATGAGACCTCAGTCGGCATCATACCGCGCGCCGACGTGGA
CCACTGGGATGCCTACAAGGGCGCCAGGGTGGTCCTCTGGGATGATTTTG
GCATGGACAATGTGGTGAAGGATGCACTGCGGCTGCAGATGCTTGCTGAC
ACATGCCCCGTCACGCTCAACTGTGACAGGATTGAGAACAAGGGGAAGAT
GTTTGATTCTCAAGTTATCATCATCACCACGAACCAGCAGACCCCAGTGC
CTTTGGATTATGTCAACCTTGAGGCGGTCTGTCGCCGCATAGACTTTCTG
GTCTATGCCGAGAGCCCCGTGGTCGACGCTGCCCGAGCCAGGTCACCGGG
CGACGTGGCCGCTGTCAAGGCCGCTATGAGGCCAGACTACAGCCATATCA
ATTTCATCTTGGCACCGCAGGGTGGCTTTGACCGGCAGGGCAACACCCCC
TACGGCAAGGGCGTCACTAAGATCATCGGCGCCACCGCGCTCTGTGCAAG
GGCGGTCGCTCTTGTCCATGAGCGTCATGATGATTTTGGCCTCCAGAACA
AAGTCTATGATTTTGATGCTGGCAAGGTTACCGCCTTCAAGACCATGGCG
GCTGATGCCGGCATCCCCTGGTACAAGATGGCAGCGATTGGCTACAAGGC
CATGGGCTGCACCTGCGTGGAGGAAGCCATGAACCTACTCAAGGACTATG
AGGTGGCCCCGTGCCAGGTGATCTACAACGGAGCCACCTACAATGTCAGC
TGCATCAAGGGGGCGCCCATGGTTGAGAAAGTCAAGGAGCCTGAGCTGCC
CAAAACCCTAGTCAATTGTGTCAGGAGGATCAAGGAGGCCCGCCTCCGCT
GCTATTGCAGGATGGCCACAGATGTCATCACCTCCATCCTGCAGGCGGCT
GGGACAGCCTTCTCTATCTACCACCA
>310_Colon
GCCTCCACCAACTCGGCCCTCAAGGCCGCCCAGGAGATGGGCAAGTTTGC
GATTGAGGTTTTCAAACAAATCATGGCATGGATCTGGCCCTCTGAGGACC
CGGTGCCCGCTCTGCTTTCCAACATGGAGCAGGCTGTCATCAAGAACGAG
TGCCAGCTTGAGAACCAACTCACGACCATGTTGCGGGACCGCAACGCTGG
AGCTGAGTTCTTGAAGGCACTCGACGAAGAAGAGCAAGAGGTCCGGAAAA
TTGCAGCCAAGTGTGGAAACTCTGCCACCACGGGCACAACCAACGCCCTG
TTGGCCAGGATTAGCATGGCTCGCGCAGCCTTTGAGAAGGCTCGCGCTGA
GCAGACTTCCCGGGTTCGCCCCGTAGTGATTATGGTCTCTGGCAGGCCCG
GGATCGGGAAAACTTGTTTTTGCCAGAACCTAGCAAAGAGAATTGCTGCC
TCCCTCGGGGATGAGACCTCAGTCGGCATCATACCGCGCGCCGACGTGGA
CCACTGGGATGCCTACAAGGGCGCCAGGGTGGTCCTCTGGGATGATTTTG
GCATGGACAATGTGGTGAAGGATGCACTGCGGCTGCAGATGCTTGCTGAC
ACATGCCCCGTCACGCTCAACTGTGACAGGATTGAGAACAAGGGGAAGAT
GTTTGATTCTCAAGTTATCATCATCACCACGAACCAGCAGACCCCAGTGC
CTTTGGATTATGTCAACCTTGAGGCGGTCTGCCGCCGCATAGACTTCCTG
GTCTATGCCGAGAGCCCCGTGGTCGACGCTGCCCGAGCCAGGTCACCGGG
CGACGTGGCCGCTGTCAAGGCCGCTATGAGGCCAGACTACAGCCATATCA
ATTTCATCTTGGCACCGCAGGGTGGCTTTGACCGGCAGGGCAACACCCCC
TACGGCAAGGGCGTCACTAAGATCATCGGCGCCACCGCGCTCTGTGCAAG
GGCGGTCGCTCTTGTCCATGAGCGTCATGATGATTTTGGCCTCCAGAACA
AAGTCTATGATTTTGATGCTGGCAAGGTTACCGCCTTCAAGACCATGGCG
GCTGATGCCGGCATCCCCTGGTACAAGATGGCAGCGATTGGCTACAAGGC
CATGGGCTGCACCTGCGTGGAGGAAGCCATGAACCTACTCAAGGACTATG
AGGTGGCCCCGTGCCAGGTGATCTACAACGGAGCCACCTACAATGTCAGC
TGCATCAAGGGGGCGCCCATGGTTGAGAAAGTCAAGGAGCCTGAGCTGCC
CAAAACCCTAGTCAATTGTGTCAGGAGGATCAAGGAGGCCCGCCTCCGCT
GCTATTGCAGGATGGCCACAGATGTCATCACCTCCATCCTGCAGGCGGCT
GGGACAGCCTTCTCTATCTACCACCA
```

Region 2

```
>F1+2
TTCGGCACGGGATGGGGCTTTTGGGTTAGCGGCCATGTGTTCATCACCGC
TAAACATGTGGCGCCACCCAAGGGCACCGAGGTGTTCGGGCGCAAGCCCG
```

AGGAATTCACCGTCACCTCCAGTGGGGACTTCCTGAAATACCACTTCACC
AGTGCTGTGAGGCCTGACATTCCTGCCATGGTTCTTGAGAATGGCTGCCA
GGAGGGTGTTGTTGCTTCGGTCCTCGTCAAGAGGGCCTCCGGAGAGATGC
TCGCCCTGGCGGTGAGGATGGGTTCTCAGGCCGCCATCAAGATCGGCAAC
GCAGTGGTGCACGGCCAGACTGGGATGCTTCTCACAGNNTCCAATGCCAA
AGCCCAGGACCTCGGAACCATCCCCGGCGATTGCGGCTGCCCCTATGTCT
ACAAAAAGGGCAACACCTGGGTTGTGATTGGTGTGCACGTGGCGGCCACC
AGGTCGGGGAACACGGTGATCGCTGCTACCCATGGGGAACCCACCCTTGA
GGCGCTGGAGTTCCAAGGACCCCCCATGCTCCCTCGTCCCTCTGGCACCT
ATGTAGGCCTCCCCATTGCAGACTATGGTGATGCCCCTCCCCTCAGCACC
AAGACCATGTTCTGGCGCACTTCTCCAGAGAAGCTGCCGCCTGGGGCATG
GGAGCCCGCTTACCTGGGTTCCAAGGATGAGAGGGTGGACGGTCCCTCCC
TCCAGCAGGTTATGCGAGACCAGCTCAAGCCCTACTCCGAGCCTCGTGGA
TTACTCCCCCCGCAGGAGATTTTGGACGCAGTTTGTGATGCCATCGAGAA
CCGACTTGAGAATACGCTGGAGCCCCAGAAGCCCTGGACCTTTAAGAAAG
CCTGCGAGAGCCTTGATAAGAACACTAGCAGTGGCTATCCCTACCACAAA
CAGAAGAGTAGGGACTGGACGGGCACCGCCTTCATTGGTGAGCTCGGTGA
TCAAGCCACCCATGCCAACAAC
>MNV1
TTCGGCACGGGGTGGGGCTTTTGGGTGAGCGGCCACGTCTTCATCACCGC
CAAGCATGTGGCGCCCCCCAAGGGCACGGAGATCTTTGGGCGCAAGCCCG
GGGACTTCACTGTCACTTCCAGCGGGGACTTCTTGAAGTACTACTTCACC
AGCGCCGTCAGGCCTGACATTCCCGCCATGGTCCTGGAGAATGGGTGCCA
GGAGGGCGTCGTCGCCTCGGTCCTTGTCAAGAGAGCCTCCGGCGAGATGC
TTGCCCTGGCTGTCAGGATGGGTTCACAGGCCGCCATCAAGATTGGTAGT
GCCGTTGTGCATGGGCAAACTGGCATGCTCCTGACTGGCTCTAATGCCAA
GGCCCAGGACCTCGGGACCATCCCGGGCGACTGTGGCTGTCCCTATGTTT
ATAAGAAGGGTAACACCTGGGTTGTGATTGGGGTGCACGTGGCGGCCACT
AGGTCTGGTAACACAGTCATTGCCGCCACTCACGGAGAACCCACACTTGA
GGCTCTGGAGTTCCAGGGACCCCCCATGCTTCCCCGCCCCTCAGGCACCT
ATGCAGGCCTCCCCATCGCCGATTACGGCGACGCTCCCCCCTTGAGCACC
AAGACCATGTTCTGGCGTACCTCGCCAGAGAAGCTTCCCCCTGGGGCTTG
GGAGCCAGCCTATCTCGGCTCTAAAGATGAGAGGGTGGACGGTCCTTCCC
TTCAGCAGGTCATGCGAGATCAGCTTAAGCCCTATTCAGAACCACGCGGT
CTGCTTCCCCCTCAAGAAATCCTTGATGCAGTCTGCGACGCCATTGAGAA
CCGCCTTGAGAACACCCTTGAACCACAGAAGCCCTGGACATTTAAGAAGG
CTTGTGAGAGCTTGGACAAGAACACCAGTAGTGGGTATCCCTATCACAAG
CAGAAGAGCAAGGACTGGACGGGGAGCGCTTTTATTGGCGATCTTGGTGA
CCAGGCCACCCACGCCAACAAC
>pT7:MNV\3
TTCGGCACGGGATGGGGCTTTTGGGTTAGCGGCCATGTGTTCATCACCGC
TAAACATGTGGCGCCACCCAAGGGCACCGAGGTGTTCGGGCGCAAGCCCG
AGGAATTCACCGTCACCTCCAGTGGGGACTTCCTGAAATACCACTTCACC
AGTGCCGTCAGGCCTGACATTCCTGCCATGGTTCTTGAGAATGGCTGCCA
GGAGGGCGTTGTTGCCTCGGTCCTCGTTAAGAGGGCCTCTGGTGAGATGC
TCGCCCTGGCGGTCAGGATGGGCTCGCAGGCAGCCATCAAGATCGGCAAC
GCTGTGGTGCACGGACAGACTGGCATGCTTCTGACTGGTTCCAATGCCAA
AGCCCAAGACCTCGGGACCATCCCGGGCGATTGTGGTTGCCCCTATGTCT
ACAAGAAAGGAAACACCTGGGTTGTGATTGGGGTGCACGTGGCGGCCACT
AGATCGGGCAACACCGTGATCGCCGCCACTCATGGAGAGCCCACCCTTGA
GGCTCTGGAGTTCCAGGGACCTCCCATGCTCCCTCGCCCCTCCGGCACCT
ATGTAGGTCTCCCCATTGCCGACTACGGTGATGCCCCTCCCCTGAGTACT
AAGACCATGTTCTGGCGCACCTCGCCAGAGAAGCTCCCCCCCGGGGCTTG
GGAGCCAGCTTACCTGGGCTCCAAGGATGAGAGAGTGGACGGCCCCTCCC
TGCAACAGGTCATGCGGGACCAGCTCAAGCCCTACTCAGAGCCGCGTGGC
TTACTCCCCCCCCAGGAGATTTTGGATGCGGTTTGTGATGCCATCGAGAA
CCGCCTTGAGAACACCCTTGAACCGCAGAAGCCCTGGACATTTAAGAAGG
CCTGCGAGAGTCTGGATAAGAACACCAGCAGTGGGTATCCTTACCATAAG
CAAAAGAGCAGGGACTGGACAGGGACTGCCTTCATTGGTGAGCTCGGTGA
TCAAGCCACCCATGCCAACAAC
>307_M5F2
TTCGGCACGGGATGGGGCTTTTGGGTTAGCGGCCATGTGTTCATCACCGC
TAAGCATGTGGCGCCATCCAAGGGCACCGAGGTGTTCGGGCGCAAGCCCG
AGGAATTCACCGTCACCTCCAGTGGGGACTTCCTGAAATACCACTTCACC
AGTGCCGTCAGGCCTGACATTCCTGCCATGGTTCTTGAGAATGGCTGCCA
GGAGGGCGTTGTTGCCTCGGTCCTCGTTAAGAGGGCCTCTGGTGAGATGC
TCGCCCTGGCGGTCAGGATGGGCTCGCAGGCAGCCATCAAGATCGGCAAC
GCTGTGGTGCACGGACAGACTGGCATGCTTCTGACTGGTTCCAATGCCAA
AGCCCAAGACCTCGGGACCATCCCGGGCGATTGTGGTTGCCCCTATGTCT

ACAAGAAAGGAAACACCTGGGTTGTGATTGGGGTGCACGTGGCGGCCACT
AGATCGGGCAACACCGTGATCGCCGCCACTCATGGAGAGCCCACCCTTGA
GGCTCTGGAGTTCCAGGGACCTCCCATGCTCCCTCGCCCCTCCGGCACCT
ATGCAGGTCTCCCCATTGCCGACTACGGTGATGCCCCTCCCCTGAGTACT
AAGACCATGTTCTGGCGCACCTCGCCAGAGAAGCTCCCCCCCGGGGCTTG
GGAGCCAGCTTACCTGGGCTCCAAGGATGAGAGAGTGGACGGCCCCTCCC
TGCAACAGGTCATGCGGGACCAGCTCAAGCCCTACTCAGAGCCGCGTGGC
TTACTCCCCCCCCAGGAGATTTTGGATGCGGTTTGTGATGCCATCGAGAA
CCGCCTTGAGAACACCCTTGAACCGCAGAAGCCCTGGACATTTAAGAAGG
CCTGCGAGAGTCTAGATAAGAACACCAGCAGTGGGTATCCTTACCATAAG
CAAAAGAGCAAGGACTGGACAGGGGCTGCCTTCATTGGTGAGCTCGGTGA
TCAAGCCACCCATGCCAACAAC
>307_M7F2
TTCGGCACGGGATGGGGCTTTTGGGTTAGCGGCCATGTGTTCATCACCGC
TAAACATGTGGCGCCATCCAAGGGCACCGAGGTGTTCGGGCGCAAGCCCG
AGGAATTCACCGTCACCTCCAGTGGGGACTTCCTGAAATACCACTTCACC
AGTGCCGTCAGGCCTGACATTCCTGCCATGGTTCTTGAGAATGGCTGCCA
GGAGGGCGTTGTTGCCTCGGTCCTCGTTAAGAGGGCCTCTGGTGAGATGC
TCGCCCTGGCGGTCAGGATGGGCTCGCAGGCAGCCATCAAGATCGGCAAC
GCTGTGGTGCACGGACAGACTGGCATGCTTCTGACTGGTTCCAATGCCAA
AGCCCAAGACCTCGGGACCATCCCGGGCGATTGTGGTTGCCCCTATGTCT
ACAAGAAAGGAAACACCTGGGTTGTGATTGGGGTGCACGTGGCGGCCACT
AGATCGGGCAACACCGTGATCGCCGCCACTCATGGAGAGCCCACCCTTGA
GGCTCTGGAGTTCCAGGGACCTCCCATGCTCCCTCGCCCCTCCGGCACCT
ATGCAGGTCTCCCCATTGCCGACTACGGTGATGCCCCTCCCCTGAGTACT
AAGACCATGTTCTGGCGCACCTCGCCAGAGAAGCTCCCCCCCGGGGCTTG
GGAGCCAGCTTACCTGGGCTCCAAGGATGAGAGAGTGGACGGCCCCTCCC
TGCAACAGGTCATGCGGGACCAGCTCAAGCCCTACTCAGAGCCGCGTGGC
TTACTCCCCCCCCAGGAGATTTTGGATGCGGTTTGTGATGCCATCGAGAA
CCGCCTTGAGAACACCCTTGAACCGCAGAAGCCCTGGACATTTAAGAAGG
CCTGCGAGAGTCTAGATAAGAACACCAGCAGTGGGTATCCTTACCATAAG
CAAAAGAGCAAGGACTGGACAGGGGCTGCCTTCATTGGTGAGCTCGGTGA
TCAAGCCACCCATGCCAACAAC
>307_M8F2
TTCGGCACGGGATGGGGCTTTTGGGTTAGCGGCCATGTGTTCATCACCGC
TAAACATGTGGCGCCATCCAAGGGCACCGAGGTGTTCGGGCGCAAGCCCG
AGGAATTCACCGTCACCTCCAGTGGGGACTTCCTGAAATACCACTTCACC
AGTGCCGTCAGGCCTGACATTCCTGCCATGGTTCTTGAGAATGGCTGCCA
GGAGGGCGTTGTTGCCTCGGTCCTCGTTAAGAGGGCCTCTGGTGAGATGC
TCGCCCTGGCGGTCAGGATGGGCTCGCAGGCAGCCATCAAGATCGGCAAC
GCTGTGGTGCACGGACAGACTGGCATGCTTCTGACTGGTTCCAATGCCAA
AGCCCAAGACCTCGGGACCATCCCGGGCGATTGTGGTTGCCCCTATGTCT
ACAAGAAAGGAAACACCTGGGTTGTGATTGGGGTGCACGTGGCGGCCACT
AGATCGGGCAACACCGTGATCGCCGCCACTCATGGAGAGCCCACCCTTGA
GGCTCTGGAGTTCCAGGGACCTCCCATGCTCCCTCGCCCCTCCGGCACCT
ATGCAGGTCTCCCCATTGCCGACTACGGTGATGCCCCTCCCCTGAGTACT
AAGACCATGTTCTGGCGCACCTCGCCAGAGAAGCTCCCCCCCGGGGCTTG
GGAGCCAGCTTACCTGGGCTCCAAGGATGAGAGAGTGGACGGCCCCTCCC
TGCAACAGGTCATGCGGGACCAGCTCAAGCCCTACTCAGAGCCGCGTGGC
TTACTCCCCCCCCAGGAGATTTTGGATGCGGTTTGTGATGCCATCGAGAA
CCGCCTTGAGAACACCCTTGAACCGCAGAAGCCCTGGACATTTAAGAAGG
CCTGCGAGAGTCTAGATAAGAACACCAGCAGTGGGTATCCTTACCATAAG
CAAAAGAGCAAGGACTGGACAGGGGCTGCCTTCATTGGTGAGCTCGGTGA
TCAAGCCACCCATGCCAACAAC
>307_CaeF2
TTCGGCACGGGATGGGGCTTTTGGGTTAGCGGCCATGTGTTCATCACCGC
TAAACATGTGGCGCCATCCAAGGGCACCGAGGTGTTCGGGCGCAAGCCCG
AGGAATTCACCGTCACCTCCAGTGGGGACTTCCTGAAATACCACTTCACC
AGTGCCGTCAGGCCTGACATTCCTGCCATGGTTCTTGAGAATGGCTGCCA
GGAGGGCGTTGTTGCCTCGGTCCTCGTTAAGAGGGCCTCTGGTGAGATGC
TCGCCCTGGCGGTCAGGATGGGCTCGCAGGCAGCCATCAAGATCGGCAAC
GCTGTGGTGCACGGACAGACTGGCATGCTTCTGACTGGTTCCAATGCCAA
AGCCCAAGACCTCGGGACCATCCCGGGTGATTGTGGTTGCCCCTATGTCT
ACAAGAAAGGAAACACCTGGGTTGTGATTGGGGTGCACGTGGCGGCCACT
AGATCGGGCAACACCGTGATCGCCGCCACTCATGGAGAGCCCACYCTTGA
GGCTCTGGAGTTCCAGGGACCTCCCATGCTCCCTCGCCCCTCCGGCACCT
ATGCAGGTCTCCCCATTGCCGACTACGGTGATGCCCCTCCCCTGAGTACT
AAGACCATGTTCTGGCGCACCTCGCCAGAGAAGCTCCCCCCCGGGGCTTG
GGAGCCAGCTTACCTGGGCTCCAAGGATGAGAGAGTGGACGGCCCCTCCC

TGCAACAGGTCATGCGGGACCAGCTCAAGCCCTACTCAGAGCCGCGTGGC
TTACTCCCCCCCCAGGAGATTTTGGATGCGGTTTGTGATGCCATCGAGAA
CCGCCTTGAGAACACCCTTGAACCGCAGAAGCCCTGGACATTTAAGAAGG
CCTGCGAGAGTCTAGATAAGAACACCAGCAGTGGGTATCCTTACCATAAG
CAAAAGAGCAAGGACTGGACAGGGGCTGCCTTCATTGGTGAGCTCGGTGA
TCAAGCCACCCATGCCAACAAC
>307_ColF2
TTCGGCACGGGATGGGGCTTTTGGGTTAGCGGCCATGTGTTCATCACCGC
TAAACATGTGGCGCCATCCAAGGGCACCGAGGTGTTCGGGCGCAAGCCCG
AGGAATTCACCGTCACCTCCAGTGGGGACTTCCTGAAATACCACTTCACC
AGTGCCGTCAGGCCTGACATTCCTGCCATGGTTCTTGAGAATGGCTGCCA
GGAGGGCGTTGTTGCCTCGGTCCTCGTTAAGAGGGCCTCTGGTGAGATGC
TCGCCCTGGCGGTCAGGATGGGCTCGCAGGCAGCCATCAAGATCGGCAAC
GCTGTGGTGCACGGACAGACTGGCATGCTTCTGACTGGTTCCAATGCCAA
AGCCCAAGACCTCGGGACCATCCCGGGTGATTGTGGTTGCCCCTATGTCT
ACAAGAAAGGAAACACCTGGGTTGTGATTGGGGTGCACGTGGCGGCCACT
AGATCGGGCAACACCGTGATCGCCGCCACTCATGGAGAGCCCACCCTTGA
GGCTCTGGAGTTCCAGGGACCTCCCATGCTCCCTCGCCCCTCCGGCACCT
ATGCAGGTCTCCCCATTGCCGACTACGGTGATGCCCCTCCCCTGAGTACT
AAGACCATGTTCTGGCGCACCTCGCCAGAGAAGCTCCCCCCCGGGGCTTG
GGAGCCAGCTTACCTGGGCTCCAAGGATGAGAGAGTGGACGGCCCCTCCC
TGCAACAGGTCATGCGGGACCAGCTCAAGCCCTACTCAGAGCCGCGTGGC
TTACTCCCCCCCCAGGAGATTTTGGATGCGGTTTGTGATGCCATCGAGAA
CCGCCTTGAGAACACCCTTGAACCGCAGAAGCCCTGGACATTTAAGAAGG
CCTGCGAGAGTCTAGATAAGAACACCAGCAGTGGGTATCCTTACCATAAG
CAAAAGAGCAAGGACTGGACAGGGGCTGCCTTCATTGGTGAGCTCGGTGA
TCAAGCCACCCATGCCAACAAC
>308_M5F2
TTCGGCACGGGATGGGGCTTTTGGGTTAGCGGCCATGTGTTCATCACCGC
TAAACATGTGGCGCCATCCAAGGGCACCGAGGTGTTCGGGCGCAAGCCCG
AGGAATTCACCGTCACCTCCAGTGGGGACTTCCTGAAATACCACTTCACC
AGTGCCGTCAGGCCTGACATTCCTGCCATGGTTCTTGAGAATGGCTGCCA
GGAGGGCGTTGTTGCCTCGGTCCTCGTTAAGAGGGCCTCTGGTGAGATGC
TCGCCCTGGCGGTCAGGATGGGCTCGCAGGCAGCCATCAAGATCGGCAAC
GCTGTGGTGCACGGACAGACTGGCATGCTTCTGACTGGTTCCAATGCCAA
AGCCCAAGACCTCGGGACCATCCCGGGCGATTGTGGTTGCCCCTATGTCT
ACAAGAAAGGAAACACCTGGGTTGTGATTGGGGTGCACGTGGCGGCCACT
AGATCGGGCAACACCGTGATCGCCGCCACTCATGGAGAGCCCACCCTTGA
GGCTCTGGAGTTCCAGGGACCTCCCATGCTCCCTCGCCCCTCCGGCACCT
ATGCAGGTCTCCCCATTGCCGACTACGGTGATGCCCCTCCCCTGAGTACT
AAGACCATGTTCTGGCGCACCTCGCCAGAGAAGCTCCCCCCCGGGGCTTG
GGAGCCAGCTTACCTGGGCTCCAAGGATGAGAGAGTGGACGGCCCCTCCC
TGCAACAGGTCATGCGGGACCAGCTCAAGCCCTACTCAGAGCCGCGTGGC
TTACTCCCCCCCCAGGAGATTTTGGATGCGGTTTGTGATGCCATCGAGAA
CCGCCTTGAGAACACCCTTGAACCGCAGAAGCCCTGGACATTTAAGAAGG
CCTGCGAGAGTCTGGATAAGAACACCAGCAGTGGGTATCCTTACCATAAG
CAAAAGAGCAAGGACTGGACAGGGGCTGCCTTCATTGGTGAGCTCGGTGA
TCAGGCCACCCATGCCAACAAC
>308_M7F2
TTCGGCACGGGATGGGGCTTTTGGGTTAGCGGCCATGTGTTCATCACCGC
TAAACATGTGGCGCCATCCAAGGGCACCGAGGTGTTCGGGCGCAAGCCCG
AGGAATTCACCGTCACCTCCAGTGGGGACTTCCTGAAATACCACTTCACC
AGTGCCGTCAGGCCTGACATTCCTGCCATGGTTCTTGAGAATGGCTGCCA
GGAGGGCGTTGTTGCCTCGGTCCTCGTTAAGAGGGCCTCTGGTGAGATGC
TCGCCCTGGCGGTCAGGATGGGCTCGCAGGCAGCCATCAAGATCGGCAAC
GCTGTGGTGCACGGACAGACTGGCATGCTTCTGACTGGTTCCAATGCCAA
AGCCCAAGACCTTGGGACCATCCCGGGCGATTGTGGTTGCCCCTATGTCT
ACAAGAAAGGAAACACCTGGGTTGTGATTGGGGTGCACGTGGCGGCCACT
AGATCGGGCAACACCGTGATCGCCGCCACTCATGGAGAGCCTACCCTTGA
GGCTCTGGAGTTCCAGGGACCTCCCATGCTCCCTCGCCCCTCCGGCACCT
ATGCAGGTCTCCCCATTGCCGACTACGGTGATGCCCCTCCCCTGAGTACT
AAGACCATGTTCTGGCGCACCTCGCCAGAGAAGCTCCCCCCCGGGGCTTG
GGAGCCAGCTTACCTAGGCTCCAAGGATGAGAGAGTGGACGGCCCCTCCC
TGCAACAGGTCATGCGGGACCAGCTCAAGCCCTACTCAGAGCCGCGTGGC
TTACTCCCCCCCCAGGAGATTTTGGATGCGGTTTGTGATGCCATCGAGAA
CCGCCTTGAGAACACCCTTGAACCGCAGAAGCCCTGGACATTTAAGAAGG
CCTGCGAGAGTCTGGATAAGAACACCAGCAGTGGGTATCCTTACCATAAG
CAAAAGAGCAAGGACTGGACAGGGGCTGCCTTCATTGGTGAGCTCGGTGA
TCAGGCCACCCATGCCAACAAC

>308_ColF2
TTCGGCACGGGATGGGGCTTTTGGGTTAGCGGCCATGTGTTCATCACCGC
TAAACATGTGGCGCCATCCAAGGGCACCGAGGTGTTCGGGCGCAAGCCCG
AGGAATTCACCGTCACCTCCAGTGGGGACTTCCTGAAATACCACTTCACC
AGTGCCGTCAGGCCTGACATTCCTGCCATGGTTCTTGAGAATGGCTGCCA
GGAGGGCGTTGTTGCCTCGGTCCTCGTTAAGAGGGCCTCTGGTGAGATGC
TCGCCCTGGCGGTCAGGATGGGCTCGCAGGCAGCCATCAAGATCGGCAAC
GCTGTGGTGCACGGACAGACTGGCATGCTTCTGACTGGTTCCAATGCCAA
AGCCCAAGACCTTGGGACCATCCCGGGCGATTGTGGTTGCCCCTATGTCT
ACAAGAAAGGAAACACCTGGGTTGTGATTGGGGTGCACGTGGCGGCCACT
AGATCGGGCAACACCGTGATCGCCGCCACTCATGGAGAGCCTACCCTTGA
GGCTCTGGAGTTCCAGGGACCTCCCATGCTCCCTCGCCCCTCCGGCACCT
ATGCAGGTCTCCCCATTGCCGACTACGGTGATGCCCCTCCCCTGAGTACT
AAGACCATGTTCTGGCGCACCTCGCCAGAGAAGCTCCCCCCCGGGGCTTG
GGAGCCAGCTTACCTAGGCTCCAAGGATGAGAGAGTGGACGGCCCCTCCC
TGCAACAGGTCATGCGGGACCAGCTCAAGCCCTACTCAGAGCCGCGTGGC
TTACTCCCCCCCCAGGAGATTTTGGATGCGGTTTGTGATGCCATCGAGAA
CCGCCTTGAGAACACCCTTGAACCGCAGAAGCCCTGGACATTTAAGAAGG
CCTGCGAGAGTCTGGATAAGAACACCAGCAGTGGGTATCCTTACCATAAG
CAAAAGAGCAAGGACTGGACAGGGGCTGCCTTCATTGGTGAGCTCGGTGA
TCAGGCCACCCATGCCAACAAC
>308_MLNF2
TTCGGCACGGGATGGGGCTTTTGGGTTAGCGGCCATGTGTTCATCACCGC
TAAACATGTGGCGCCATCCAAGGGCACCGAGGTGTTCGGGCGCAAGCCCG
AGGAATTCACCGTCACCTCCAGTGGGGACTTCCTGAAATACYACTTCACC
AGTGCCGTCAGGCCTGACATTCCTGCCATGGTTCTTGAGAATGGCTGCCA
GGAGGGCGTTGTTGCCTCGGTCCTCGTTAAGAGGGCCTCTGGTGAGATGC
TCGCCCTGGCGGTCAGGATGGGCTCGCAGGCAGCCATCAAGATCGGCAAC
GCTGTGGTGCACGGACAGACTGGCATGCTTCTGACTGGTTCCAATGCCAA
AGCCCAAGACCTTGGGACCATCCCGGGCGATTGTGGTTGCCCCTATGTCT
ACAAGAAAGGAAACACCTGGGTTGTGATTGGGGTGCACGTGGCGGCCACT
AGATCGGGCAACACCGTGATCGCCGCCACTCATGGAGAGCCTACCCTTGA
GGCTCTGGAGTTCCAGGGACCTCCCATGCTCCCTCGCCCCTCCGGCACCT
ATGCAGGTCTCCCCATTGCCGACTACGGTGATGCCCCTCCCCTGAGTACT
AAGACCATGTTCTGGCGCACCTCGCCAGAGAAGCTCCCCCCCGGGGCTTG
GGAGCCAGCTTACCTAGGCTCCAAGGATGAGAGAGTGGACGGCCCCTCCC
TGCAACAGGTCATGCGGGACCAGCTCAAGCCCTACTCAGAGCCGCGTGGC
TTACTCCCCCCCCAGGAGATTTTGGATGCGGTTTGTGATGCCATCGAGAA
CCGCCTTGAGAACACCCTTGAACCGCAGAAGCCCTGGACATTTAAGAAGG
CCTGCGAGAGTCTGGATAAGAACACCAGCAGTGGGTATCCTTACCATAAG
CAAAAGAGCARGGACTGGACAGGGGCTGCCTTCATTGGTGAGCTCGGTGA
TCAGGCCACCCATGCCAACAAC
>309_M7F2
TTCGGCACGGGATGGGGCTTTTGGGTTAGCGGCCATGTGTTCATCACCGC
TAAACATGTGGCGCCATCCAAGGGCACCGAGGTGTTCGGGCGCAAGCCCG
AGGAATTCACCGTCACCTCCAGTGGGGACTTCCTGAAATACCACTTCACC
AGTGCCGTCAGGCCTGACATTCCTGCCATGGTTCTTGAGAATGGCTGCCA
GGAGGGCGTTGTTGCCTCGGTCCTCGTTAAGAGGGCCTCTGGTGAGATGC
TCGCCCTGGCGGTCAGGATGGGCTCGCAGGCAGCCATCAAGATCGGCAAC
GCTGTGGTGCACGGACAGACTGGCATGCTTCTGACTGGTTCCAATGCCAA
AGCCCAAGACCTCGGGACCATCCCGGGCGATTGTGGTTGCCCCTATGTCT
ACAAGAAAGGAAACACCTGGGTTGTGATTGGGGTGCACGTGGCGGCCACT
AGATCGGGCAACACCGTGATCGCCGCCACTCATGGAGAGCCCACCCTTGA
GGCTCTGGAGTTCCAGGGACCTCCCATGCTCCCTCGCCCCTCCGGCACCT
ATGCAGGTCTCCCCATTGCCGACTACGGTGATGCCCCTCCCCTGAGTACT
AAGACCATGTTCTGGCGCACCTCGCCAGAGAAGCTCCCCCCCGGGGCTTG
GGAGCCAGCTTACCTGGGCTCCAAGGATGAGAGAGTGGACGGCCCCTCCC
TGCAACAGGTCATGCGGGACCAGCTCAAGCCCTACTCAGAGCCGCGTGGC
TTACTCCCCCCCCAGGAGATTTTGGATGCGGTTTGTGATGCCATCGAGAA
CCGCCTTGAGAACACCCTTGAACCGCAGAAGCCCTGGACATTTAAGAAGG
CCTGCGAGAGTCTAGATAAGAACACCAGCAGTGGGTATCCTTACCATAAG
CAAAAGAGCAAGGACTGGACAGGGGCTGCCTTCATTGGTGAGCTCGGTGA
TCAAGCCACCCATGCCAACAAC
>309_CaeF2
TTCGGCACGGGATGGGGCTTTTGGGTTAGCGGCCATGTGTTCATCACCGC
TAAACATGTGGCGCCACCCAAGGGCACCGAGGTGTTCGGGCGCAAGCCCG
AGGAATTCACCGTCACCTCCAGTGGGGACTTCCTGAAATACTACTTCACC
AGTGCCGTCAGGCCTGACATTCCTGCCATGGTTCTTGAGAATGGCTGCCA
GGAGGGCGTTGTTGCCTCGGTCCTCGTTAAGAGGGCCTCTGGTGAGATGC

TCGCCCTGGCGGTCAGGATGGGCTCGCAGGCAGCCATCAAGATCGGCAAC
GCTGTGGTGCACGGACAGACTGGCATGCTTCTGACTGGTTCCAATGCCAA
AGCCCAAGACCTCGGGACCATCCCGGGCGATTGTGGTTGCCCCTATGTCT
ACAAGAAAGGGAACACCTGGGTTGTGATTGGGGTGCACGTGGCGGCCACT
AGATCGGGCAACACCGTGATCGCCGCCACTCATGGAGAGCCCACCCTTGA
GGCTCTGGAGTTCCAGGGACCTCCCATGCTCCCTCGCCCCTCCGGCACCT
ATGCAGGTCTCCCCATTGCCGACTACGGTGATGCCCCTCCCCTGAGTACT
AAGACCATGTTCTGGCGCACCTCGCCAGAGAAGCTCCCCCCCGGGGCTTG
GGAGCCAGCTTACCTGGGCTCCAAGGATGAGAGGGTGGACGGCCCCTCCC
TGCAACAGGTCATGCGGGACCAGCTCAAGCCCTACTCAGAGCCGCGTGGC
TTACTCCCCCCCCAGGAGATTTTGGATGCGGTTTGTGATGCCATCGAGAA
CCGCCTTGAGAACACCCTTGAACCGCAGAAGCCCTGGACATTTAAGAAGG
CCTGCGAGAGTCTGGATAAGAACACCAGCAGTGGGTATCCTTACCATAAG
CAAAAGAGCAGGGACTGGACAGGGGCTGCCTTCATTGGTGAGCTCGGTGA
TCAAGCCACCCATGCCAACAAC
>309_ColF2
TTCGGCACGGGATGGGGCTTTTGGGTTAGCGGCCATGTGTTCATCACCGY
TAAACATGTGGCGCCACCCAAGGGCACCGAGGTGTTCGGGCGCAAGCCCG
AGGAATTCACCGTCACCTCCAGTGGGGACTTCCTGAAATACTACTTCACC
AGTGCCGTCAGGCCTGACATTCCTGCCATGGTTCTTGAGAATGGCTGCCA
GGAGGGCGTTGTTGCCTCGGTCCTCGTTAAGAGGGCCTCTGGTGAGATGC
TCGCCCTGGCGGTCAGGATGGGCTCGCAGGCAGCCATCAAGATCGGCAAC
GCTGTGGTGCACGGACAGACTGGCATGCTTCTGACTGGTTCCAATGCCAA
AGCCCAAGACCTCGGGACCATCCCGGGCGATTGTGGTTGCCCCTATGTCT
ACAAGAAAGGGAACACCTGGGTTGTGATTGGGGTGCACGTGGCGGCCACT
AGATCGGGCAACACCGTGATCGCCGCCACTCATGGAGAGCCCACCCTTGA
GGCTCTGGAGTTCCAGGGACCTCCCATGCTCCCTCGCCCCTCCGGCACCT
ATGCAGGTCTCCCCATTGCCGACTACGGTGATGCCCCTCCCCTGAGTACT
AAGACCATGTTCTGGCGCACCTCGCCAGAGAAGCTCCCCCCCGGGGCTTG
GGAGCCAGCTTACCTGGGCTCCAAGGATGAGAGGGTGGACGGCCCCTCCC
TGCAACAGGTCATGCGGGACCAGCTCAAGCCCTACTCAGAGCCGCGTGGC
TTACTCCCCCCCCAGGAGATTTTGGATGCGGTTTGTGATGCCATCGAGAA
CCGCCTTGAGAACACCCTTGAACCGCAGAAGCCCTGGACATTTAAGAAGG
CCTGCGAGAGTCTGGATAAGAACACCAGCAGTGGGTATCCTTACCATAAG
CAAAAGAGCAGGGACTGGACAGGGGCTGCCTTCATTGGTGAGCTCGGTGA
TCAAGCCACCCATGCCAACAAC
>310_M5F2
TTCGGCACGGGATGGGGCTTTTGGGTTAGCGGCCATGTGTTCATCACCGC
TAAACATGTGGCGCCACCCAAGGGCACCGAGGTGTTCGGGCGCAAGCCCG
AGGAATTCACCGTCACCTCCAGTGGGGACTTCCTGAAATACTACTTCACC
AGTGCCGTCAGGCCTGACATTCCTGCCATGGTTCTTGAGAATGGCTGCCA
GGAGGGCGTTGTTGCCTCGGTCCTCGTTAAGAGGGCCTCTGGTGAGATGC
TCGCCCTGGCGGTCAGGATGGGCTCGCAGGCAGCCATCAAGATCGGCAAC
GCTGTGGTGCACGGACAGACTGGCATGCTTCTGACTGGTTCCAATGCCAA
AGCCCAAGACCTCGGGACCATCCCGGGCGATTGTGGTTGCCCCTATGTCT
ACAAGAAAGGGAACACCTGGGTTGTGATTGGGGTGCACGTGGCGGCCACT
AGATCGGGCAACACCGTGATCGCCGCCACTCATGGAGAGCCCACCCTTGA
GGCTCTGGAGTTCCAGGGACCTCCCATGCTCCCTCGCCCCTCCGGCACCT
ATGCAGGTCTCCCCATTGCCGACTACGGTGATGCCCCTCCCCTGAGTACT
AAGACCATGTTCTGGCGCACCTCGCCAGAGAAGCTCCCCCCCGGGGCTTG
GGAGCCAGCTTACCTGGGCTCCAAGGATGAGAGGGTGGACGGCCCCTCCC
TGCAACAGGTCATGCGGGACCAGCTCAAGCCCTACTCAGAGCCGCGTGGC
TTACTCCCCCCCCAGGAGATTTTGGATGCGGTTTGTGATGCCATCGAGAA
CCGCCTTGAGAACACCCTTGAACCGCAGAAGCCCTGGACATTTAAGAAGG
CCTGCGAGAGTCTGGATAAGAACACCAGCAGTGGGTATCCTTACCATAAG
CAAAAGAGCAGGGACTGGACAGGGGCTGCCTTCATTGGTGAGCTCGGTGA
TCAAGCCACCCATGCCAACAAC
>310_M7F2
TTCGGCACGGGATGGGGCTTTTGGGTTAGCGGCCATGTGTTCATCACCGC
TAAACATGTGGCGCCACCCAAGGGCACCGAGGTGTTCGGGCGCAAGCCCG
AGGAATTCACCGTCACCTCCAGTGGGGACTTCCTGAAATACTACTTCACC
AGTGCCGTCAGGCCTGACATTCCTGCCATGGTTCTTGAGAATGGCTGCCA
GGAGGGCGTTGTTGCCTCGGTCCTCGTTAAGAGGGCCTCTGGTGAGATGC
TCGCCCTGGCGGTCAGGATGGGCTCGCAGGCAGCCATCAAGATCGGCAAC
GCTGTGGTGCACGGACAGACTGGCATGCTTCTGACTGGTTCCAATGCCAA
AGCCCAAGACCTCGGGACCATCCCGGGCGATTGTGGTTGCCCCTATGTCT
ACAAGAAAGGGAACACCTGGGTTGTGATTGGGGTGCACGTGGCGGCCACT
AGATCGGGCAACACCGTGATCGCCGCCACTCATGGAGAGCCCACCCTTGA
GGCTCTGGAGTTCCAGGGACCTCCCATGCTCCCTCGCCCCTCCGGCACCT

ATGCAGGTCTCCCCATTGCCGACTACGGTGATGCCCCTCCCCTGAGTACT
AAGACCATGTTCTGGCGCACCTCGCCAGAGAAGCTCCCCCCCGGGGCTTG
GGAGCCAGCTTACCTGGGCTCCAAGGATGAGAGGGTGGACGGCCCCTCCC
TGCAACAGGTCATGCGGGACCAGCTCAAGCCCTACTCAGAGCCGCGTGGC
TTACTCCCCCCCCAGGAGATTTTGGATGCGGTTTGTGATGCCATCGAGAA
CCGCCTTGAGAACACCCTTGAACCGCAGAAGCCCTGGACATTTAAGAAGG
CCTGCGAGAGTCTGGAYAAGAACACCAGCAGTGGGTATCCTTACCATAAG
CAAAAGAGCAGGGACTGGACAGGGGCTGCCTTCATTGGTGAGCTCGGTGA
TCAAGCCACCCATGCCAACAAC
>Hsu
TTCGGCACGGGATGGGGCTTTTGGGTTAGCGGCCATGTGTTCATCACCGC
TAAACATGTGGCGCCACCCAAGGGCACCGAGGTGTTCGGGCGCAAGCCCG
AGGAATTCACCGTCACCTCCAGTGGGGACTTCCTGAAATACCACTTCACC
AGTGCCGTCAGGCCTGACATCCCTGCCATGGTTCTTGAGAATGGCTGCCA
GGAGGGCGTTGTTGCCTCGGTCCTCGTTAAGAGGGCCTCTGGTGAGATGC
TCGCCCTGGCGGTCAGGATGGGCTCGCAGGCAGCCATCAAGATCGGCAAC
GCTGTGGTGCACGGACAGACTGGCATGCTTCTGACTGGTTCCAATGCCAA
AGCCCAAGACCTCGGGACCATTCCGGGCGATTGTGGTTGCCCCTATGTCT
ACAAGAAAGGAAACACCTGGGTTGTGATTGGGGTGCACGTGGCGGCCACT
AGATCGGGCAACACCGTGATCGCCGCCACTCATGGGGAGCCCACCCTTGA
GGCTCTGGAGTTCCAGGGACCTCCCATGCTTCCTCGCCCCTCCGGCACCT
ATGCAGGTCTCCCCATTGCCGACTACGGTGATGCCCCTCCCCTGAGTACT
AAGACCATGTTCTGGCGCACCTCGCCAGAGAAGCTCCCTCCCGGGGCTTG
GGAGCCAGCTTACCTGGGCTCCAAGGATGAGAGAGTGGACGGCCCCTCCC
TGCAACAGGTCATGCGGGACCAGCTCAAGCCCTACTCAGAGCCGCGTGGC
TTACTCCCCCCCCAGGAGATTTTGGATGCGGTTTGTGATGCCATCGAGAA
CCGCCTTGAGAACACCCTTGAACCGCAGAAGCCCTGGACATTTAAGAAGG
CCTGCGAGAGTCTGGATAAGAACACCAGCAGTGGGTATCCTTACCATAAG
CAAAAGAGCAGGGACTGGACAGGGACTGCCTTCATTGGTGAGCTCGGTGA
TCAAGCCACCCATGCCAACAAC
>302_M7F2
NNNGGCACGGGATGGGGCTTTTGGGTTAGCGGCCATGTGTTCATCACCGC
TAAACATGTGGCGCCACCCAAGGGCACCGAGGTGTTCGGGCGCAAGCCCG
AGGAATTCACCGTCACCTCCAGYGGGGACTTCCTGAAATACCACTTCACC
AGTGCTGTGAGGCCTGACATTCCTGCCATGGTTCTTGAGAATGGCTGCCA
GGAGGGTGTTGTTGCTTCGGTCCTCGTCAAGAGGGCCTCCGGAGAGATGC
TCGCCCTGGCGGTGAGGATGGGTTCTCAGGCCGCCATCAAGATCGGCAAC
GCAGTGGTGCACGGCCAGACTGGGATGCTTCTCACAGGCTCCAATGCCAA
AGCCCAGGACCTCGGAACCATCCCCGGCGATTGCGGCTGCCCCTATGTCT
ACAAAAAGGGCAACACCTGGGTTGTGATTGGTGTGCACGTGGCGGCCACC
AGGTCGGGGAACACGGTGATCGCTGCTACCCATGGGGAACCCACCCTTGA
GGCGCTGGAGTTCCAAGGACCCCCCATGCTCCCTCGTCCCTCTGGCACCT
ATGTAGGCCTCCCCATTGCAGACTATGGTGATGCCCCTCCCCTCAGCACC
AAGACCATGTTCTGGCGCACTTCTCCAGAGAAGCTGCCGCCTGGGGCATG
GGAGCCCGCTTACCTGGGTTCCAAGGATGAGAGGGTGGACGGTCCCTCCC
TCCAGCAGGTTATGCGAGACCAGCTCAAGCCCTACTCCGAGCCTCGTGGA
TTACTCCCCCCGCAGGAGATTTTGGACGCAGTTTGTGATGCCATCGAGAA
CCGACTTGAGAATACGCTGGAGCCCCAGAAGCCCTGGACCTTTAAGAAAG
CCTGCGAGAGCCTTGATAAGAACACTAGCAGTGGCTATCCCTACCACAAA
CAGAAGAGTAGGGACTGGACGGGCACCGCCTTCATTGGTGAGCTCGGTGA
TCAAGCCACCCATGCCAACAAC
>302_ColF2
TTCGGCACGGGATGGGGCTTTTGGGTTAGCGGCCATGTGTTCATCACCGC
TAAACATGTGGCGCCACCCAAGGGCACCGAGGTGTTCGGGCGCAAGCCCG
AGGAATTCACCGTCACCTCCAGTGGGGACTTCCTGAAATACCACTTCACC
AGTGCTGTGAGGCCTGACATTCCTGCCATGGTTCTTGAGAATGGCTGCCA
GGAGGGTATTGTTGCTTCGGTCCTCGTCAAGAGGGCCTCCGGAGAGATGC
TCGCCCTGGCGGTGAGGATGGGTTCCCAGGCCGCCATCAAGATCGGCAAC
GCAGTGGTGCACGGCCAGACTGGGATGCTTCTCACAGGCTCCAATGCCAA
AGCCCAGGACCTCGGAACCATCCCCGGCGATTGCGGCTGCCCCTATGTCT
ACAAAAAGGGCAACACCTGGGTTGTGATTGGTGTGCACGTGGCGGCCACC
AGGTCGGGGAACACGGTGATCGCTGCTACCCATGGGGAACCCACCCTTGA
GGCGCTGGAGTTCCAAGGACCCCCCATGCTCCCTCGTCCCTCTGGCACCT
ATGTAGGCCTCCCCATTGCAGACTATGGTGATGCCCCTCCCCTCAGCACC
AAGACCATGTTCTGGCGCACTTCTCCAGAGAAGCTGCCGCCTGGGGCATG
GGAGCCCGCTTACCTGGGTTCCAAGGATGAGAGGGTGGACGGTCCCTCCC
TCCAGCAGGTTATGCGAGACCAGCTCAAGCCCTACTCCGAGCCTCGTGGA
CTACTCCCCCCGCAGGAGATTTTGGATGCAGTTTGTGATGCCATCGAGAA
CCGACTTGAGAATACGCTGGAGCCCCAGAAGCCCTGGACCTTTAAGAAAG

CCTGCGAGAGCCTTGATAAGAACACTAGCAGTGGCTATCCCTACCACAAA
CAGAAGAGTAGGGACTGGACGGGCACCGCCTTCATTGGTGAGCTCGGTGA
TCAAGCCACCCATGCCAACAAC
>302_MLF2
TTCGGCACGGGATGGGGCTTTTGGGTTAGCGGCCATGTGTTCATCACCGC
TAAACATGTGGCGCCACCCAAGGGCACCGAGGTGTTCGGGCGCAAGCCCG
AGGAATTCACCGTCACCTCCAGTGGGGACTTCCTGAAATACCACTTCACC
AGTGCTGTGAGGCCTGACATTCCTGCCATGGTTCTTGAGAATGGCTGCCA
GGAGGGTGTTGTTGCTTCGGTCCTCGTCAAGAGGGCCTCCGGAGAGATGC
TCGCCCTGGCGGTGAGGATGGGTTCTCAGGCCGCCATCAAGATCGGCAAC
GCAGTGGTGCACGGCCAGACTGGGATGCTTCTCACAGGCTCCAATGCCAA
AGCCCAGGACCTCGGAACCATCCCCGGCGATTGCGGCTGCCCCTATGTCT
ACAAAAAGGGCAACACCTGGGTTGTGATTGGTGTGCACGTGGCGGCCACC
AGGTCGGGGAACACGGTGATCGCTGCTACCCATGGGGAACCCACCCTTGA
GGCGCTGGAGTTCCAAGGACCCCCCATGCTCCCTCGTCCCTCTGGCACCT
ATGTAGGCCTCCCCATTGCAGACTATGGTGATGCCCCTCCCCTCAGCACC
AAGACCATGTTCTGGCGCACTTCTCCAGAGAAGCTGCCGCCTGGGGCATG
GGAGCCCGCTTACCTGGGTTCCAAGGATGAGAGGGTGGACGGTCCCTCCC
TCCAGCAGGTTATGCGAGACCAGCTCAAGCCCTACTCCGAGCCTCGTGGA
CTACTCCCCCCGCAGGAGATTTTGGATGCAGTTTGTGATGCCATCGAGAA
CCGACTTGAGAATACGCTGGAGCCCCAGAAGCCCTGGACCTTTAAGAAAG
CCTGCGAGAGCCTTGATAAGAACACTAGCAGTGGCTATCCCTACCACAAA
CAGAAGAGTAGGGACTGGACGGGCACCGCCTTCATTGGTGAGCTCGGTGA
TCAAGCCACCCATGCCAACAAC
>303_M7F2
TTCGGCACGGGATGGGGCTTTTGGGTTAGCGGCCATGTGTTCATCACCGC
TAAACATGTGGCGCCACCCAAGGGCACCGAGGTGTTCGGGCGCAAGCCCG
AGGAATTCACCGTCACCTCCAGTGGGGACTTCCTGAAATACCACTTCACC
AGTGCTGTGAGGCCTGACATTCCTGCCATGGTTCTTGAGAATGGCTGCCA
GGAGGGTGTTGTTGCTTCGGTCCTCGTCAAGAGGGCCTCCGGAGAGATGC
TCGCCCTGGCGGTGAGGATGGGTTCTCAGGCCGCCATCAAGATCGGCAAC
GCAGTGGTGCACGGCCAGACTGGGATGCTTCTCACAGGCTCCAATGCCAA
AGCCCAGGACCTCGGAACCATCCCCGGCGATTGCGGCTGCCCCTATGTCT
ACAAAAAGGGCAACACCTGGGTTGTGATTGGTGTGCACGTGGCGGCCACC
AGGTCGGGGAACACGGTGATCGCTGCTACCCATGGGGAACCCACCCTTGA
GGCGCTGGAGTTCCAAGGACCCCCCATGCTCCCTCGTCCCTCTGGCACCT
ATGTAGGCCTCCCCATTGCAGACTATGGTGATGCCCCTCCCCTCAGCACC
AAGACCATGTTCTGGCGCACTTCTCCAGAGAAGCTGCCGCCTGGGGCATG
GGAGCCCGCTTACCTGGGTTCCAAGGATGAGAGGGTGGATGGTCCCTCCC
TCCAGCAGGTTATGCGAGACCAGCTCAAGCCCTACTCCGAGCCTCGTGGA
YTACTCCCCCCGCAGGAGATTTTGGACGCAGTTTGTGATGCCATCGAGAA
CCGACTTGAGAATACGCTGGAGCCCCAGAAGCCCTGGACCTTTAAGAAAG
CCTGCGAGAGCCTTGATAAGAACACTAGCAGTGGCTATCCCTACCACAAA
CAGAAGAGTAGGGACTGGACGGGCRCCGCCTTCATTGGTGAGCTCGGTGA
TCAAGCCACCCATGCCAACAAC
>303_M8F2
TTCGGCACGGGATGGGGCTTTTGGGTTAGCGGCCATGTGTTCATCACCGC
TAAACATGTGGCGCCACCCAAGGGCACCGAGGTGTTCGGGCGCAAGCCCG
AGGAATTCACCGTCACCTCCAGTGGGGACTTCCTGAAATACCACTTCACC
AGTGCTGTGAGGCCTGACATTCCTGCCATGGTTCTTGAGAATGGCTGCCA
GGAGGGTGTTGTTGCTTCGGTCCTCGTCAAGAGGGCCTCCGGAGAGATGC
TCGCCCTGGCGGTGAGGATGGGTTCTCAGGCCGCCATCAAGATCGGCAAC
GCAGTGGTGCACGGCCAGACTGGGATGCTTCTCACAGGCTCCAATGCCAA
AGCCCAGGACCTCGGAACCATCCCCGGCGATTGCGGCTGCCCCTATGTCT
ACAAAAAGGGCAACACCTGGGTTGTGATTGGTGTGCACGTGGCGGCCACC
AGGTCGGGGAACACGGTGATCGCTGCTACCCATGGGGAACCCACCCTTGA
GGCGCTGGAGTTCCAAGGACCCCCCATGCTCCCTCGTCCCTCTGGCACCT
ATGTAGGCCTCCCCATTGCAGAYTATGGTGATGCCCCTCCCCTCAGCACC
AAGACCATGTTCTGGCGCACTTCTCCAGAGAAGCTGCCGCCTGGGGCATG
GGAGCCCGCTTACCTGGGTTCCAAGGATGAGAGGGTGGACGGTCCCTCCC
TCCAGCAGGTTATGCGAGACCAGCTCAAGCCCTACTCCGAGCCTCGTGGA
CTACTCCCCCCGCAGGAGATTTTGGATGCAGTTTGTGATGCCATCGAGAA
CCGACTTGAGAATACGCTGGAGCCCCAGAAGCCCTGGACCTTTAAGAAAG
CCTGCGAGAGCCTTGATAAGAACACTAGCAGTGGCTATCCCTACCACAAA
CAGAAGAGTAGGGACTGGACGGGCACCGCCTTCATTGGTGAGCTCGGTGA
TCAAGCCACCCATGCCAACAAC
>303_CaeF2
TTCGGCACGGGATGGGGCTTTTGGGTTAGCGGCCATGTGTTCATCACCGC
TAAACATGTGGCGCCACCCAAGGGCACCGAGGTGTTCGGGCGCAAGCCCG

AGGAATTCACCGTCACCTCCAGTGGGGACTTCCTGAAATACCACTTCACC
AGTGCTGTGAGGCCTGACATTCCTGCCATGGTTCTTGAGAATGGCTGCCA
GGAGGGTGTTGTTGCTTCGGTCCTCGTCAAGAGGGCCTCCGGAGAGATGC
TCGCCCTGGCGGTGAGGATGGGTTCTCAGGCCGCCATCAAGATCGGCAAC
GCAGTGGTGCACGGCCAGACTGGGATGCTTCTCACAGGCTCCAATGCCAA
AGCCCAGGACCTCGGAACCATCCCCGGCGATTGCGGCTGCCCCTATGTCT
ACAAAAAGGGCAACACCTGGGTTGTGATTGGTGTGCACGTGGCGGCCACC
AGGTCGGGGAACACGGTGATCGCTGCTACCCATGGGGAACCCACCCTTGA
GGCGCTGGAGTTCCAAGGACCCCCCATGCTCCCTCGTCCCTCTGGCACCT
ATGTAGGCCTCCCCATTGCAGACTATGGTGATGCCCCTCCCCTCAGCACC
AAGACCATGTTCTGGCGCACTTCTCCAGAGAAGCTGCCGCCTGGGGCATG
GGAGCCCGCTTACCTGGGTTCCAAGGATGAGAGGGTGGATGGTCCCTCCC
TCCAGCAGGTTATGCGAGACCAGCTCAAGCCCTACTCCGAGCCTCGTGGA
YTACTCCCCCCGCAGGAGATTTTGGACGCAGTTTGTGATGCCATCGAGAA
CCGACTTGAGAATACGCTGGAGCCCCAGAAGCCCTGGACCTTTAAGAAAG
CCTGCGAGAGCCTTGATAAGAACACTAGCAGTGGCTATCCCTACCACAAA
CAGAAGAGTAGGGACTGGACGGGCACCGCCTTCATTGGTGAGCTCGGTGA
TCAAGCCACCCATGCCAACAAC
>303_ColF2
TTCGGCACGGGATGGGGCTTTTGGGTTAGCGGCCATGTGTTCATCACCGC
TAAACATGTGGCGCCACCCAAGGGCACCGAGGTGTTCGGGCGCAAGCCCG
AGGAATTCACCGTCACCTCCAGTGGGGACTTCCTGAAATACCACTTCACC
AGTGCTGTGAGGCCTGACATTCCTGCCATGGTTCTTGAGAATGGCTGCCA
GGAGGGTGTTGTTGCTTCGGTCCTCGTCAAGAGGGCCTCCGGAGAGATGC
TCGCCCTGGCGGTGAGGATGGGTTCTCAGGCCGCCATCAAGATCGGCAAC
GCAGTGGTGCACGGCCAGACTGGGATGCTTCTCACAGGCTCCAATGCCAA
AGCCCAGGACCTCGGAACCATCCCCGGCGATTGCGGCTGCCCCTATGTCT
ACAAAAAGGGCAACACCTGGGTTGTGATTGGTGTGCACGTGGCGGCCACC
AGGTCGGGGAACACGGTGATCGCTGCTACCCATGGGGAACCCACCCTTGA
GGCGCTGGAGTTCCAAGGACCCCCCATGCTCCCTCGTCCCTCTGGCACCT
ATGTAGGCCTCCCCATTGCAGACTATGGTGATGCCCCTCCCCTCAGCACC
AAGACCATGTTCTGGCGCACTTCTCCAGAGAAGCTGCCGCCTGGGGCATG
GGAGCCCGCTTACCTGGGTTCCAAGGATGAGAGGGTGGACGGTCCCTCCC
TCCAGCAGGTTATGCGAGACCAGCTCAAGCCCTACTCCGAGCCTCGTGGA
TTACTCCCCCCGCAGGAGATTTTGGACGCAGTTTGTGATGCCATCGAGAA
CCGACTTGAGAATACGCTGGAGCCCCAGAAGCCCTGGACCTTTAAGAAAG
CCTGCGAGAGCCTTGATAAGAACACTAGCAGTGGCTATCCCTACCACAAA
CAGAAGAGTAGGGACTGGACGGGCACCGCCTTCATTGGTGAGCTCGGTGA
TCAAGCCACCCATGCCAACAAC
>303_MLF2
TTCGGCACGGGATGGGGCTTTTGGGTTAGCGGCCATGTGTTCATCACCGC
TAAACATGTGGCGCCACCCAAGGGCACCGAGGTGTTCGGGCGCAAGCCCG
AGGAATTCACCGTCACCTCCAGTGGGGACTTCCTGAAATACCACTTCACC
AGTGCTGTGAGGCCTGACATTCCTGCCATGGTTCTTGAGAATGGCTGCCA
GGAGGGTGTTGTTGCTTCGGTCCTCGTCAAGAGGGCCTCCGGAGAGATGC
TCGCCCTGGCGGTGAGGATGGGTTCTCAGGCCGCCATCAAGATCGGCAAC
GCAGTGGTGCACGGCCAGACTGGGATGCTTCTCACAGGCTCCAATGCCAA
AGCCCAGGACCTCGGAACCATCCCCGGCGATTGCGGCTGCCCCTATGTCT
ACAAAAAGGGCAACACCTGGGTTGTGATTGGTGTGCACGTGGCGGCCACC
AGGTCGGGGAACACGGTGATCGCTGCTACCCATGGGGAACCCACCCTTGA
GGCGCTGGAGTTCCAAGGACCCCCCATGCTCCCTCGTCCCTCTGGCACCT
ATGTAGGCCTCCCCATTGCAGACTATGGTGATGCCCCTCCCCTCAGCACC
AAGACCATGTTCTGGCGCACTTCTCCAGAGAAGCTGCCGCCTGGGGCATG
GGAGCCCGCTTACCTGGGTTCCAAGGATGAGAGGGTGGACGGTCCCTCCC
TCCAGCAGGTTATGCGAGACCAGCTCAAGCCCTACTCCGAGCCTCGTGGA
TTACTCCCCCCGCAGGAGATTTTGGACGCAGTTTGTGATGCCATCGAGAA
CCGACTTGAGAATACGCTGGAGCCCCAGAAGCCCTGGACCTTTAAGAAAG
CCTGCGAGAGCCTTGATAAGAACACTAGCAGTGGCTATCCCTACCACAAA
CAGAAGAGTAGGGACTGGACGGGCACCGCCTTCATTGGTGAGCTCGGTGA
TCAAGCCACCCATGCCAACAAC
>304_M7F2
TTCGGCACGGGATGGGGCTTTTGGGTTAGCGGCCATGTGTTCATCACCGC
TAAACATGTGGCGCCACCCAAGGGCACCGAGGTGTTCGGGCGCAAGCCCG
AGGAATTCACCGTCACCTCCAGTGGGGACTTCCTGAAATACCACTTCACC
AGTGCTGTGAGGCCTGACATTCCTGCCATGGTTCTTGAGAATGGCTGCCA
GGAGGGTGTTGTTGCTTCGGTCCTCGTCAAGAGGGCCTCCGGAGAGATGC
TCGCCCTGGCGGTGAGGATGGGTTCTCAGGCCGCCATCAAGATCGGCAAC
GCAGTGGTGCACGGCCAGACTGGGATGCTTCTCACAGGCTCCAATGCCAA
AGCCCAGGACCTCGGAACCATCCCCGGCGATTGCGGCTGCCCCTATGTCT

ACAAAAAGGGCAACACCTGGGTTGTGATTGGTGTGCACGTGGCGGCCACC
AGGTCGGGGAACACGGTGATCGCTGCTACCCATGGGGAACCCACCCTTGA
GGCGCTGGAGTTCCAAGGACCCCCCATGCTCCCTCGTCCCTCTGGCACCT
ATGTAGGCCTCCCCATTGCAGACTATGGTGATGCCCCTCCCCTCAGCACC
AAGACCATGTTCTGGCGCACTTCTCCAGAGAAGCTGCCGCCTGGGGCATG
GGAGCCCGCTTACCTGGGTTCCAAGGATGAGAGGGTGGACGGTCCCTCCC
TCCAGCAGGTTATGCGAGACCAGCTCAAGCCCTACTCCGAGCCTCGTGGA
CTACTCCCCCCGCAGGAGATTTTGGATGCAGTTTGTGATGCCATCGAGAA
CCGACTTGAGAATACGCTGGAGCCCCAGAAGCCCTGGACCTTTAAGAAAG
CCTGCGAGAGCCTTGATAAGAACACTAGCAGTGGCTATCCCTACCACAAA
CAGAAGAGTAGGGACTGGACGGGCACCGCCTTCATTGGTGAGCTCGGTGA
TCAAGCCACCCATGCCAACAAC
>304_CaeF2
TTCGGCACGGGATGGGGCTTTTGGGTTAGCGGCCATGTGTTCATCACCGC
TAAACATGTGGCGCCACCCAAGGGCACCGAGGTGTTCGGGCGCAAGCCCG
AGGAATTCACCGTCACCTCCAGTGGGGACTTCCTGAAATACCACTTCACC
AGTGCTGTGAGGCCTGACATTCCTGCCATGGTTCTTGAGAATGGCTGCCA
GGAGGGTGTTGTTGCTTCGGTCCTCGTCAAGAGGGCCTCCGGAGAGATGC
TCGCCCTGGCGGTGAGGATGGGTTCTCAGGCCGCCATCAAGATCGGCAAC
GCAGTGGTGCACGGCCAGACTGGGATGCTTCTCACAGGCTCCAATGCCAA
AGCCCAGGACCTCGGAACCATCCCCGGCGATTGCGGCTGCCCCTATGTCT
ACAAAAAGGGCAACACCTGGGTTGTGATTGGTGTGCACGTGGCGGCCACC
AGGTCGGGGAACACGGTGATCGCTGCTACCCATGGGGAACCCACCCTTGA
GGCGCTGGAGTTCCAAGGACCCCCCATGCTCCCTCGTCCCTCTGGCACCT
ATGTAGGCCTCCCCATTGCAGACTATGGTGATGCCCCTCCCCTCAGCACC
AAGACCATGTTCTGGCGCACTTCTCCAGAGAAGCTGCCGCCTGGGGCATG
GGAGCCCGCTTACCTGGGTTCCAAGGATGAGAGGGTGGACGGTCCCTCCC
TCCAGCAGGTTATGCGAGAYCAGCTCAAGCCCTACTCCGAGCCTCGTGGA
CTACTCCCCCCGCAGGAGATTTTGGATGCAGTTTGTGATGCCATCGAGAA
CCGACTTGAGAATACGCTGGAGCCCCAGAAGCCCTGGACCTTTAAGAAAG
CCTGCGAGAGCCTTGATAAGAACACTAGCAGTGGCTATCCCTACCACAAA
CAGAAGAGTAGGGACTGGACGGGCACCGCCTTCATTGGTGAGCTCGGTGA
TCAAGCCACCCATGCCAACAAC
>305_M7F2
TTCGGCACGGGATGGGGCTTTTGGGTTAGCGGCCATGTGTTCATCACCGC
TAAACATGTGGCGCCACCCAAGGGCACCGAGGTGTTCGGGCGCAAGCCCG
AGGAATTCACCGTCACCTCCAGTGGGGACTTCCTGAAATACCACTTCACC
AGTGCTGTGAGGCCTGACATTCCTGCCATGGTTCTTGAGAATGGCTGCCA
GGAGGGTATTGTTGCTTCGGTCCTCGTCAAGAGGGCCTCCGGAGAGATGC
TCGCCCTGGCGGTGAGGATGGGTTCTCAGGCCGCCATCAAGATCGGCAAC
GCAGTGGTGCACGGCCAGACTGGGATGCTTCTCACAGGCTCCAATGCCAA
AGCCCAGGACCTCGGAACCATCCCCGGCGATTGCGGCTGCCCCTATGTCT
ACAAAAAGGGCAACACCTGGGTTGTGATTGGTGTGCACGTGGCRGCCACC
AGGTCGGGGAACACGGTGATCGCTGCTACCCATGGGGAACCCACCCTTGA
GGCGCTGGAGTTCCAAGGACCCCCCATGCTCCCTCGTCCCTCTGGCACCT
ATGTAGGCCTCCCCATTGCAGACTATGGTGATGCCCCTCCCCTCAGCACC
AAGACCATGTTCTGGCGCACTTCTCCAGAGAAGCTGCCGCCTGGGGCATG
GGAGCCCGCTTACCTGGGTTCCAAGGATGAGAGGGTGGACGGTCCCTCCC
TCCAGCAGGTTATGCGAGACCAGCTCAAGCCCTACTCCGAGCCTCGTGGA
CTACTCCCCCCGCAGGAGATTTTGGACGCAGTTTGTGATGCCATTGAGAA
CCGACTTGAGAATACGCTGGAGCCCCAGAAGCCCTGGACCTTTAAGAAAG
CCTGCGAGAGCCTTGATAAGAACACTAGCAGTGGCTATCCCTACCACAAA
CAGAAGAGTAGGGACTGGACGGGCACCGCCTTCATTGGTGAGCTCGGTGA
TCAAGCCACCCATGCCAACAAC
>305_ColF2
TTCGGCACGGGATGGGGCTTTTGGGTTAGCGGCCATGTGTTCATCACCGC
TAAACATGTGGCGCCACCCAAGGGCACCGAGGTGTTCGGGCGCAAGCCCG
AGGAATTCACCGTCACCTCCAGTGGGGACTTCCTGAAATACCACTTCACC
AGTGCTGTGAGGCCTGACATTCCTGCCATGGTTCTTGAGAATGGCTGCCA
GGAGGGTATTGTTGCTTCGGTCCTCGTCAAGAGGGCCTCCGGAGAGATGC
TCGCCCTGGCGGTGAGGATGGGTTCTCAGGCCGCCATCAAGATCGGCAAC
GCAGTGGTGCACGGCCAGACTGGGATGCTTCTCACAGGCTCCAATGCCAA
AGCTCAGGACCTCGGAACCATCCCCGGCGATTGCGGCTGCCCCTATGTCT
ACAAAAAGGGCAACACCTGGGTTGTGATTGGTGTGCACGTGGCRGCCACC
AGGTCGGGGAACACGGTGATCGCTGCTACCCATGGGGAACCCACCCTTGA
GGCGCTGGAGTTCCAAGGACCCCCCATGCTCCCTCGTCCCTCTGGCACCT
ATGTAGGCCTCCCCATTGCAGACTATGGTGATGCCCCTCCCCTCAGCACC
AAGACCATGTTCTGGCGCACTTCTCCAGAGAAGCTGCCGCCTGGGGCATG
GGAGCCCGCTTACCTGGGTTCCAAGGATGAGAGGGTGGACGGTCCCTCCC

TCCAGCAGGTTATGCGAGACCAGCTCAAGCCCTACTCCGAGCCTCGTGGA
CTACTCCCCCCGCAGGAGATTTTGGACGCAGTTTGTGATGCCATTGAGAA
CCGACTTGAGAATACGCTGGAGCCCCAGAAGCCCTGGACCTTTAAGAAAG
CCTGCGAGAGTCTTGATAAGAACACTAGCAGTGGCTATCCCTACCACAAA
CAGAAGAGTAGGGACTGGACGGGCACCGCCTTCATTGGTGAGCTCGGTGA
TCAAGCCACCCATGCCAACAAC
>306_M5F2
TTCGGCACGGGATGGGGCTTTTGGGTTAGCGGCCATGTGTTCATCACCGC
TAAACATGTGGCGCCACCCAAGGGCACCGAGGTGTTCGGGCGCAAGCCCG
AGGAATTCACCGTCACCTCCAGYGGGGACTTCCTGAAATACCACTTCACC
AGTGCTGTGAGGCCTGACATTCCTGCCATGGTTCTTGAGAATGGCTGCCA
GGAGGGTGTTGTTGCTTCGGTCCTCGTCAAGAGGGCCTCCGGAGAGATGC
TCGCCCTGGCGGTGAGGATGGGTTCTCAGGCCGCCATCAAGATCGGCAAC
GCAGTGGTGCACGGCCAGACTGGGATGCTTCTCACAGGCTCCAATGCCAA
AGCCCAGGACCTCGGAACCATCCCCGGCGATTGCGGCTGCCCCTATGTCT
ACAAAAAGGGCAACACCTGGGTTGTGATTGGTGTGCACGTGGCGGCCACC
AGGTCGGGGAACACGGTGATCGCTGCTACCCATGGGGAACCCACCCTTGA
GGCGCTGGAGTTCCAAGGACCCCCCATGCTCCCTCGTCCCTCTGGCACCT
ATGTAGGCCTCCCCATTGCAGACTATGGTGATGCCCCTCCCCTCAGCACC
AAGACCATGTTCTGGCGCACTTCTCCAGAGAAGCTGCCGCCTGGGGCATG
GGAGCCCGCTTACCTGGGTTCCAAGGATGAGAGGGTGGACGGTCCCTCCC
TCCAGCAGGTTATGCGAGACCAGCTCAAGCCCTACTCCGAGCCTCGTGGA
YTACTCCCCCCGCAGGAGATTTTGGACGCAGTTTGTGATGCCATCGAGAA
CCGACTTGAGAATACGCTGGAGCCCCAGAAGCCCTGGACCTTTAAGAAAG
CCTGCGAGAGCCTTGATAAGAACACTAGCAGTGGCTATCCCTACCACAAA
CAGAAGAGTAGGGACTGGACGGGCACCGCCTTCATTGGTGAGCTCGGTGA
TCAAGCCACCCATGCCAACAAC
>306_M7F2
TTCGGCACGGGATGGGGCTTTTGGGTTAGCGGCCATGTGTTCATCACCGC
TAAACATGTGGCGCCACCCAAGGGCACCGAGGTGTTCGGGCGCAAGCCCG
AGGAATTCACCGTCACCTCCAGTGGGGACTTCCTGAAATACCACTTCACC
AGTGCTGTGAGGCCTGACATTCCTGCCATGGTTCTTGAGAATGGCTGCCA
GGAGGGTGTTGTTGCTTCGGTCCTCGTCAAGAGGGCCTCCGGAGAGATGC
TCGCCCTGGCGGTGAGGATGGGTTCTCAGGCCGCCATCAAGATCGGCAAC
GCAGTGGTGCACGGCCAGACTGGGATGCTTCTCACAGGCTCCAATGCCAA
AGCCCAGGACCTCGGAACCATCCCCGGCGATTGCGGCTGCCCCTATGTCT
ACAAAAAGGGCAACACCTGGGTTGTGATTGGTGTGCACGTGGCGGCCACC
AGGTCGGGGAACACGGTGATCGCTGCTACCCATGGGGAACCCACCCTTGA
GGCGCTGGAGTTCCAAGGACCCCCCATGCTCCCTCGTCCCTCTGGCACCT
ATGTAGGCCTCCCCATTGCAGACTATGGTGATGCCCCTCCCCTCAGCACC
AAGACCATGTTCTGGCGCACTTCTCCAGAGAAGCTGCCGCCTGGGGCATG
GGAGCCCGCTTACCTGGGTTCCAAGGATGAGAGGGTGGACGGTCCCTCCC
TCCAGCAGGTTATGCGAGACCAGCTCAAGCCCTACTCCGAGCCTCGTGGA
CTACTCCCCCCGCAGGAGATTTTGGATGCAGTTTGTGATGCCATCGAGAA
CCGACTTGAGAATACGCTGGAGCCCCAGAAGCCCTGGACCTTTAAGAAAG
CCTGCGAGAGCCTTGATAAGAACACTAGCAGTGGCTATCCCTACCACAAA
CAGAAGAGTAGGGACTGGACGGGCACCGCCTTCATTGGTGAGCTCGGTGA
TCAAGCCACCCATGCCAACAAC
>306_M8F2
TTCGGCACGGGATGGGGCTTTTGGGTTAGCGGCCATGTGTTCATCACCGC
TAAACATGTGGCGCCACCCAAGGGCACCGAGGTGTTCGGGCGCAAGCCCG
AGGAATTCACCGTCACCTCCAGTGGGGACTTCCTGAAATACCACTTCACC
AGTGCTGTGAGGCCTGACATTCCTGCCATGGTTCTTGAGAATGGCTGCCA
GGAGGGTGTTGTTGCTTCGGTCCTCGTCAAGAGGGCCTCCGGAGAGATGC
TCGCCCTGGCGGTGAGGATGGGTTCTCAGGCCGCCATCAAGATCGGCAAC
GCAGTGGTGCACGGCCAGACTGGGATGCTTCTCACAGGCTCCAATGCCAA
AGCCCAGGACCTCGGAACCATCCCCGGCGATTGCGGCTGCCCCTATGTCT
ACAAAAAGGGCAACACCTGGGTTGTGATTGGTGTGCACGTGGCGGCCACC
AGGTCGGGGAACACGGTGATCGCTGCTACCCATGGGGAACCCACCCTTGA
GGCGCTGGAGTTCCAAGGACCCCCCATGCTCCCTCGTCCCTCTGGCACCT
ATGTAGGCCTCCCCATTGCAGACTATGGTGATGCCCCTCCCCTCAGCACC
AAGACCATGTTCTGGCGCACTTCTCCAGAGAAGCTGCCGCCTGGGGCATG
GGAGCCCGCTTACCTGGGTTCCAAGGATGAGAGGGTGGACGGTCCCTCCC
TCCAGCAGGTTATGCGAGACCAGCTCAAGCCCTACTCCGAGCCTCGTGGA
CTACTCCCCCCGCAGGAGATTTTGGATGCAGTTTGTGATGCCATCGAGAA
CCGACTTGAGAATACGCTGGAGCCCCAGAAGCCCTGGACCTTTAAGAAAG
CCTGCGAGAGCCTTGATAAGAACACTAGCAGTGGCTATCCCTACCACAAA
CAGAAGAGTAGGGACTGGACGGGCACCGCCTTCATTGGTGAGCTCGGTGA
TCAAGCCACCCATGCCAACAAC

>306_CaeF2
TTCGGCACGGGATGGGGCTTTTGGGTTAGCGGCCATGTGTTCATCACCGC
TAAACATGTGGCGCCACCCAAGGGCACCGAGGTGTTCGGGCGCAAGCCCG
AGGAATTCACCGTCACCTCCAGTGGGGACTTCCTGAAATACCACTTCACC
AGTGCTGTGAGGCCTGACATTCCTGCCATGGTTCTTGAGAATGGCTGCCA
GGAGGGTGTTGTTGCTTCGGTCCTCGTCAAGAGGGCCTCCGGAGAGATGC
TCGCCCTGGCGGTGAGGATGGGTTCTCAGGCCGCCATCAAGATCGGCAAC
GCAGTGGTGCACGGCCAGACTGGGATGCTTCTCACAGGCTCCAATGCCAA
AGCCCAGGACCTCGGAACCATCCCCGGCGATTGCGGCTGCCCCTATGTCT
ACAAAAAGGGCAACACCTGGGTTGTGATTGGTGTGCACGTGGCGGCCACC
AGGTCGGGGAACACGGTGATCGCTGCTACCCATGGGGAACCCACCCTTGA
GGCGCTGGAGTTCCAAGGACCCCCCATGCTCCCTCGTCCCTCTGGCACCT
ATGTAGGCCTCCCCATTGCAGACTATGGTGATGCCCCTCCCCTCAGCACC
AAGACCATGTTCTGGCGCACTTCTCCAGAGAAGCTGCCGCCTGGGGCATG
GGAGCCCGCTTACCTGGGTTCCAAGGATGAGAGGGTGGACGGTCCCTCCC
TCCAGCAGGTTATGCGAGACCAGCTCAAGCCCTACTCCGAGCCTCGTGGA
CTACTCCCCCCGCAGGAGATTTTGGATGCAGTTTGTGATGCCATCGAGAA
CCGACTTGAGAATACGCTGGAGCCCCAGAAGCCCTGGACCTTTAAGAAAG
CCTGCGAGAGCCTTGATAAGAACACTAGCAGTGGCTATCCCTACCACAAA
CAGAAGAGTAGGGACTGGACGGGCACCGCCTTCATTGGTGAGCTCGGTGA
TCAAGCCACCCATGCCAACAAC
>306_ColF2
TTCGGCACGGGATGGGGCTTTTGGGTTAGCGGCCATGTGTTCATCACCGC
TAAACATGTGGCGCCACCCAAGGGCACCGAGGTGTTCGGGCGCAAGCCCG
AGGAATTCACCGTCACCTCCAGTGGGGACTTCCTGAAATACCACTTCACC
AGTGCTGTGAGGCCTGACATTCCTGCCATGGTTCTTGAGAATGGCTGCCA
GGAGGGTGTTGTTGCTTCGGTCCTCGTCAAGAGGGCCTCCGGAGAGATGC
TCGCCCTGGCGGTGAGGATGGGTTCTCAGGCCGCCATCAAGATCGGCAAC
GCAGTGGTGCACGGCCAGACTGGGATGCTTCTCACAGGCTCCAATGCCAA
AGCCCAGGACCTCGGAACCATCCCCGGCGATTGCGGCTGCCCCTATGTCT
ACAAAAAGGGCAACACCTGGGTTGTGATTGGTGTGCACGTGGCGGCCACC
AGGTCGGGGAACACGGTGATCGCTGCTACCCATGGGGAACCCACCCTTGA
GGCGCTGGAGTTCCAAGGACCCCCCATGCTCCCTCGTCCCTCTGGCACCT
ATGTAGGCCTCCCCATTGCAGACTATGGTGATGCCCCTCCCCTCAGCACC
AAGACCATGTTCTGGCGCACTTCTCCAGAGAAGCTGCCGCCTGGGGCATG
GGAGCCCGCTTACCTGGGTTCCAAGGATGAGAGGGTGGACGGTCCCTCCC
TCCAGCAGGTTATGCGAGACCAGCTCAAGCCCTACTCCGAGCCTCGTGGA
CTACTCCCCCCGCAGGAGATTTTGGATGCAGTTTGTGATGCCATCGAGAA
CCGACTTGAGAATACGCTGGAGCCCCAGAAGCCCTGGACCTTTAAGAAAG
CCTGCGAGAGCCTTGATAAGAACACTAGCAGTGGCTATCCCTACCACAAA
CAGAAGAGTAGGGACTGGACGGGCRCCGCCTTCATTGGTGAGCTCGGTGA
TCAAGCCACCCATGCCAACAAC
